# Pathogen Pursuit: A Gamified Format to Learn Infectious Diseases and Antimicrobial Stewardship for Medical Residents

**DOI:** 10.15766/mep_2374-8265.11565

**Published:** 2025-12-16

**Authors:** Ryan Salemme, Reema Kola, Shazia Samanani

**Affiliations:** 1 Assistant Professor, Department of Medicine, Johns Hopkins School of Medicine; 2 Fellow, Cardiovascular Disease Fellowship Program, Orlando Health; 3 Assistant Professor, Department of Medicine, The George Washington University School of Medicine

**Keywords:** Infectious Disease, Internal Medicine, Games

## Abstract

**Introduction:**

Game-based learning in medical education has emerged as a preferred format for learning complicated topics. At our institution, infectious diseases has been found to be a challenging topic for learners, making it important to find an engaging method to teach this topic. We designed a gamified approach to teach infectious disease management and antimicrobial stewardship for internal medicine resident education.

**Methods:**

In our gamified learning session, participants played a 60–120–minute game with the goal of treating various pathogens. Participants earned Antimicrobial cards, representing common antimicrobial treatments, by correctly answering challenging infectious disease questions, and then matched the Antimicrobial cards to Pathogen cards, to select appropriate treatments for each infectious disease pathogen or organism while also learning about antimicrobial stewardship. Participants completed a postgame assessment using a Likert scale to gauge satisfaction. Knowledge retention was assessed by a pre- and postgame five-question test.

**Results:**

Thirty residents participated in the session. Resident response to the game was positive, with many comments noting its engaging utility to learn such complicated topics. All participants *agreed* or *strongly agreed* that the educational objectives were met and that the format of the game encouraged participants to be engaged. Pre- and postgame five-question analysis showed a 28% increase in mean knowledge scores postgame.

**Discussion:**

Our gamified session was received as an engaging way to learn about complex and complicated infectious disease treatment topics for residents. Our evaluation demonstrated participants were able to a recall information that was covered during the game.

## Educational Objectives

By the end of the session, learners will be able to:

1. Apply knowledge of several common infectious disease topics using a gamified, team-based format.
2. Develop appropriate treatment plans for various infectious disease pathogens commonly seen in internal medicine.
3. Demonstrate principles of antimicrobial stewardship when selecting treatments for various infectious diseases.

## Introduction

**Figure 1.**
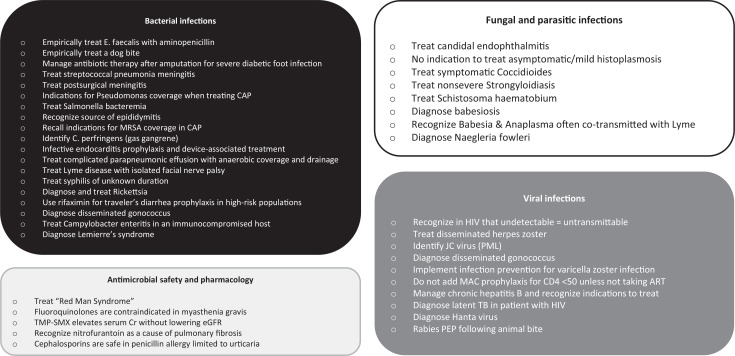
Infectious disease topics and treatments covered in the educational card game Pathogen Pursuit. Abbreviations: E. faecalis, *Enterococcus faecalis*; ART, antiretroviral therapy; CAP, community-acquired pneumonia; Cr, creatinine; eGFR, estimated glomerular filtration rate; JC, John Cunninham virus; MAC, *Mycobacterium avium* complex; MRSA, Methicillin-resistant *Staphylococcus aureus*; PEP, postexposure prophylaxis; PML, progressive multifocal leukoencephalopathy; TB, tuberculosis; TMP-SMX, trimethoprim sulfamethoxazole.

Infectious diseases and antimicrobial stewardship are integral components in medical education, regardless of specialty. Infectious diseases comprises a large proportion of inpatient cases in internal medicine, and is a topic considered high yield in American Board of Internal Medicine review questions. Further, with the rise of multidrug-resistant (MDR) organisms because of antimicrobial misuse and overuse, antimicrobial stewardship is ever more imperative.^1^ In general, we recognize a need for further learning innovations to strengthen training in this topic. At our institution, infectious diseases remains a lower-scoring topic on the in-training exam (ITE) and is cited by learners as challenging to grasp. Our goal was to develop an engaging method to increase internal medicine residents’ exposure to commonly seen infectious disease topics, allowing them to learn appropriate treatments for common infections while applying principles of antimicrobial stewardship (Figure 1).

To minimize reliance on traditional educational formats (eg, lecture-based learning), medical educators have adopted teaching formats that include active learning. As one format, game-based learning, or gamified learning, has emerged as an interactive approach to teach complex topics.^2^ Gamified learning has been shown to improve motivation, participation, and time investment.^3^ There are several high-quality, reproducible published games intended to teach learners different topics, such as the Bloody Board Game, Pulmonopoly, and Candy Gland.^4–6^ There was even a recently published game called Empiric that used gamified learning to teach antimicrobial stewardship at the postresidency level in pediatrics.^7^ Other studies have expanded the use of games to enhance critical thinking skills in an escape room format^8^ or to review teaching points from real cases on rounds during the setting of the COVID-19 pandemic.^9^ Various studies have shown an increase in learner satisfaction with gamified sessions, as well as data supporting that the game-based format is more effective at achieving educational objectives than conventional teaching.^10–15^ For example, a randomized controlled study demonstrated an increase in medical student test scores when they were taught the pharmacology of antimicrobial drugs through a board game versus a traditional lecture-based format.^15^

There are numerous publications in which the benefits of gamification to teach learners about antimicrobials and infectious diseases have been described; however, these studies were largely limited to a single topic within infectious diseases. with most cases targeted at the medical student learner, attending level learner, or nonphysician health care worker.^7,16–22^ Few resources exist to teach internal medicine residents infectious diseases topics through a gamified format. We designed an educational curriculum using a widely accessible gamified format to teach and review broad and complex infectious diseases topics and treatments, which is high yield in terms of applying the knowledge in real-world clinical practice. As such, our approach introduces a wider-scoped active learning tool for the postgraduate learner in infectious diseases.

## Methods

While developing our didactic curriculum, we began with an informal needs assessment by reviewing prior scores on the resident ITEs. We found that our residents uniformly scored low on infectious diseases. This was followed by a literature review aimed at improving efficacy of teaching infectious diseases, with findings showing that gamified learning results in better engagement and retention.^2,3,23^ We then sought to apply this method of learning to teach our residents complex infectious disease topics. Our educational chief resident and associate program director of curriculum development developed our teaching game based on American Board of Internal Medicine and ITE infectious disease subtopics (Figure 1), using Kern's approach to curriculum development.^24^ These subtopics were chosen mostly from the lowest-scoring subtopics by percentile on the ITE, with some other topics included that we noticed residents had difficulty answering during weekly board review sessions. We then conducted an extensive literature review based on these topics, including primary and secondary infectious diseases literature as well as major guidelines, to create original, up-to-date, board-style questions on these topics for incorporation into the curriculum (Appendix A). Thereafter, we created Antimicrobial and Pathogen cards that represent the antimicrobial treatments and infectious disease pathogens most commonly encountered at our hospital on the internal medicine wards as well as in internal medicine board review questions.

**Figure 2.**
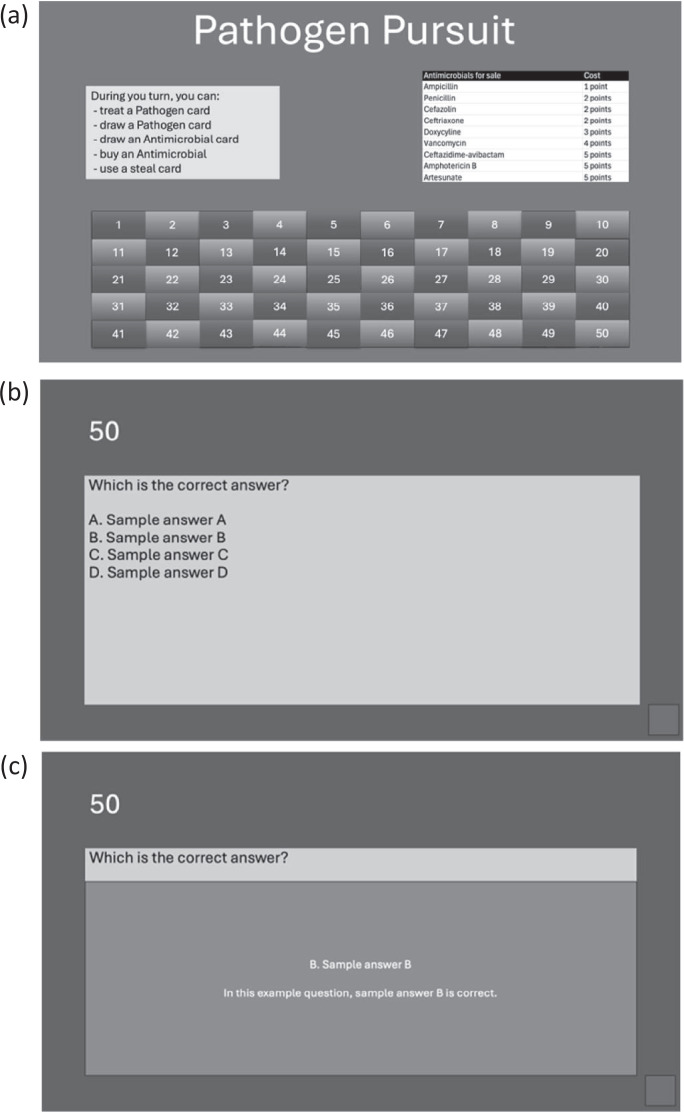
The Pathogen Pursuit game board (a), sample question (b), and sample answer slide (c) with an explanation.

We designed a card-based game, Pathogen Pursuit, to teach residents how to treat several infectious disease pathogens (Appendix B). The game was designed to allow variability in timing, such that a game can last from 60 minutes up to 120 minutes. This game was intended for up to 20 participants divided into four or five teams, with one or two instructors. At a minimum, the game can be played with three participants and one instructor. The game consisted of two sets of cards: (1) 50 unique Pathogen cards that either include a specific disease, such as community acquired pneumonia (outpatient with no comorbidities), or an organism, such as gonorrhea (Appendix C); and (2) 35 unique Antimicrobial cards (Appendix D) used to match with (ie, treat) the respective Pathogen cards. The team drew an Antimicrobial card and had to correctly answer its corresponding question displayed on the electronic game board (Figure 2; Appendix E). The main game board (Figure 2a) includes instructions on what participants can do during their turn, prices of antimicrobials that are available for sale, and the question grid. When participants drew an Antimicrobial card, the instructor then selected the corresponding number on that card to bring up the questions. Figure 2b is a sample question (note that for instructional purposes, the actual question from the game was omitted). Hitting the space bar reveals the answer and explanation, as shown in Figure 2c. Hitting the button in the bottom-right corner of the slide returns players to the game board.

Each question had its own educational objective (Appendix A). If answered correctly, the team kept their Antimicrobial card, and if they answered incorrectly, they forfeited their turn. After earning Antimicrobial cards, participants earned points by matching Antimicrobial cards to Pathogen cards (ie, treating the pathogen) on subsequent turns. The instructor was given an answer key (Appendix F) to confirm proper antimicrobial treatment (ie, correct match of Antimicrobial card to Pathogen card). Participants could treat overbroadly, for example using meropenem to treat simple cystitis, but then would find themselves later unable to treat MDR organisms that they had acquired, thus encouraging the use of principles of antibiotic stewardship throughout. During the game play, participants might have inadvertently chosen a card designated a wild card, which could be played as any antimicrobial (eg, the Infectious Disease Consult card); the C diff card, which represents a pathogen that participants must treat before being able to continue treating other pathogens; the Vaccine card, which allowed teams to not deduct any points for untreated pathogens at the end of the game; the MDR card, for which 1 point was deducted; the Central Line Associated Blood Stream Infection (CLABSI) card, for which 5 points were deducted; or a Steal card.

Points were tallied throughout the game as teams selected Antimicrobial treatment cards matched with Pathogen cards. At the conclusion of the game, teams added up the unmatched (untreated) Pathogen cards that they still had in hand and subtracted that from their score (the exception to this is if a team had a Vaccine card). The team with the most points won.

We ran the game over two sessions. Participants included 30 internal medicine residents from postgraduate years PGY 1 to PGY 3. Using Kirkpatrick's Model of Evaluation,^25^ we developed an 11-question survey-based assessment to measure resident reaction (Level 1) to the didactic session. The assessment used a 5-point Likert scale to assess utility of and satisfaction with the session, with the scale ranging from 1 = *strongly disagree* to 5 = *strongly agree* (Appendix G). The particular questions were selected to best assess the didactic within Level 1 of Kirkpatrick's Model of Evaluation.^25^ We also added a section to allow open-ended comments from participants on the innovation.

A five-question pregame test was administered immediately before the game, and a postgame test was administered at the end of the session week (Appendix H). Five questions were selected as the intended purpose was formative rather than summative. This was included to assess learning (Level 2). The five-question test included questions chosen at random from the game and was intended to assess knowledge retention while requiring minimal participant time.

The project was submitted to the George Washington University Institutional Review Board and was deemed exempt from further review (No. NCR256329; May 19, 2025).

## Results

All 30 participants completed the survey assessment. For all 11 questions with Likert-scale responses, participant responses averaged between *agree* and *strongly agree* (Table 1). Notably, participants *strongly agreed* that the educational objectives were met (mean score 4.6) and that the gamified session was an appropriate way to learn the material (mean score 4.7). Every single participant *agreed* (5 of 30 responders) or *strongly agreed* (25 of 30 responders) that the format of the game encouraged participants to be engaged. We also separated results of the Likert analysis by PGY 1 and PGY 2/PGY 3 participants to assess whether there was a difference in efficacy of our innovation based on level of training (Table 1). Notably, there was no substantial difference between the two cohorts for any of the questions asked.

**Table 1.**
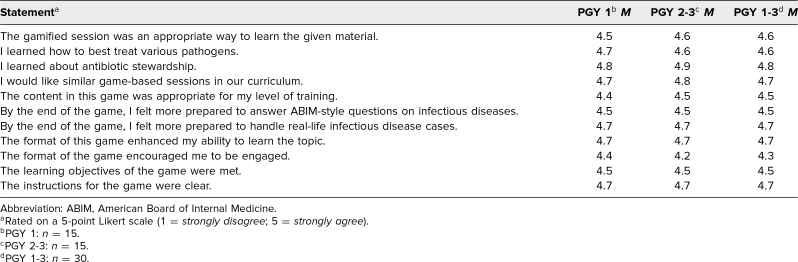
Participant Postgame Evaluation of Session Utility and Satisfaction (*N* = 30)

All 30 participants completed the pre- and postgame knowledge retention tests. There was a 28% increase in the mean knowledge score from pre- to postgame, as participants scored a mean 51% on the pregame test and a mean 79% on the postgame test (Table 2). Despite an overall difference in participants’ knowledge from pre- to postgame between the PGY 1 cohort and the PGY 2/PGY 3 cohort, the percentage increase in mean score was nearly identical, at 28% and 27%, respectively. It was evident that if we did not get to a topic during the game, then there was no increase in knowledge score for the correlating question on the postgame test. Questions covered during the game showed a postgame increase in participants’ knowledge.

**Table 2.**
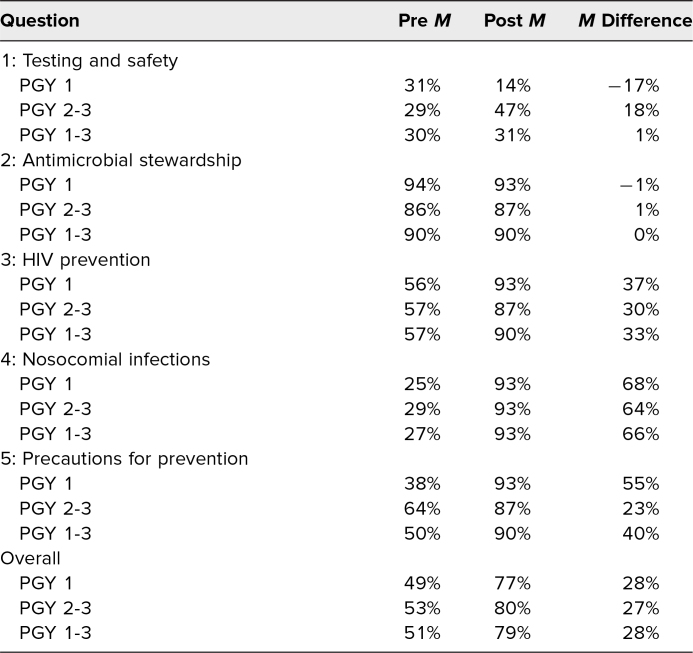
Pre- and Postgame Assessment of Knowledge Retention (*N* = 30)

Open-ended responses from the participants were quite positive. For example, several participants noted that the game was both fun and informative. Other participants described how the game made them more engaged, expressing enjoyment and satisfaction with the format.

## Discussion

Despite being an important, high-yield topic both in real-life clinical practice and in board questions for all learners in medicine, infectious diseases was a lower-scoring category on the ITE at our institution, and many of our learners have cited that it was a challenging subject to learn. We sought to create an engaging innovation to address this while also practicing principles of antimicrobial stewardship. Game-based learning has been shown to be an engaging and enjoyable way to learn, with multiple prior publications showing the benefit in medical education.^2,10,23,26^ Further, team-based learning with an element of competition enhances participation, better integrates the individual, alleviates fear of failure, and improves motivation.^27^ To reflect this, we designed a gamified didactic session to teach and review commonly encountered infectious disease topics in a manner previously shown to be engaging, with our target audience of internal medicine residents.

Strong integration between educational objectives and training goals has been described as a key element for successful game design in medical education.^27^ Our first educational objective was for residents to apply knowledge of several common infectious disease topics using a gamified, team-based format. This objective was addressed through game-based clinical questions that required learners to use clinical reasoning to determine accurate diagnosis and management. We intentionally designed our questions as two-step, or linked, questions using the key feature method, requiring learners to first establish the correct diagnosis from the case and then select the appropriate treatment. This format has been shown to effectively accommodate the complexity often needed to answer difficult clinical questions, and it ensured that residents could not succeed without actively applying their knowledge.^28^ Evidence of achievement was demonstrated by residents’ ability to correctly answer case-based questions and accrue points, reflecting appropriate application of their existing knowledge to novel scenarios.

Our second educational objective was for our learners to develop appropriate treatment plans for various infectious disease pathogens commonly seen in internal medicine. To meet this objective, learners were presented with case-based scenarios in which determining the correct management plan was essential to advancing in the game. These scenarios highlighted common pathogens and required residents to integrate diagnostic reasoning with therapeutic decision-making, ensuring that management choices were evidence-based and guideline-concordant. The achievement of this objective was demonstrated through residents’ ability to consistently select appropriate treatment strategies during game play. Further confirmation was provided by higher postgame knowledge test scores compared to pregame test scores, demonstrating measurable improvement in residents’ ability to construct effective treatment plans through gained knowledge on treatment.

Our final educational objective was for our learners to demonstrate principles of antimicrobial stewardship when selecting treatment for various infectious diseases. This was addressed through the intentional design of the game, in which the availability of certain antimicrobials was limited. Learners quickly discovered through experiential game play that early use of overly broad-spectrum agents left them unable to treat MDR infectious diseases or more severe infection in subsequent rounds. Success in the game therefore required them to apply stewardship principles, such as narrowing coverage and conserving stronger or broader antibiotics for later use. Evidence of achievement was seen by residents’ ability to adapt their treatment strategies over the course of game play.

The gamified learning format was well received by the participants. Subjective assessments showed that participants were satisfied with the session in several areas. The team-based, gamified format was unanimously found to be an appropriate and engaging way to learn the material. Comments from residents also indicated a clear enjoyment in learning through this type of gamified format, with many residents asking for more games to be incorporated in their didactics. While an inherent limitation is that we did not run a control group who learned the material throughout traditional learning methods (ie, lectures), our participants, who have historically participated in several traditional formats, noted that they found this game a more enjoyable and preferred way to learn, and all 30 participants either *agreed* or *strongly agreed* that they would like similar games in the curriculum in future didactics.

We designed the game to be easily reproducible by other institutions with negligible cost. The game cards (Appendix C and D) can be printed, and the electronic game board slide show (Appendix E) is downloadable. We also have included a comprehensive key to assess for successful treatment of pathogens during the game play. We purposefully made all components of the game editable to adjust to any alternative educational objectives or topic matter, allowing other programs to tailor the game to their educational needs. However, any edits made must maintain the rigor and evidence-based nature of the original questions.

There were recognized limitations to our game. Given its complexities, the instructions are somewhat complicated and slightly time consuming to explain to learners; however, nearly all participants (28 of 30) *agreed* or *strongly agreed* that the instructions of the game were clear. Without any modifications, completion of the entire game itself typically takes about 2 hours, which may prohibit its use in shorter didactic sessions. To address this, we include suggested modifications to the instructions, such as allowing participants to choose Antimicrobial cards face up rather than blinded or by removing the viral and fungal Pathogen cards to allow for faster game play while retaining the rigor and ability to achieve the intended educational objectives. The editability of our game also affords the instructor the ability to remove or edit questions that may be more difficult if targeting a different level of learners. We note that from our experience in our didactics, the game was stopped at our time limit and still proved to be a strong learning opportunity even without needing to complete all cards.

As we have designed the game for the advanced learner, we noticed that many of the questions were challenging for the PGY 1 level, and in some cases more appropriate for PGY 2 and PGY 3 residents. A qualitative analysis by Wang et al demonstrated that balancing difficulty with pressure in gamified learning is a crucial element and considered important by many experts.^27^ We recognize that implementing a gamified curriculum that is misaligned with the learner's level of training and knowledge may also be counterproductive, and as described in prior literature, can even result in poor educational outcomes.^23,26^ Despite varying level of difficulty being apparent during our game, this fostered valuable discussion about the many subtopics. A possible modification for early PGY 1 learners includes allowing for an open-book format of game play to lower the difficulty but still allow for overall learning. A future direction would be to create an alternate game e-board with questions aimed at an introductory level, or even to target another cohort, such as medical students.

Our assessment was designed to evaluate outcomes at Kirkpatrick Level 1 (learner reaction) and Level 2 (learning), but not Level 3 (behavior change) or Level 4 (results). This represents a limitation of our study, and future work should aim to incorporate higher-level outcomes to more fully assess the impact of our intervention. A more effective future design would be to ask a higher volume of questions in a unique pregame and postgame test. While it would also be ideal to administer a postgame assessment further in the future rather than at the end of the week, this is limited by a likely drop in willingness to take the assessment, as well as not having the same sample of people who played the game at subsequent didactics. Administering the test at a later date would also introduce the potential for several confounders as some participants may have spent time on an infectious diseases rotation, may have seen certain cases, or may have engaged in supplemental learning. Nevin et al introduced a large-scale gamified learning platform to their internal medicine residents using multiple choice–based questions and were able to demonstrate engagement of a large number of learners as well as acquisition of new knowledge along with retention of this knowledge over time.^3^ In general, we also did not control for factors such as baseline enthusiasm or knowledge of infectious diseases, given the relatively small available sample size.

Several lessons emerged through the creation of this gamified didactic. First, developing a successful gamified lesson is an iterative process, which is necessary to refine content, instruction, and delivery. Another lesson is that assessment development should comprise a substantial component of gamified didactic creation, ensuring alignment with educational objectives as well as the nature of the content being taught. Additionally, thoughtful consideration of learner level is critical to avoid unintended challenges or disengagement, especially when creating gamified learning for multiple levels of learners. Finally, gamified learning requires substantially more effort to design than traditional lecture-based learning methods. However, the level of resident engagement and appreciation can make it a worthwhile endeavor.

We have successfully created a fun and engaging learning tool for increasing students’ understanding of infectious diseases at the advanced learning level by using a unique gamified format. The game was well received by participants and addresses learning outcomes that are crucial to the growth of internal medicine residents who frequently encounter infectious disease topics in both clinical practice and internal medicine boards. The game is easily reproducible at other institutions and can be replicated and incorporated into didactics or modified to accommodate different educational objectives.

## Appendices


Educational Objectives by Quesitons.docxGame Instructions.docxPathogen Game Cards.pdfAntimicrobial Game Cards.pdfGame Board Slide Show.pptxKey.pdfPostgame Survey.docxPre- and Posttest.docx
*All appendices are peer reviewed as integral parts of the Original Publication.*
