# Supplementary material for: Pathogen Pursuit: A Gamified Format to Learn Infectious Diseases and Antimicrobial Stewardship for Medical Residents
Source: MedEdPORTAL. 2025 Dec 16;21:11565. doi: 10.15766/mep_2374-8265.11565 (PMC12705857; doi:10.15766/mep_2374-8265.11565)
Supplement: Supplementary file 1 — Educational Objectives by Quesitons.docxGame Instructions.docxPathogen Game Cards.pdfAntimicrobial Game Cards.pdfGame Board Slide Show.pptxKey.pdfPostgame Survey.docxPre- and Posttest.docx [file mep_2374-8265.11565-s001.zip › A. Educational Objectives by Questions.docx]

**Appendix A:**

Educational Objective by Questions

1. empirically treat E faecalis with an aminopenicillin^1^
2. empirically treat a dog bite^2^
3. manage antibiotic therapy after amputation for severe diabetic foot infection^3^
4. recognize indications for head CT prior to LP^4^
5. treat streptococcal pneumonia meningitis^4^
6. treat Syphilis of unknown duration^5^
7. treat post-surgical meningitis^6^
8. treat patient with Lyme disease and isolated facial nerve palsy^7^
9. recognize babesia and Anaplasma often are transmitted along with Lyme^7-9^
10. indications for Pseudomonas coverage when treating for CAP^10^
11. do not add MAC prophylaxis for CD4 <50 unless patient not expected to take ART^11^
12. recognize in HIV that undetectable equals untransmittable^12^
13. treat Campylobacter enteritis in an immunocompromised host^13^
14. treat Salmonella bacteremia^14^
15. treat severe malaria^15^
16. manage chronic hepatitis B^16^
17. recognize indications to treat hepatitis B^16^
18. treat HCV in almost all cases except short life expectancy^17^
19. recognize source of epididymitis^5,18^
20. recall indications for MRSA coverage in CAP^10^
21. diagnose latent TB in a patient living with HIV^19^
22. diagnose babesiosis^9,20^
23. treat disseminated herpes zoster^21^
24. treat candidal endophthalmitis with vitriol-penetrating anti-fungal^22^
25. no indication to treat asymptomatic or mild histoplasmosis infections^23^
26. treat symptomatic Coccidioides^23^
27. double cover for Pseudomonas in VAP with patient with rick factors for resistance^24^
28. treat “Red Man Syndrome”^25^
29. Fluoroquinolones are contraindicated in myasthenia gravis^26^
30. TMX-sulfamethoxazole results in an elevated serum Cr without lowering eGFR^27^
31. recognize nitrofurantoin as a cause of pulmonary fibrosis^25^
32. cephalosporins are safe in patients with a penicillin allergy limited to urticaria^28^
33. treat pulmonary nocardia^29^
34. diagnose Lemierre’s syndrome^30^
35. post-infectious glomerulonephritis is self-limiting^31^
36. identify C. perfringens the pathogen causing gas gangrene^2^
37. rabies PEP following animal bite^32^
38. identify JC virus as a cause of PML^33^
39. diagnose and treat Rickettsia^9^
40. prophylaxis for infective endocarditis^34^
41. select appropriate prophylaxis for infective endocarditis^34^
42. treat device-associated endocarditis^34,35^
43. treat non-severe Strongyloidiasis^36^
44. treat Schistosoma haematobium^37^
45. diagnose Naegleria fowleri^38^
46. use rifaximin for prophylaxis for traveler’s diarrhea in high-risk populations^19,39^
47. diagnose hanta virus^40^
48. diagnose disseminated gonococcus^5,41^
49. implement infection prevention for varicella zoster infection^42^
50. treat a complicated parapneumonic effusion with anaerobic coverage and drainage^43,44^

**References**

1. Mazuski JE, Tessier JM, May AK, et al. The Surgical Infection Society Revised Guidelines on the Management of Intra-Abdominal Infection. *Surg Infect (Larchmt)*. Jan 2017;18(1):1-76. doi:10.1089/sur.2016.261

2. Stevens DL, Bisno AL, Chambers HF, et al. Practice guidelines for the diagnosis and management of skin and soft tissue infections: 2014 update by the Infectious Diseases Society of America. *Clin Infect Dis*. Jul 15 2014;59(2):e10-52. doi:10.1093/cid/ciu444

3. Senneville E, Albalawi Z, van Asten SA, et al. IWGDF/IDSA guidelines on the diagnosis and treatment of diabetes-related foot infections (IWGDF/IDSA 2023). *Diabetes Metab Res Rev*. Mar 2024;40(3):e3687. doi:10.1002/dmrr.3687

4. Young N, Thomas M. Meningitis in adults: diagnosis and management. *Intern Med J*. Nov 2018;48(11):1294-1307. doi:10.1111/imj.14102

5. Workowski KA, Bachmann LH, Chan PA, et al. Sexually Transmitted Infections Treatment Guidelines, 2021. *MMWR Recomm Rep*. Jul 23 2021;70(4):1-187. doi:10.15585/mmwr.rr7004a1

6. Tunkel AR, Hasbun R, Bhimraj A, et al. 2017 Infectious Diseases Society of America's Clinical Practice Guidelines for Healthcare-Associated Ventriculitis and Meningitis. *Clin Infect Dis*. Mar 15 2017;64(6):e34-e65. doi:10.1093/cid/ciw861

7. Lantos PM, Rumbaugh J, Bockenstedt LK, et al. Clinical Practice Guidelines by the Infectious Diseases Society of America (IDSA), American Academy of Neurology (AAN), and American College of Rheumatology (ACR): 2020 Guidelines for the Prevention, Diagnosis and Treatment of Lyme Disease. *Clin Infect Dis*. Jan 23 2021;72(1):1-8. doi:10.1093/cid/ciab049

8. Horowitz HW, Aguero-Rosenfeld ME, Holmgren D, et al. Lyme disease and human granulocytic anaplasmosis coinfection: impact of case definition on coinfection rates and illness severity. *Clin Infect Dis*. Jan 2013;56(1):93-9. doi:10.1093/cid/cis852

9. Ho BM, Davis HE, Forrester JD, et al. Wilderness Medical Society Clinical Practice Guidelines for the Prevention and Management of Tick-Borne Illness in the United States. *Wilderness Environ Med*. Dec 2021;32(4):474-494. doi:10.1016/j.wem.2021.09.001

10. Metlay JP, Waterer GW, Long AC, et al. Diagnosis and Treatment of Adults with Community-acquired Pneumonia. An Official Clinical Practice Guideline of the American Thoracic Society and Infectious Diseases Society of America. *Am J Respir Crit Care Med*. Oct 1 2019;200(7):e45-e67. doi:10.1164/rccm.201908-1581ST

11. Guidelines for the Prevention and Treatment of Opportunistic Infections in Adults and Adolescents With HIV. *HIV Clinical Guidelines* 2024;

12. Okamoto EE, Anam FR, Batiste S, et al. Ending AIDS as a public health threat: the imperative for clear messaging on U=U, viral suppression, and zero risk. *Lancet HIV*. Nov 2024;11(11):e783-e790. doi:10.1016/S2352-3018(24)00241-8

13. Shane AL, Mody RK, Crump JA, et al. 2017 Infectious Diseases Society of America Clinical Practice Guidelines for the Diagnosis and Management of Infectious Diarrhea. *Clin Infect Dis*. Nov 29 2017;65(12):1963-1973. doi:10.1093/cid/cix959

14. Plumb IF, Patricia; Bruce, Beau. Salmonellosis, Nontyphoidal. *CDC Yellow Book 2024*. 2024;

15. Abanyie F, Acharya SD, Leavy I, Bowe M, Tan KR. Safety and Effectiveness of Intravenous Artesunate for Treatment of Severe Malaria in the United States-April 2019 Through December 2020. *Clin Infect Dis*. Dec 6 2021;73(11):1965-1972. doi:10.1093/cid/ciab570

16. Terrault NA, Lok ASF, McMahon BJ, et al. Update on Prevention, Diagnosis, and Treatment of Chronic Hepatitis B: AASLD 2018 Hepatitis B Guidance. *Clin Liver Dis (Hoboken)*. Jul 2018;12(1):33-34. doi:10.1002/cld.728

17. Ghany MG, Morgan TR, Panel A-IHCG. Hepatitis C Guidance 2019 Update: American Association for the Study of Liver Diseases-Infectious Diseases Society of America Recommendations for Testing, Managing, and Treating Hepatitis C Virus Infection. *Hepatology*. Feb 2020;71(2):686-721. doi:10.1002/hep.31060

18. McConaghy JR, Panchal B. Epididymitis: An Overview. *Am Fam Physician*. Nov 1 2016;94(9):723-726.

19. Masur H, Kaplan JE, Holmes KK, Service USPH, Infectious Diseases Society of A. Guidelines for preventing opportunistic infections among HIV-infected persons--2002. Recommendations of the U.S. Public Health Service and the Infectious Diseases Society of America. *Ann Intern Med*. Sep 3 2002;137(5 Pt 2):435-78. doi:10.7326/0003-4819-137-5_part_2-200209031-00002

20. Waked R, Krause PJ. Human Babesiosis. *Infect Dis Clin North Am*. Sep 2022;36(3):655-670. doi:10.1016/j.idc.2022.02.009

21. Kitaya S, Iyobe R, Kanamori H. Disseminated Herpes Zoster in an Immunocompromised Patient: Challenges for Preventing Transmission Before Diagnosis. *Am J Med*. Oct 15 2024;doi:10.1016/j.amjmed.2024.10.010

22. Pappas PG, Kauffman CA, Andes DR, et al. Clinical Practice Guideline for the Management of Candidiasis: 2016 Update by the Infectious Diseases Society of America. *Clin Infect Dis*. Feb 15 2016;62(4):e1-50. doi:10.1093/cid/civ933

23. Galgiani JN, Kauffman CA. Coccidioidomycosis and Histoplasmosis in Immunocompetent Persons. *N Engl J Med*. Feb 8 2024;390(6):536-547. doi:10.1056/NEJMra2306821

24. Kalil AC, Metersky ML, Klompas M, et al. Management of Adults With Hospital-acquired and Ventilator-associated Pneumonia: 2016 Clinical Practice Guidelines by the Infectious Diseases Society of America and the American Thoracic Society. *Clin Infect Dis*. Sep 1 2016;63(5):e61-e111. doi:10.1093/cid/ciw353

25. Broyles AD, Banerji A, Barmettler S, et al. Practical Guidance for the Evaluation and Management of Drug Hypersensitivity: Specific Drugs. *J Allergy Clin Immunol Pract*. Oct 2020;8(9S):S16-S116. doi:10.1016/j.jaip.2020.08.006

26. Jones SC, Sorbello A, Boucher RM. Fluoroquinolone-associated myasthenia gravis exacerbation: evaluation of postmarketing reports from the US FDA adverse event reporting system and a literature review. *Drug Saf*. Oct 1 2011;34(10):839-47. doi:10.2165/11593110-000000000-00000

27. Yokoyama S, Nakagawa J, Aiuchi N, Seito T, Niioka T. Impact of trimethoprim on serum creatinine, sodium, and potassium concentrations in patients taking trimethoprim-sulfamethoxazole without changes in glomerular filtration rate. *J Clin Pharm Ther*. Sep 2022;47(9):1409-1417. doi:10.1111/jcpt.13679

28. Engelman R, Shahian D, Shemin R, et al. The Society of Thoracic Surgeons practice guideline series: Antibiotic prophylaxis in cardiac surgery, part II: Antibiotic choice. *Ann Thorac Surg*. Apr 2007;83(4):1569-76. doi:10.1016/j.athoracsur.2006.09.046

29. Restrepo A, Clark NM, Infectious Diseases Community of Practice of the American Society of T. Nocardia infections in solid organ transplantation: Guidelines from the Infectious Diseases Community of Practice of the American Society of Transplantation. *Clin Transplant*. Sep 2019;33(9):e13509. doi:10.1111/ctr.13509

30. Carrara A, Bertelli C, Gardiol C, et al. Association of pathogenic determinants of Fusobacterium necrophorum with bacteremia, and Lemierre's syndrome. *Sci Rep*. Aug 27 2024;14(1):19804. doi:10.1038/s41598-024-70608-y

31. Duong MD, Reidy KJ. Acute Postinfectious Glomerulonephritis. *Pediatr Clin North Am*. Dec 2022;69(6):1051-1078. doi:10.1016/j.pcl.2022.08.001

32. Rupprecht CE, Gibbons RV. Clinical practice. Prophylaxis against rabies. *N Engl J Med*. Dec 16 2004;351(25):2626-35. doi:10.1056/NEJMcp042140

33. Ferenczy MW, Marshall LJ, Nelson CD, et al. Molecular biology, epidemiology, and pathogenesis of progressive multifocal leukoencephalopathy, the JC virus-induced demyelinating disease of the human brain. *Clin Microbiol Rev*. Jul 2012;25(3):471-506. doi:10.1128/CMR.05031-11

34. Otto CM, Nishimura RA, Bonow RO, et al. 2020 ACC/AHA Guideline for the Management of Patients With Valvular Heart Disease: A Report of the American College of Cardiology/American Heart Association Joint Committee on Clinical Practice Guidelines. *Circulation*. Feb 2 2021;143(5):e72-e227. doi:10.1161/CIR.0000000000000923

35. Chesdachai S, Esquer Garrigos Z, DeSimone CV, DeSimone DC, Baddour LM. Infective Endocarditis Involving Implanted Cardiac Electronic Devices: JACC Focus Seminar 1/4. *J Am Coll Cardiol*. Apr 9 2024;83(14):1326-1337. doi:10.1016/j.jacc.2023.11.036

36. Buonfrate D, Rodari P, Barda B, Page W, Einsiedel L, Watts MR. Current pharmacotherapeutic strategies for Strongyloidiasis and the complications in its treatment. *Expert Opin Pharmacother*. Oct 2022;23(14):1617-1628. doi:10.1080/14656566.2022.2114829

37. Danso-Appiah A, Garner P, Olliaro PL, Utzinger J. Treatment of urinary schistosomiasis: methodological issues and research needs identified through a Cochrane systematic review. *Parasitology*. Nov 2009;136(13):1837-49. doi:10.1017/S0031182009005939

38. Jahangeer M, Mahmood Z, Munir N, et al. Naegleria fowleri: Sources of infection, pathophysiology, diagnosis, and management; a review. *Clin Exp Pharmacol Physiol*. Feb 2020;47(2):199-212. doi:10.1111/1440-1681.13192

39. Riddle MS, DuPont HL, Connor BA. ACG Clinical Guideline: Diagnosis, Treatment, and Prevention of Acute Diarrheal Infections in Adults. *Am J Gastroenterol*. May 2016;111(5):602-22. doi:10.1038/ajg.2016.126

40. Vial PA, Ferres M, Vial C, et al. Hantavirus in humans: a review of clinical aspects and management. *Lancet Infect Dis*. Sep 2023;23(9):e371-e382. doi:10.1016/S1473-3099(23)00128-7

41. Blank JA, Thapa N, Mansoor AM. Arthritis-Dermatitis Syndrome: a Case of Disseminated Gonococcal Infection with Petechial Skin Rash. *J Gen Intern Med*. Sep 2021;36(9):2836-2838. doi:10.1007/s11606-021-06923-1

42. Siegel JD, Rhinehart E, Jackson M, Chiarello L, Health Care Infection Control Practices Advisory C. 2007 Guideline for Isolation Precautions: Preventing Transmission of Infectious Agents in Health Care Settings. *Am J Infect Control*. Dec 2007;35(10 Suppl 2):S65-164. doi:10.1016/j.ajic.2007.10.007

43. Sultan S, Gupta E, Benzaquen S. Management of complicated parapneumonic effusions. *Curr Opin Pulm Med*. Jan 1 2023;29(1):54-59. doi:10.1097/MCP.0000000000000934

44. Shen KR, Bribriesco A, Crabtree T, et al. The American Association for Thoracic Surgery consensus guidelines for the management of empyema. *J Thorac Cardiovasc Surg*. Jun 2017;153(6):e129-e146. doi:10.1016/j.jtcvs.2017.01.030

Educational objective by questions
