# Supplementary material for: Pathogen Pursuit: A Gamified Format to Learn Infectious Diseases and Antimicrobial Stewardship for Medical Residents
Source: MedEdPORTAL. 2025 Dec 16;21:11565. doi: 10.15766/mep_2374-8265.11565 (PMC12705857; doi:10.15766/mep_2374-8265.11565)
Supplement: Supplementary file 1 — Educational Objectives by Quesitons.docxGame Instructions.docxPathogen Game Cards.pdfAntimicrobial Game Cards.pdfGame Board Slide Show.pptxKey.pdfPostgame Survey.docxPre- and Posttest.docx [file mep_2374-8265.11565-s001.zip › B. Game Instructions.docx]

**Pathogen Pursuit: Game Instructions**

Overview

The objective of the game is to earn as many points as possible by treating various pathogens or infectious diseases. The game is a card-game, electronic board-game hybride: participants use and earn cards that are kept face up, and answer questions on the electronic board game. Participants will be given various “Pathogen” cards that either include a specific disease, such as “Community Acquired Pneumonia (outpatient with no comorbidities),” or just an organism itself like Gonorrhea. Each team is given 3 “Pathogen” cards at the start of the game, and can draw more during each during. To treat the “Pathogens,” participants will use “Antimicrobial” cards either alone or in combination, depending on the disease or pathogen they are treating. To earn Antimicrobial cards, participants will answer a multiple-choice question associated with the card.

Set up/Preparation:

1. This game is intended for 3-5 groups of 3-25 participants in addition to 1-2 instructors.
2. Participants will split into groups of approximately 4-5 learners
3. Each group will randomly be given 3 Pathogen cards

Playing the game:

1. Each team will have a turn to play every round, playing in order by team in a counterclockwise fashion. Cards earned and used are not-blinded and should be kept face-up.
2. For each turn, teams will have the option to do one of the following:
   1. Draw an anti-microbial card of the top of the deck
      1. Once the team draws the antimicrobial card, the instructor will use the PowerPoint to bring up the associated question that corresponds to the number listed on the top left of that antimicrobial card (example below; appendix C). The team has 90-seconds to answer the questions.
         1. If the team answers correctly, the team keeps the antimicrobial card to use at a later turn
         2. If the team answers incorrectly, they forfeit the card, and the card is no longer in play for the rest of the game


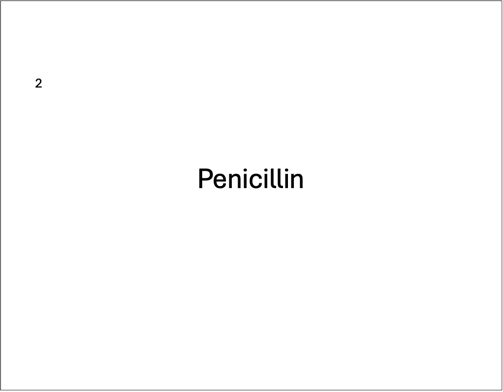


- - - 1. For example, the Antimicrobial card for penicillin is shown here. If drawn, participants would answer question #2 on the corresponding electronic game board in order to keep the card.
    1. Some of the antimicrobial cards are not tied to a question, and either contain a reward or punishment
       1. Steal: This card allows the team to steal an Antimicrobial card of their choice from another team at a later turn
       2. Infectious Disease Consult: This card is a wild card that will treat any pathogen card, and can be used at a later turn
       3. National Shortage: The team must give up an Antimicrobial card (of their choice)
       4. MDR: The team loses 1 point
       5. CLABSI: The team loses 5 points
       6. C diff: The team must spend each turn trying to treat C diff with a fidaxomicin or PO vancomycin card. If they have either card already, they can use it on a subsequent turn. If not, they must spend each turn either drawing an antimicrobial card to earn a fidaxomicin or PO vancomycin card (if they draw and alternative antimicrobial they can keep it if answering the question correctly) or buying a vancomycin card (see “buying” below)
       7. Vaccine cards: No matter how many Pathogen cards that team has at the end of their game, no points will be deducted
  1. Draw a Pathogen card from the top of the deck
     1. Teams have no limit on how many Pathogen cards they end up with. However, untreated Pathogen cards at the end of the game result in their corresponding points being deducted (except for if a team has the vaccine card).
  2. Buy an Antimicrobial
     1. Select Antimicrobials are for sale based on the list on the PowerPoint. The cost is points that the teams have earned from treating Pathogens. Teams cannot go into negative points by buying Antimicrobials.
  3. Treat a Pathogen card
     1. Teams can attempt to treat a Pathogen card by using a single or multiple Antimicrobial cards (depending on the Pathogen)
        1. The instructor has a master key on which Antimicrobial cards (by a letter code) treat the respective Pathogen Cards
        2. If treated correctly, the team earns the designated number of points on the Pathogen card
        3. If treated incorrectly, the team can keep both the Pathogen card and the Antimicrobial card, but they do not earn any points


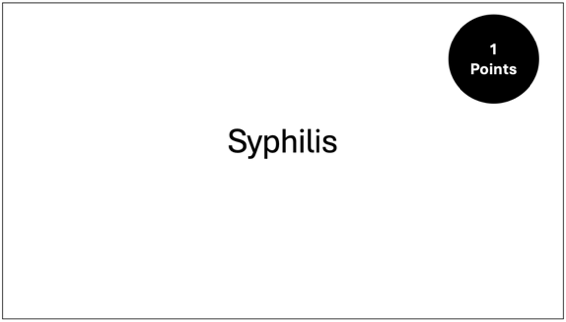


- - - 1. For example, using the Syphilis “Pathogen” card shown – if the team uses the Penicillin “Antimicrobial” card to treat, they then earn 1 point as indicated in the top right corner of the Pathogen card.
    1. There is no specified penalty for covering too broadly, though doing so may “waste” a broad-spectrum antibiotic when needed later in the game.
    2. Once Pathogen cards have been used, the instructor will place them in a discard pile to then be shuffled and reintroduced into the game when the deck runs out
  1. Use a steal card
     1. This card allows the team to steal an Antimicrobial card of their choice from another team at a later turn. Since cards are kept face up, this can allow teams to strategically steal a card to their own benefit, or to thwart another team from a future sucessful treatment.

Ending the game:

1. Depending on the preference of the instructor and participants, the game can end:
   1. Once all Antimicrobial cards have been drawn, and players are no longer able to treat further Pathogen Cards
   2. After a designated amount of time
   3. After a designated amount of rounds
2. Declaring a winner
   1. Points are added up throughout the game as teams treat Pathogen Cards
   2. At the conclusion of the game, teams add up the untreated Pathogen Cards that they still have in hand and subtract that from their score (the exception to this is if a team has a vaccine card, then they are not subtracted any points)

Modifications:

Consider the following modifications to alter the length of time or level of difficulty:

1. Face up version – To save time, rather than participants drawing Antimicrobial cards from a deck, instead lay them face up in a grid format so that participants can chose directly based on the antimicrobial they need to earn to treat one of the Pathogens they have in their hand. With this version, you must remove the Antimicrobial cards that are not associated with a question (e.g., the Infectious Disease Consult, Steal, PO vanc).
2. Open book version – if playing with medical students or early PGY-1 learners (e.g., within their first 6-months), consider allowing participants to use sources to answer questions and to properly treat pathogens. We strongly encourage against the use of artificial intelligence in this case.
3. Simplified version – to both save time and simplify the game, remove the viral Pathogen cards and antiviral Antimicrobial cards. The rest of the game can be played the same, or you can also make an additional modification such as the “Face up version” or “Open book version.”

**Instructions for the instructor:**

- - Print and cut out the Pathogen and Antimicrobial card decks on two different color papers. Keep the two sets of cards separate from eachother. Shuffle the two individual sets of cards within their deck.
  - Download and familiarize yourself with the electronic game-board (appendix E)
  - Split the participants into 3-5 groups, ideally consisting of 3-5 participants
  - Hand out a copy of the instructions to each group
  - Give each group 3 Pathogen cards
  - Decide which team will go first, and then proceed with the game in a counterclockwise fashion by team
