# Supplementary material for: Pathogen Pursuit: A Gamified Format to Learn Infectious Diseases and Antimicrobial Stewardship for Medical Residents
Source: MedEdPORTAL. 2025 Dec 16;21:11565. doi: 10.15766/mep_2374-8265.11565 (PMC12705857; doi:10.15766/mep_2374-8265.11565)
Supplement: Supplementary file 1 — Educational Objectives by Quesitons.docxGame Instructions.docxPathogen Game Cards.pdfAntimicrobial Game Cards.pdfGame Board Slide Show.pptxKey.pdfPostgame Survey.docxPre- and Posttest.docx [file mep_2374-8265.11565-s001.zip › C. Pathogen Game Cards.pdf]

Pathogen Cards

4 Points

Aspiration pneumonia

With an abscess

4 Points

Candida auris

1 Points

Community Acquired Pneumonia

Outpatient, no comorbidities

1 Points

Community Acquired Pneumonia

Outpatient, no comorbidities

1 Point

Chlamydia

1 Point

Chlamydia

2
Points

Community Acquired
Pneumonia

Inpatient

2
Points

Community Acquired
Pneumonia

Inpatient

2
Points

Enterobacter cloacae

2
Points

Enterobacter cloacae

1
Points

Enterococcus faecalis

1
Points

Enterococcus faecalis

4
Points

Enterococcus faecium

Pan resistant: resistant to ampicillin, vancomycin, daptomycin, linezolid

3
Points

ESBL *E. coli* Pyelonephritis

3
Points

ESBL *E. coli* Pyelonephritis

4
Points

Esophageal Candidiasis

1
Points

Genital herpes flare

1
Points

Genital herpes flare

1
Points

Gonorrhea

1
Points

Gonorrhea

5
Points

Hepatitis C

2
Points

Herpes-zoster

Disseminated

3
Points

HIV

3
Points

HIV

4 Points

Invasive aspergillus

2 Points

Lyme disease

6 Points

Malaria

4 Points

Meningitis

In a 23-year-old

5 Points

Meningitis

In a 59-year-old

2 Points

MRSA Bacteremia

2
Points

MRSA Bacteremia

2
Points

MRSA Bacteremia

2
Points

MRSA Bacteremia

2
Points

MRSA Osteomyelitis

2
Points

MRSA Osteomyelitis

2
Points

MRSA Pneumonia

2 Points

MRSA Pneumonia

4 Points

Mucormycosis

5 Points

Naegleria fowleri

4 Points

Neuroborreliosis

3 Points

Neurosyphilis

2 Points

Non-purulent cellulitis

2
Points

Non-purulent cellulitis

2
Points

Pneumocystis jerovicii

2
Points

Pneumocystis jerovicii

4
Points

Post-surgical meningitis

3
Points

Pseudomonas

Resistant to piperacillin-tazobactam and cefepime

3
Points

Pseudomonas

Resistant to piperacillin-tazobactam and cefepime

6
Points

Pseudomonas

Resistant to piperacillin-tazobactam, cefepime, and carbapenems

2
Points

Pseudomonas pneumonia

2
Points

Pseudomonas pneumonia

2
Points

Purulent cellulitis

2
Points

Purulent cellulitis

2
Points

Purulent cellulitis

2
Points

Purulent cellulitis

2
Points

Rocky mountain spotted fever

1
Points

Simple cystitis

1
Points

Simple cystitis

1
Points

Simple cystitis

1
Points

Simple cystitis

2 Points

Stenotrophomonas

1 Points

Strep pharyngitis

1 Points

Strep pharyngitis

1 Points

Syphilis

1 Points

Syphilis

3 Points

Tinea corporis
