# Supplementary material for: Pathogen Pursuit: A Gamified Format to Learn Infectious Diseases and Antimicrobial Stewardship for Medical Residents
Source: MedEdPORTAL. 2025 Dec 16;21:11565. doi: 10.15766/mep_2374-8265.11565 (PMC12705857; doi:10.15766/mep_2374-8265.11565)
Supplement: Supplementary file 1 — Educational Objectives by Quesitons.docxGame Instructions.docxPathogen Game Cards.pdfAntimicrobial Game Cards.pdfGame Board Slide Show.pptxKey.pdfPostgame Survey.docxPre- and Posttest.docx [file mep_2374-8265.11565-s001.zip › D. Antimicrobial Game Cards.pdf]

1

Amoxicillin or Ampicillin

2

Penicillin

3

Doxycycline

4

Cefazolin

5

Ceftriaxone

6

Cefepime

7

Clindamycin

8

Ceftazidime-Avibactam

9

Piperacillin-tazobactam

10

Meropenem

11

Ertapenem

12

Ceftriaxone

13

Piperacillin-tazobactam

14

Doxycycline

15

Trimethoprim-
sulfamethoxazole

16

Emtricitabine-tenofovir

17

Bictegravir

18

Dolutegravir

19

Lamivudine

20

Emtricitabine-tenofovir

21

Bictegravir

22

Acyclovir

23

Valcyclovir

24

Fluconazole

25

Clotrimazole

26

Voriconazole

27

Micafungin

28

Amphotericin B

29

Ciprofloxacin

30

Metronidazole

31

IV Vancomycin

32

Trimethoprim-
sulfamethazole

33

Daptomycin

34

Linezolid

35

Nitrofurantoin

36

Clindamycin

37

Azithromycin

38

Ampicillin-sulbactam

39

IV Vancomycin

40

Acyclovir

41

Ceftriaxone

42

Valcyclovir

43

Tigecycline

44

Artesunate

45

Ivermectin

46

Ivermectin

47

Trimethoprim-
sulfamethoxazole

48

Doxycycline

49

Azithromycin

50

Sofosbuvir-velpatasvir

Steal

Choose an antimicrobial from another team

Steal

Choose an antimicrobial from another team

Infectious disease consult

Wild card that can be used against any pathogen or disease

National shortage

Lose one of your antimicrobials

New MDR organism

You covered too broadly! Loose 1 point

CLABSI

Lose 5 points

Infectious disease consult

Wild card that can be used against any pathogen or disease

Vaccine

No points are deducted from you at the end of the game

C. diff infection

Uh oh! You need to treat *C diff* before you can treat another pathogen

C. diff infection

Uh oh! You need to treat *C diff* before you can treat another pathogen

C. diff infection

Uh oh! You need to treat *C diff* before you can treat another pathogen

Fidaxomicin

Fidaxomicin

Fidaxomicin

Fidaxomicin

PO Vancomycin

PO Vancomycin

PO Vancomycin
