# Supplementary material for: Pathogen Pursuit: A Gamified Format to Learn Infectious Diseases and Antimicrobial Stewardship for Medical Residents
Source: MedEdPORTAL. 2025 Dec 16;21:11565. doi: 10.15766/mep_2374-8265.11565 (PMC12705857; doi:10.15766/mep_2374-8265.11565)
Supplement: Supplementary file 1 — Educational Objectives by Quesitons.docxGame Instructions.docxPathogen Game Cards.pdfAntimicrobial Game Cards.pdfGame Board Slide Show.pptxKey.pdfPostgame Survey.docxPre- and Posttest.docx [file mep_2374-8265.11565-s001.zip › E. Game Board Slide Show.pptx]

## Slide 1
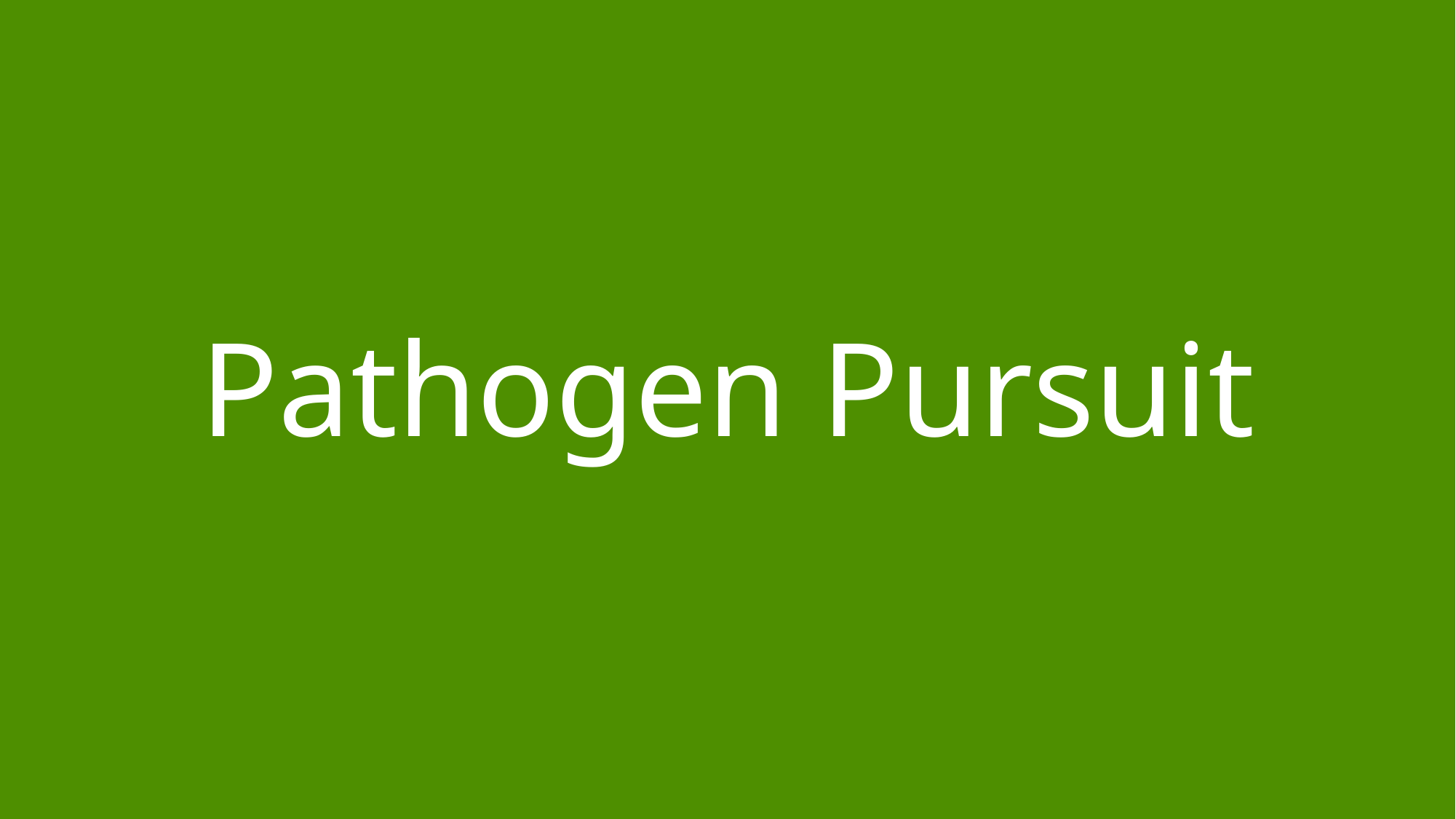

# Pathogen Pursuit

## Slide 2
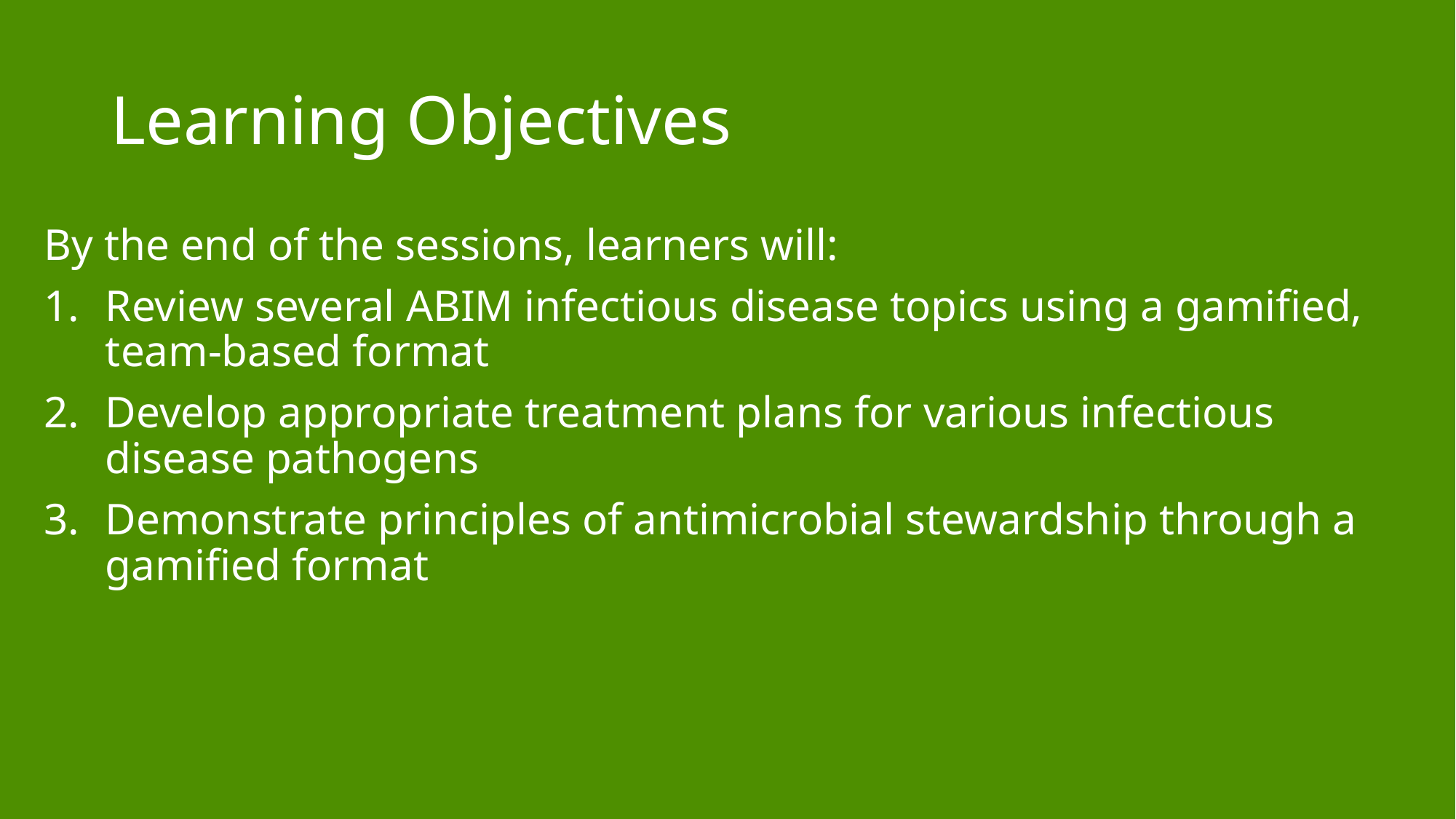

# Learning Objectives
By the end of the sessions, learners will:
Review several ABIM infectious disease topics using a gamified, team-based format
Develop appropriate treatment plans for various infectious disease pathogens
Demonstrate principles of antimicrobial stewardship through a gamified format

## Slide 3
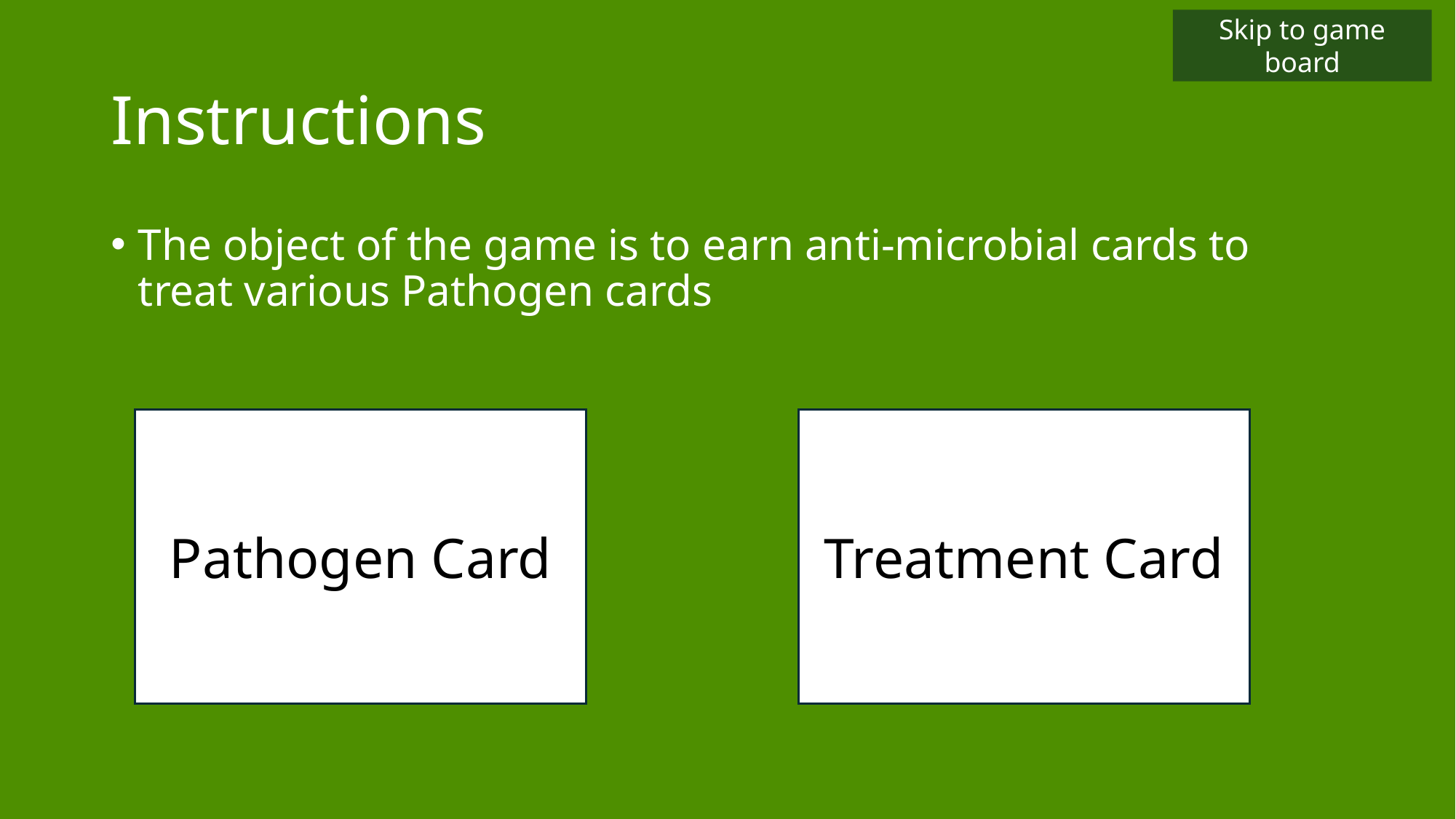

Skip to game board
# Instructions
The object of the game is to earn anti-microbial cards to treat various Pathogen cards
Treatment Card
Pathogen Card

## Slide 4
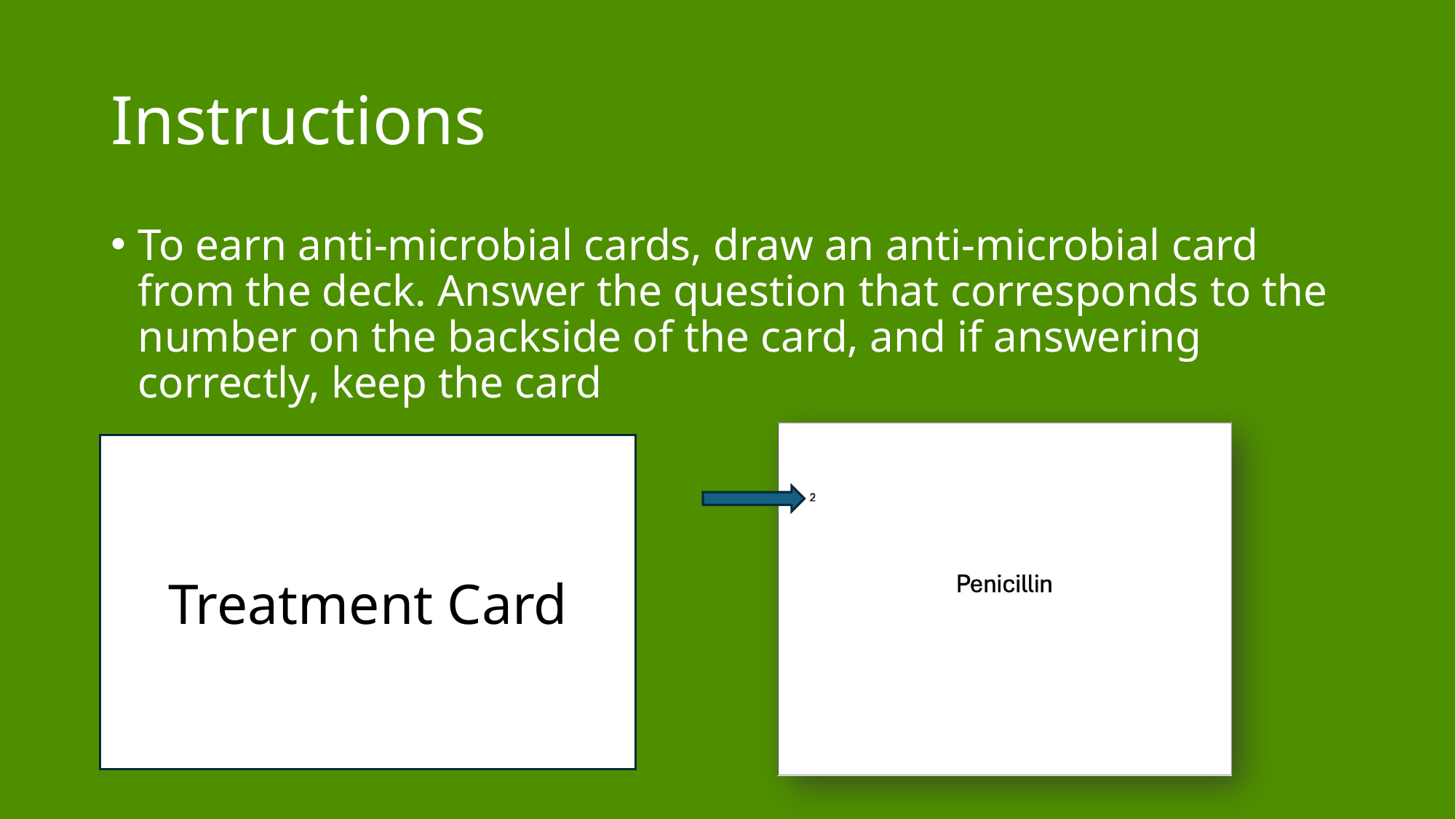

# Instructions
To earn anti-microbial cards, draw an anti-microbial card from the deck. Answer the question that corresponds to the number on the backside of the card, and if answering correctly, keep the card
Treatment Card

## Slide 5
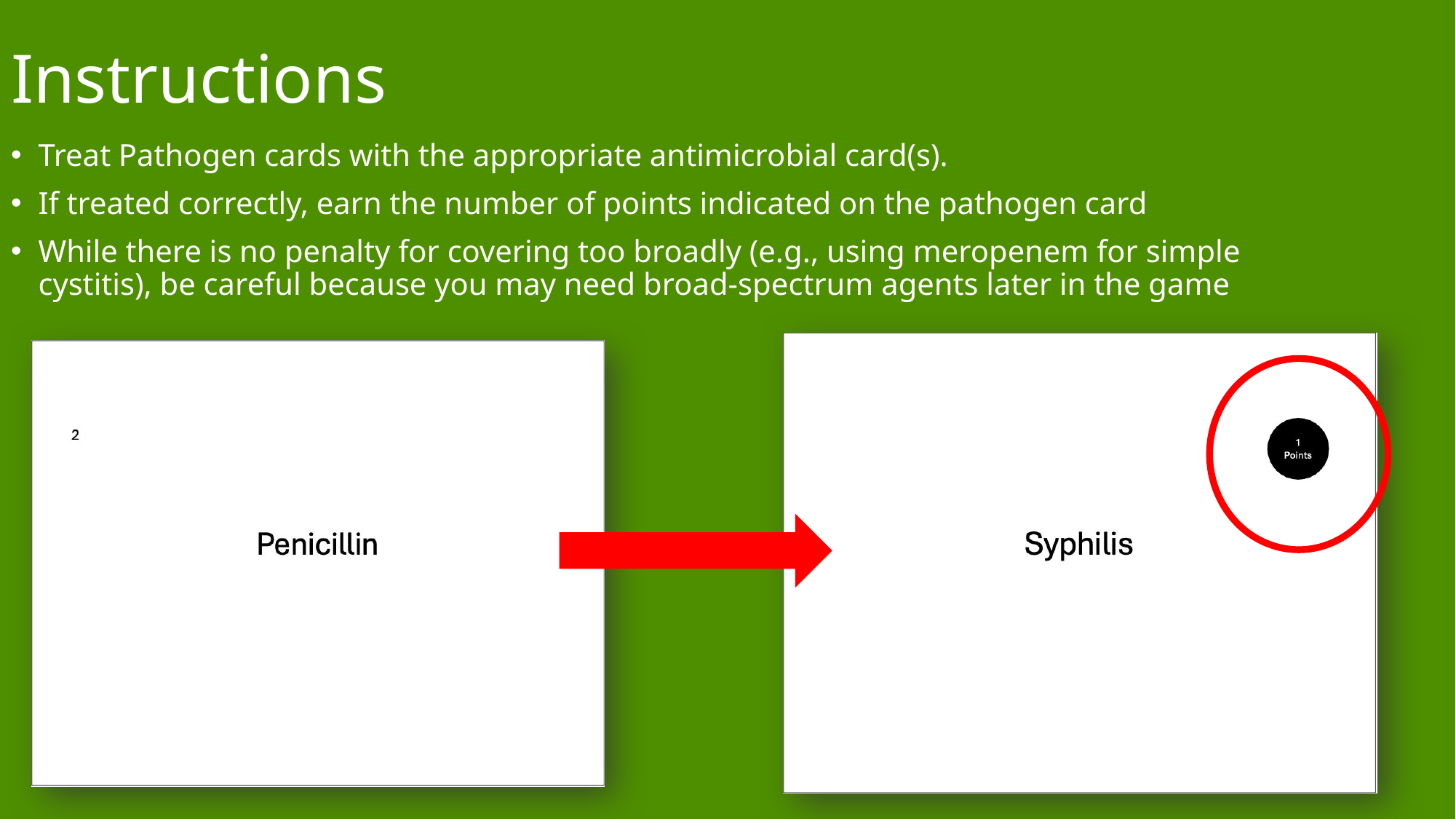

# Instructions
Treat Pathogen cards with the appropriate antimicrobial card(s).
If treated correctly, earn the number of points indicated on the pathogen card
While there is no penalty for covering too broadly (e.g., using meropenem for simple cystitis), be careful because you may need broad-spectrum agents later in the game

## Slide 6
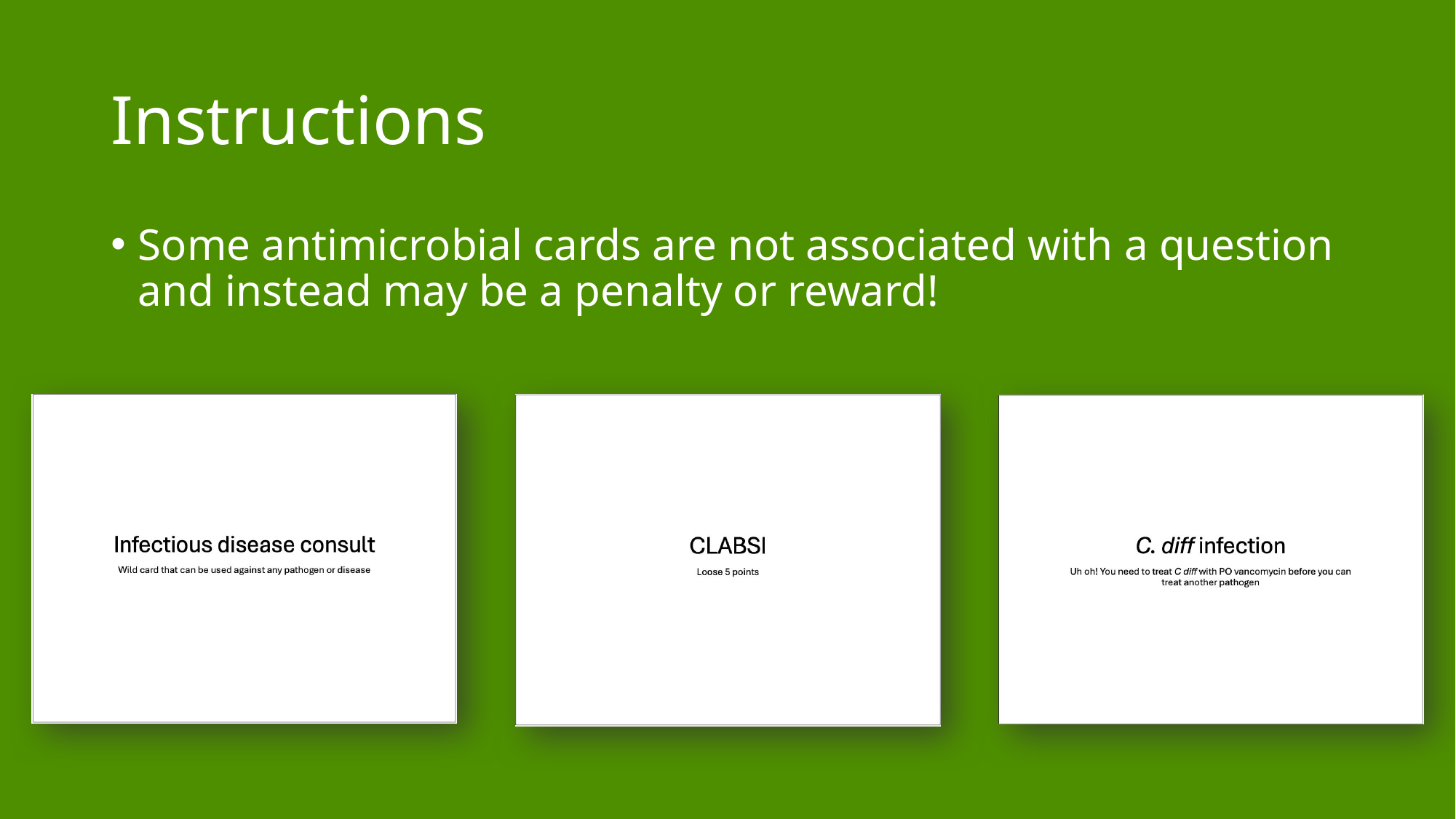

# Instructions
Some antimicrobial cards are not associated with a question and instead may be a penalty or reward!

## Slide 7
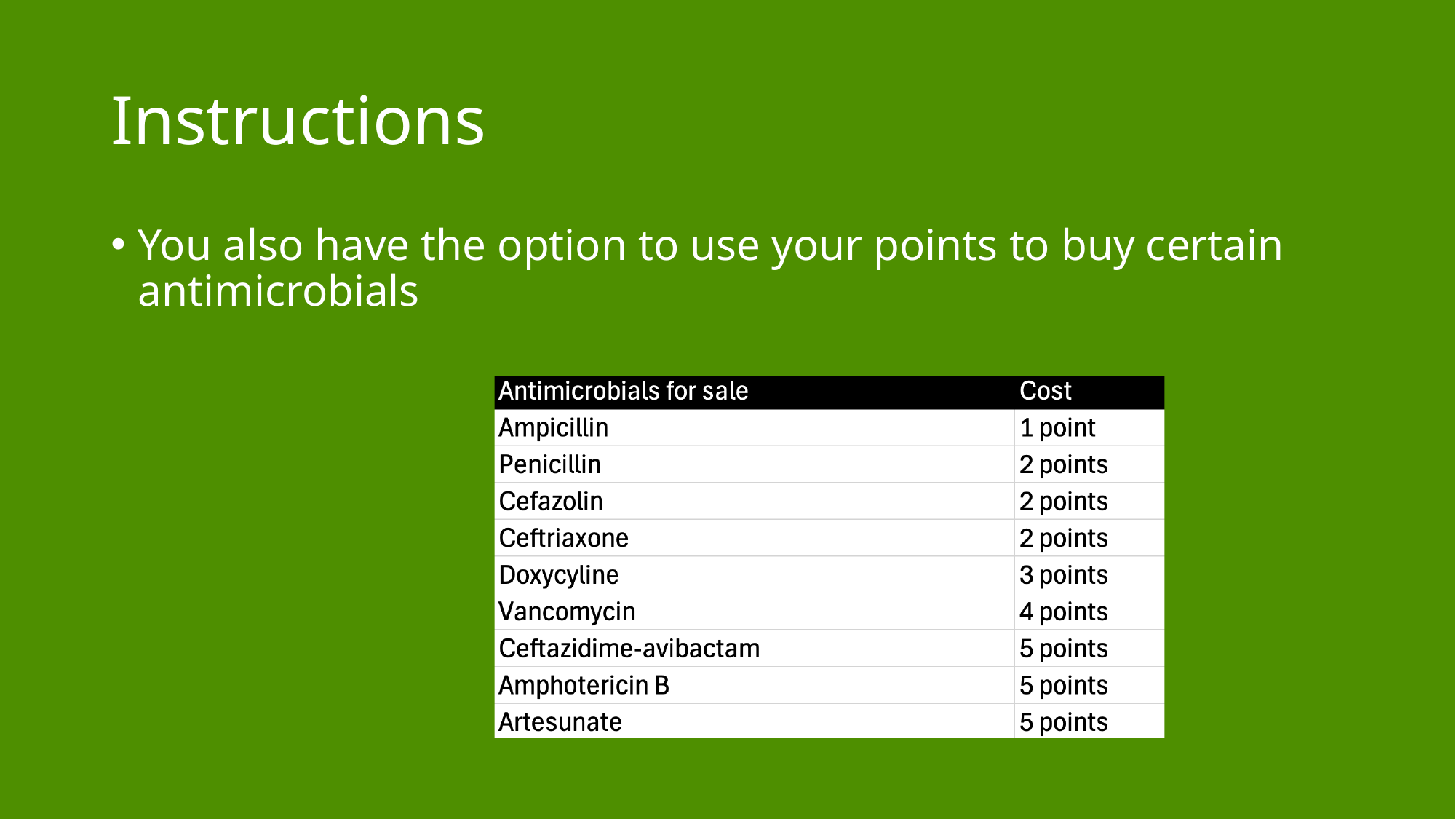

# Instructions
You also have the option to use your points to buy certain antimicrobials

## Slide 8
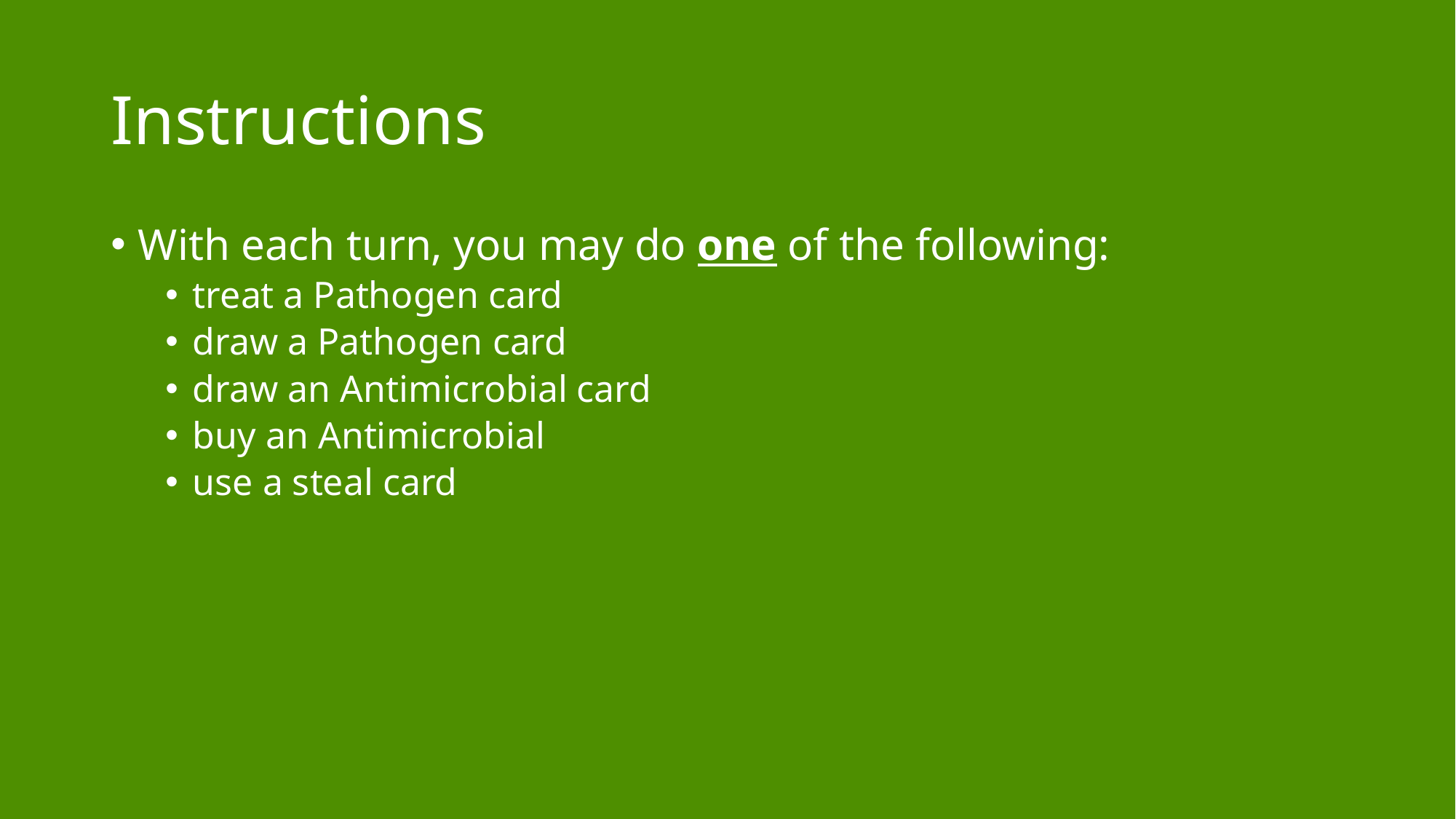

# Instructions
With each turn, you may do one of the following:
treat a Pathogen card
draw a Pathogen card
draw an Antimicrobial card
buy an Antimicrobial
use a steal card

## Slide 9
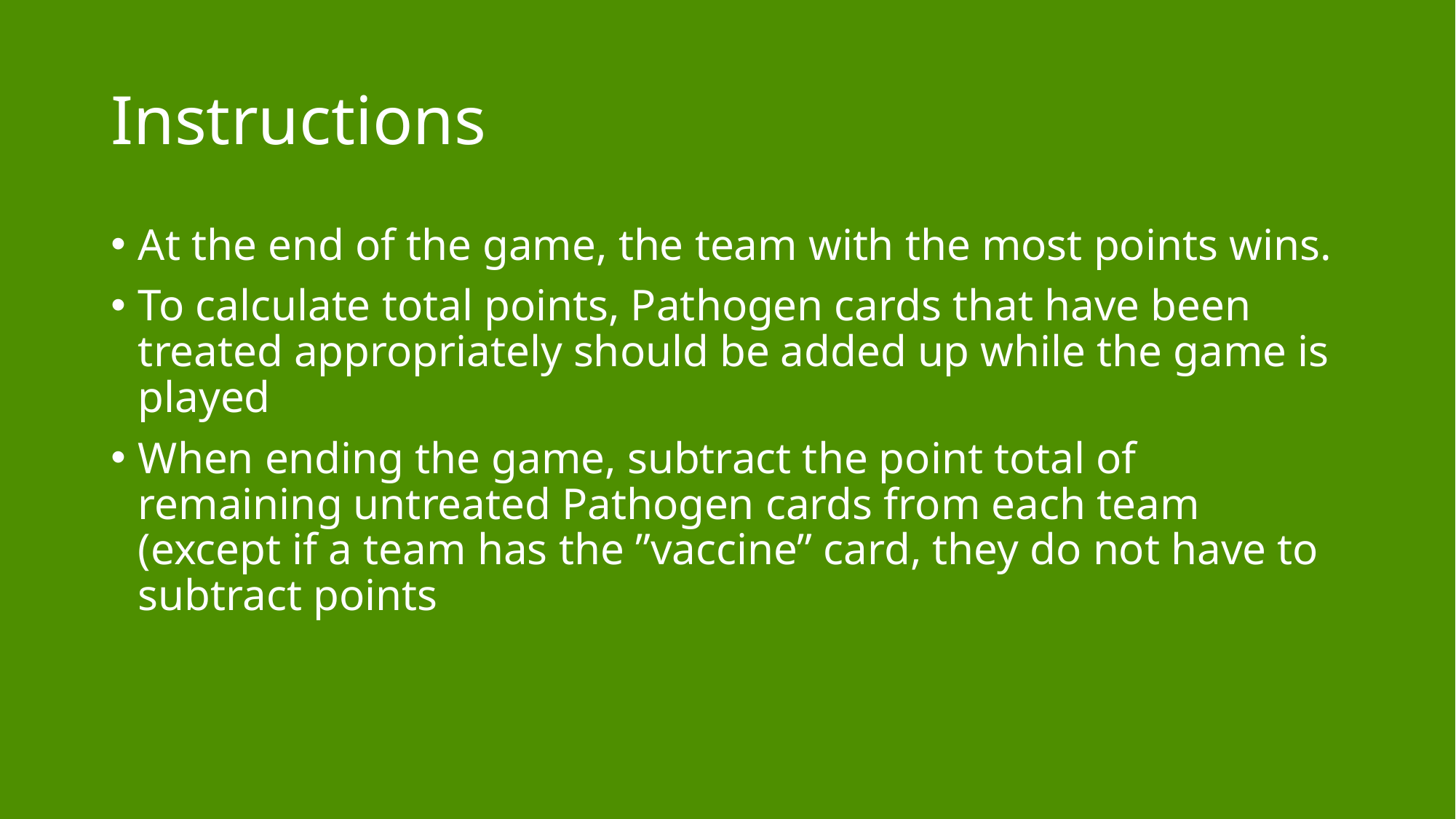

# Instructions
At the end of the game, the team with the most points wins.
To calculate total points, Pathogen cards that have been treated appropriately should be added up while the game is played
When ending the game, subtract the point total of remaining untreated Pathogen cards from each team (except if a team has the ”vaccine” card, they do not have to subtract points

## Slide 10
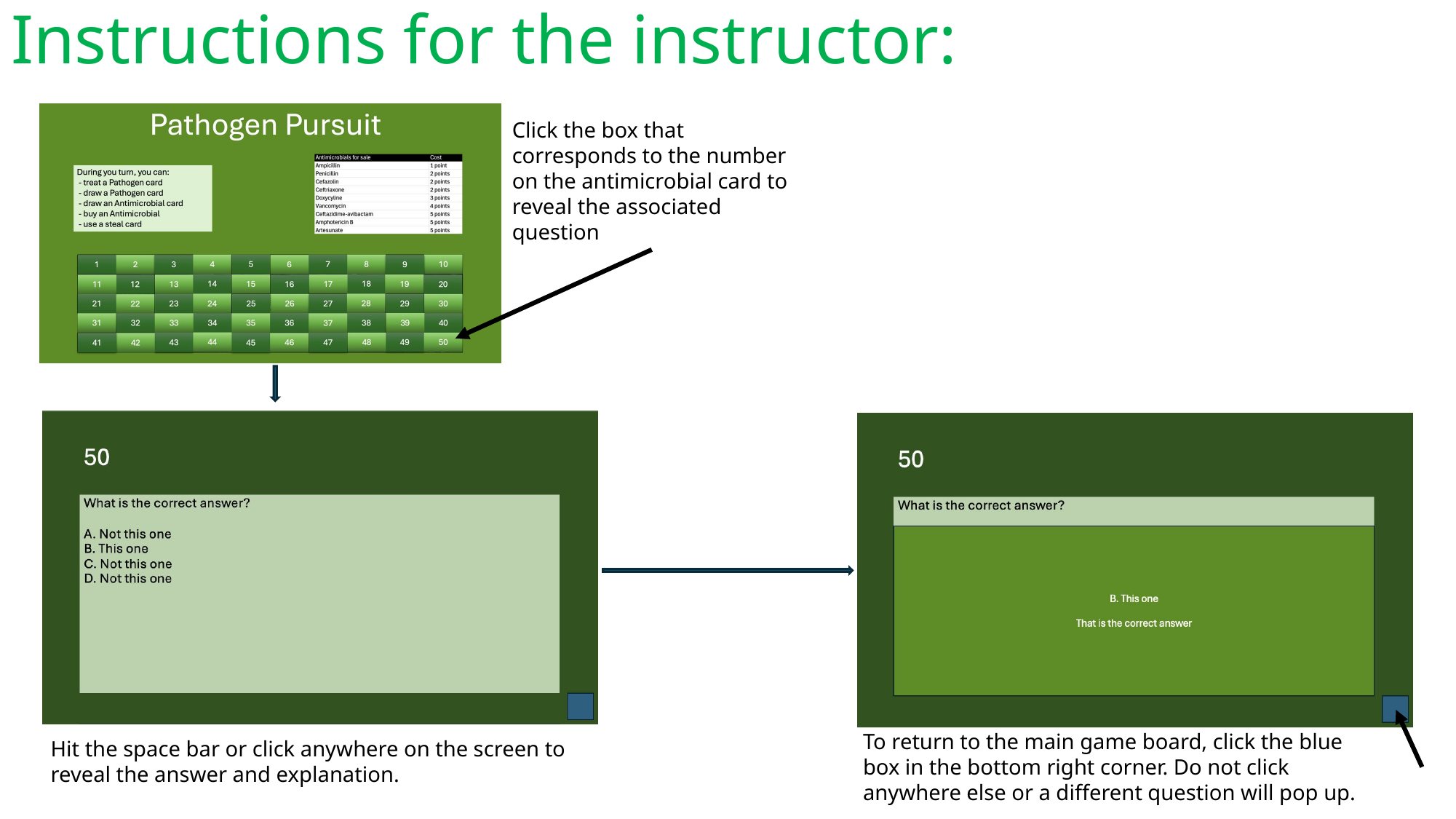

# Instructions for the instructor:
Click the box that corresponds to the number on the antimicrobial card to reveal the associated question
To return to the main game board, click the blue box in the bottom right corner. Do not click anywhere else or a different question will pop up.
Hit the space bar or click anywhere on the screen to reveal the answer and explanation.

## Slide 11
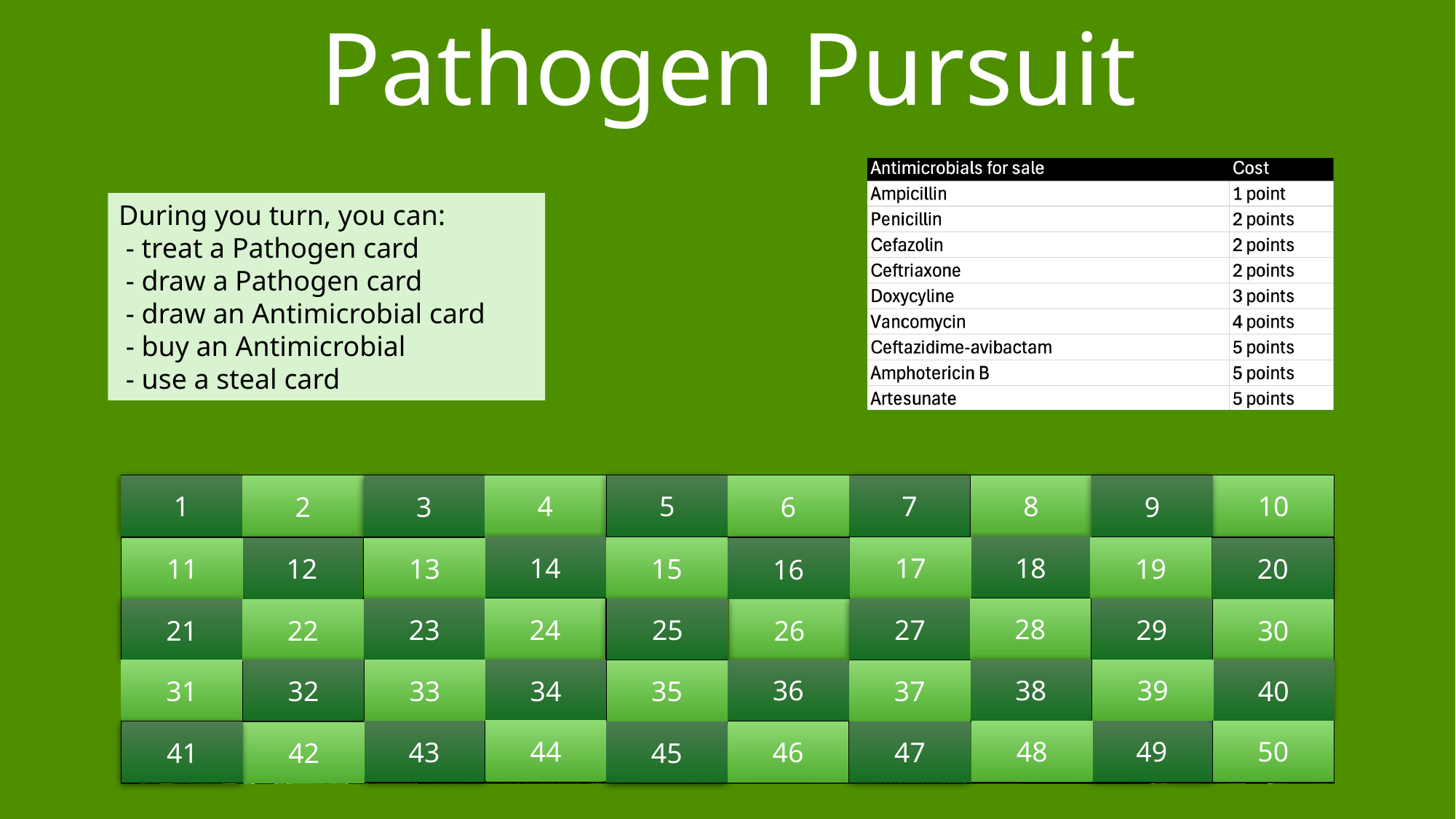

Pathogen Pursuit
During you turn, you can:
 - treat a Pathogen card
 - draw a Pathogen card
 - draw an Antimicrobial card
 - buy an Antimicrobial
 - use a steal card
4
5
7
8
10
1
| | | | | | | | | | |
| --- | --- | --- | --- | --- | --- | --- | --- | --- | --- |
| | | | | | | | | | |
| | | | | | | | | | |
| | | | | | | | | | |
| | | | | | | | | | |
2
3
6
9
14
18
17
19
15
11
12
13
20
16
28
24
23
25
27
29
21
26
30
22
36
39
38
40
31
33
32
34
35
37
44
48
49
50
43
46
47
41
45
42

## Slide 12
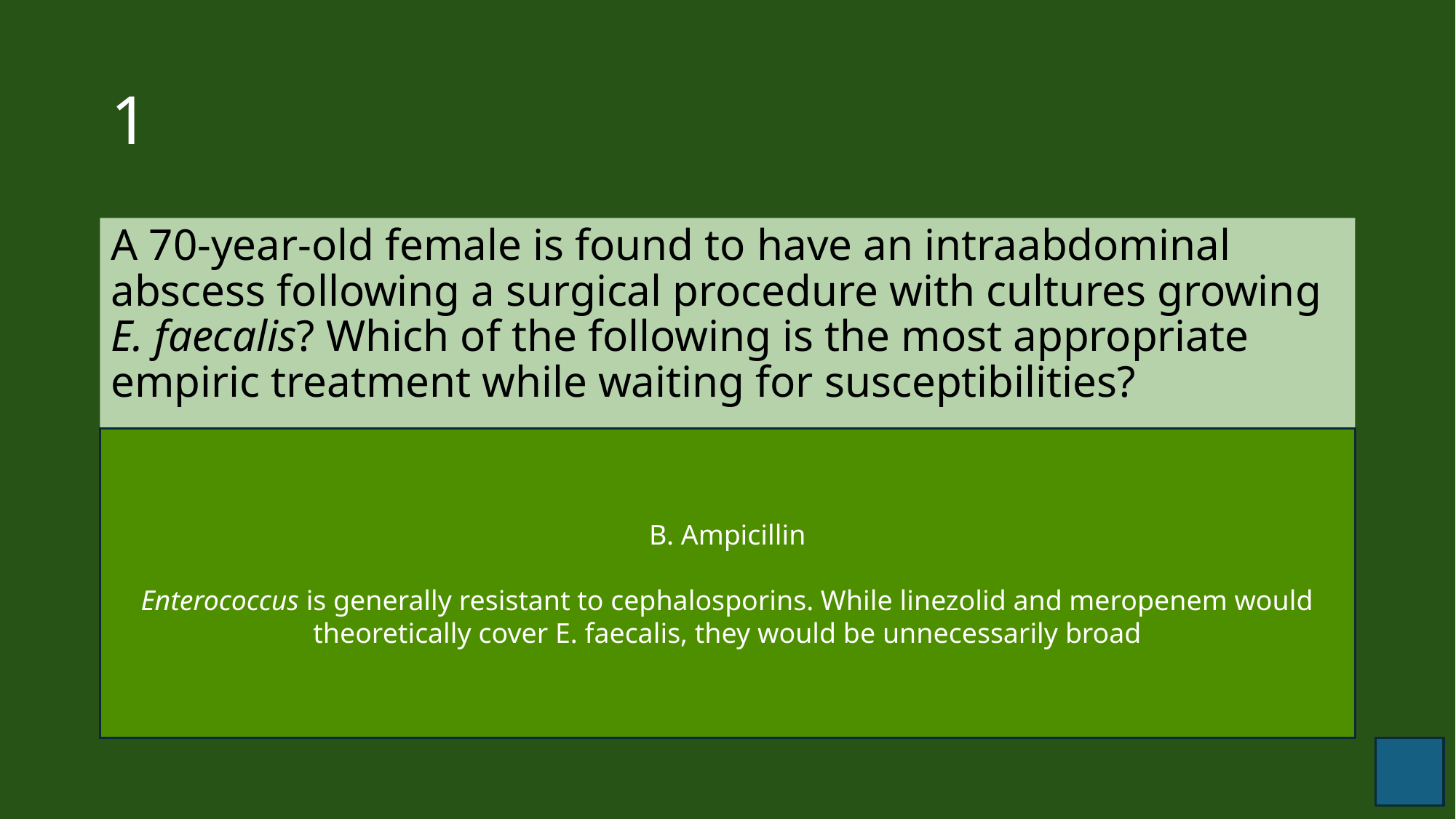

# 1
A 70-year-old female is found to have an intraabdominal abscess following a surgical procedure with cultures growing E. faecalis? Which of the following is the most appropriate empiric treatment while waiting for susceptibilities?
Cefazolin
Ampicillin
Linezolid
Meropenem
B. Ampicillin
Enterococcus is generally resistant to cephalosporins. While linezolid and meropenem would theoretically cover E. faecalis, they would be unnecessarily broad

## Slide 13
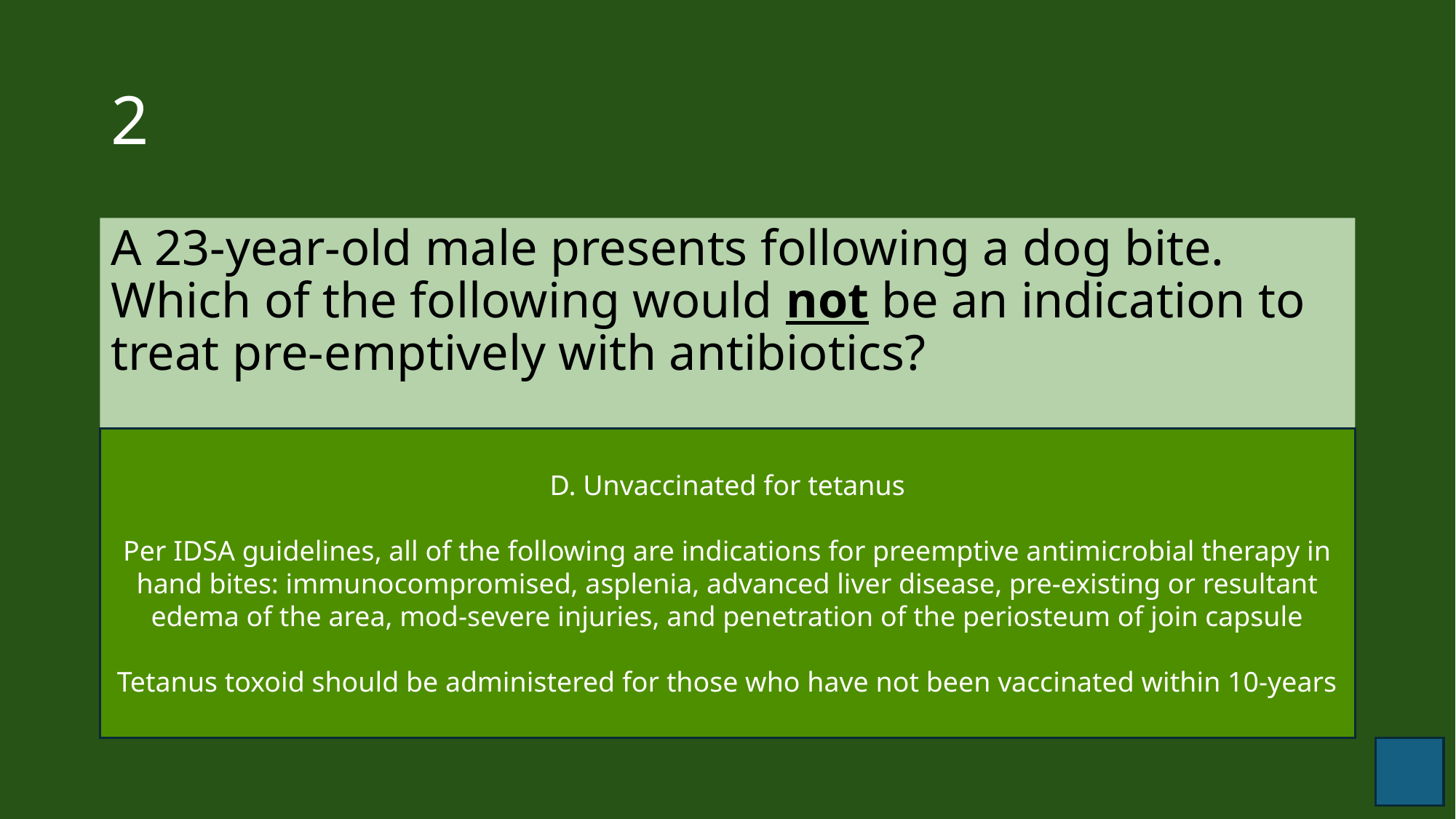

# 2
A 23-year-old male presents following a dog bite. Which of the following would not be an indication to treat pre-emptively with antibiotics?
 Edema
 Immunocompromised
 Involvement of the hands
 Unvaccinated for tetanus
D. Unvaccinated for tetanus
Per IDSA guidelines, all of the following are indications for preemptive antimicrobial therapy in hand bites: immunocompromised, asplenia, advanced liver disease, pre-existing or resultant edema of the area, mod-severe injuries, and penetration of the periosteum of join capsule
Tetanus toxoid should be administered for those who have not been vaccinated within 10-years

## Slide 14
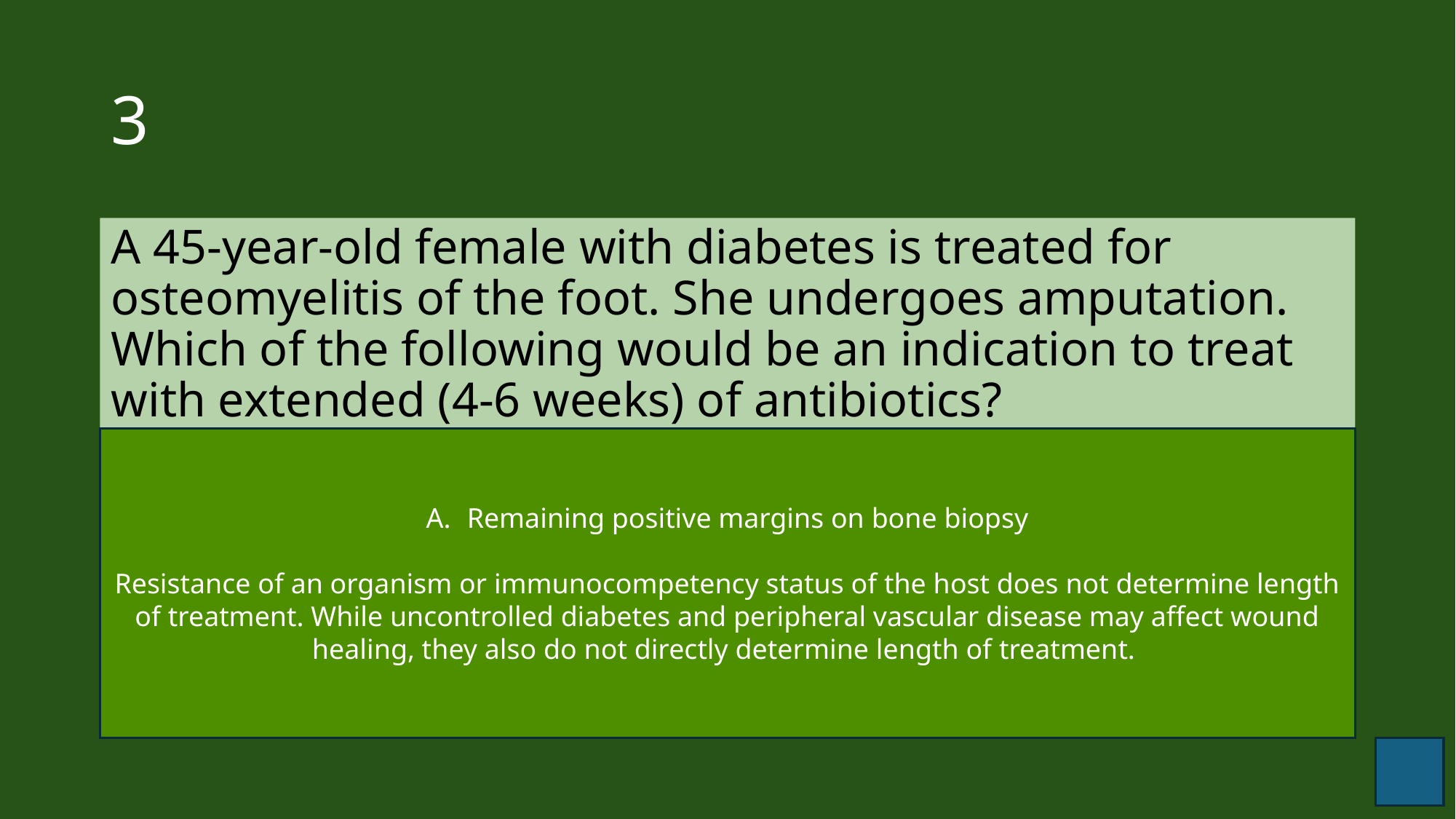

# 3
A 45-year-old female with diabetes is treated for osteomyelitis of the foot. She undergoes amputation. Which of the following would be an indication to treat with extended (4-6 weeks) of antibiotics?
 Remaining positive-margins on bone biopsy
 MDR organism
 Immunocompromised
 Underlying peripheral vascular disease
 A1c >10
Remaining positive margins on bone biopsy
Resistance of an organism or immunocompetency status of the host does not determine length of treatment. While uncontrolled diabetes and peripheral vascular disease may affect wound healing, they also do not directly determine length of treatment.

## Slide 15
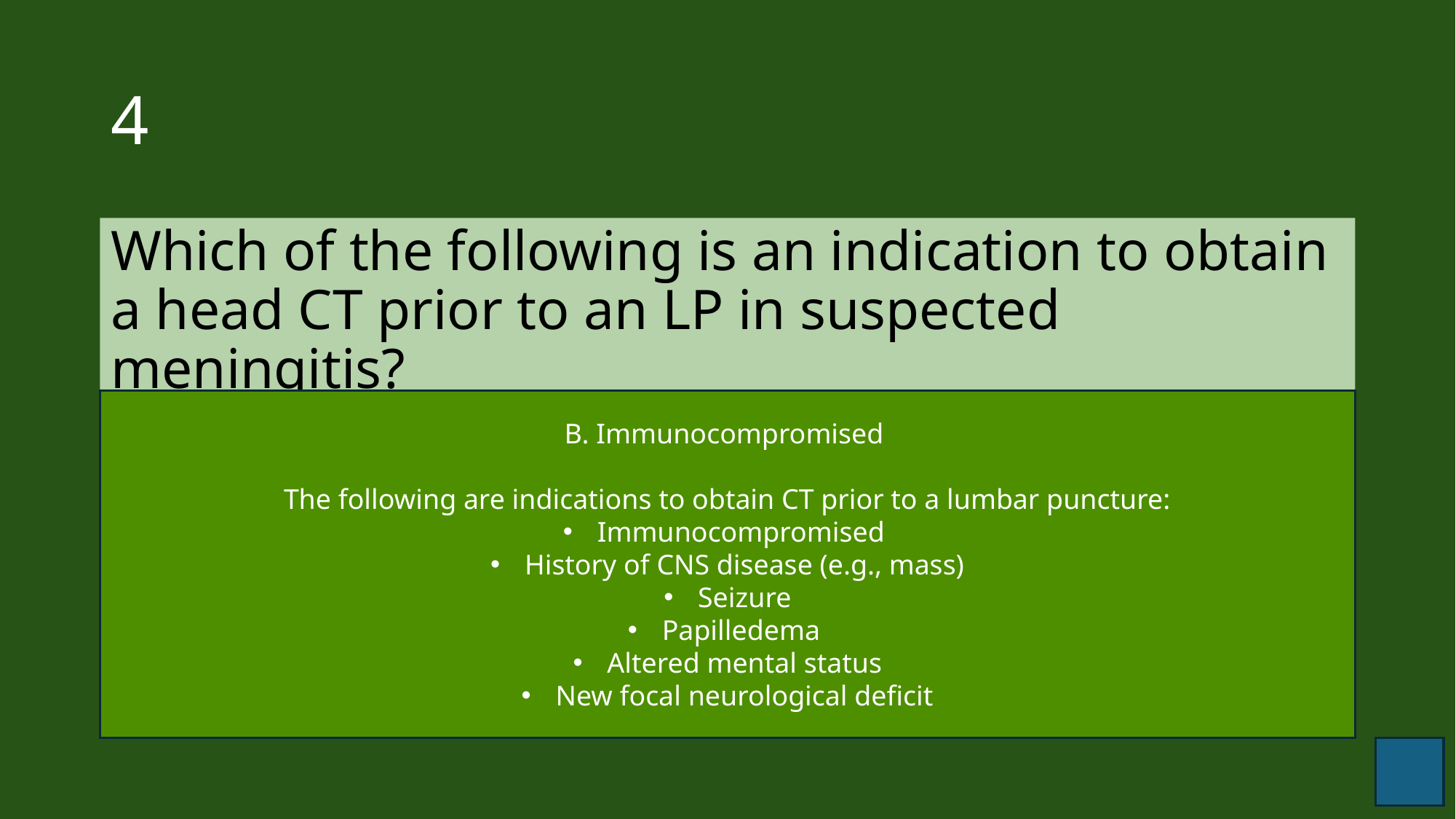

# 4
Which of the following is an indication to obtain a head CT prior to an LP in suspected meningitis?
 Headache
 Immunocompromised
 Photosensitivity
 Suspected viral etiology
B. Immunocompromised
The following are indications to obtain CT prior to a lumbar puncture:
Immunocompromised
History of CNS disease (e.g., mass)
Seizure
Papilledema
Altered mental status
New focal neurological deficit

## Slide 16
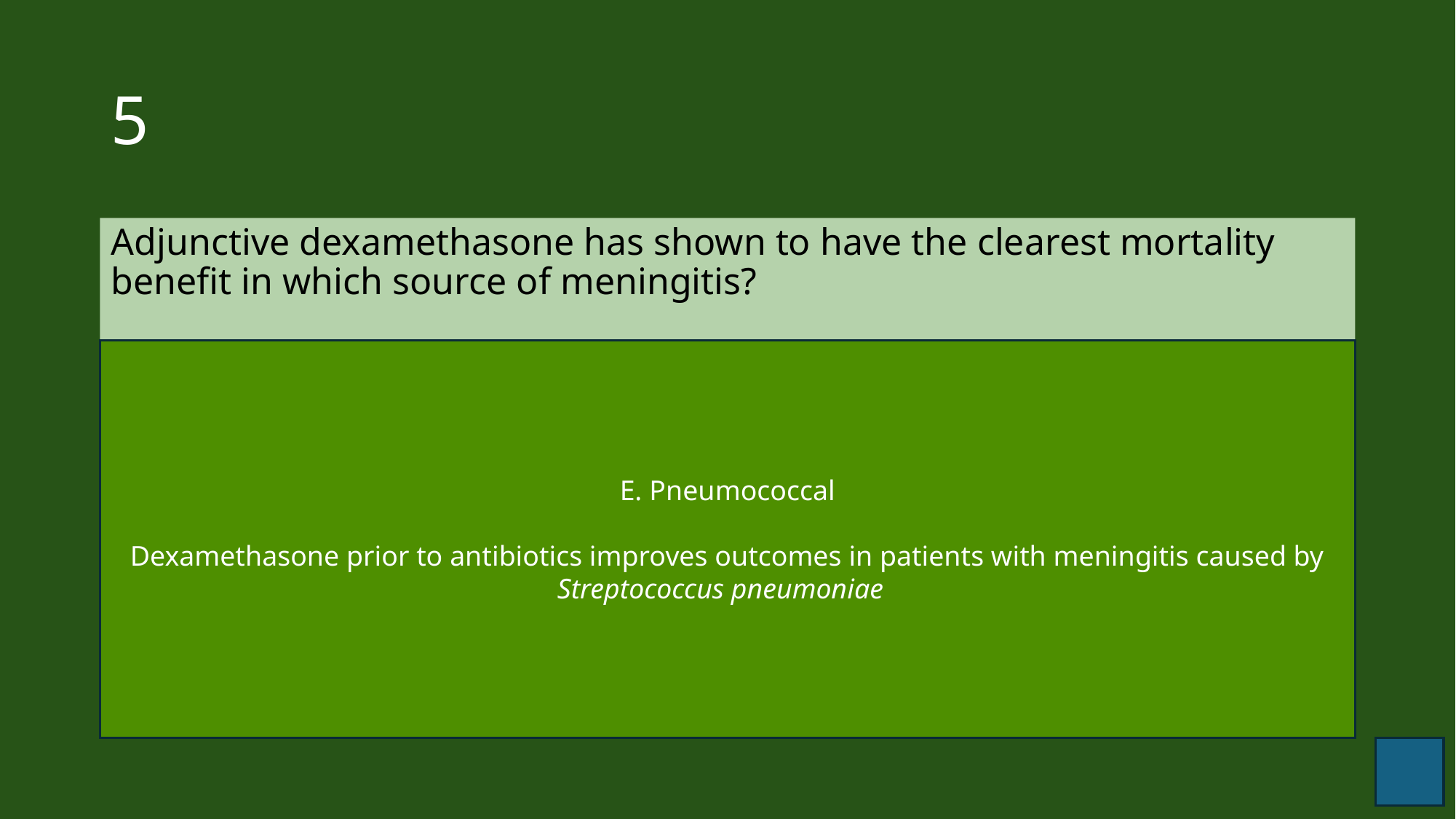

# 5
Adjunctive dexamethasone has shown to have the clearest mortality benefit in which source of meningitis?
Meningococcal
HSV-2
Cryptococcal
Listeria
Pneumococcal
E. Pneumococcal
Dexamethasone prior to antibiotics improves outcomes in patients with meningitis caused by Streptococcus pneumoniae

## Slide 17
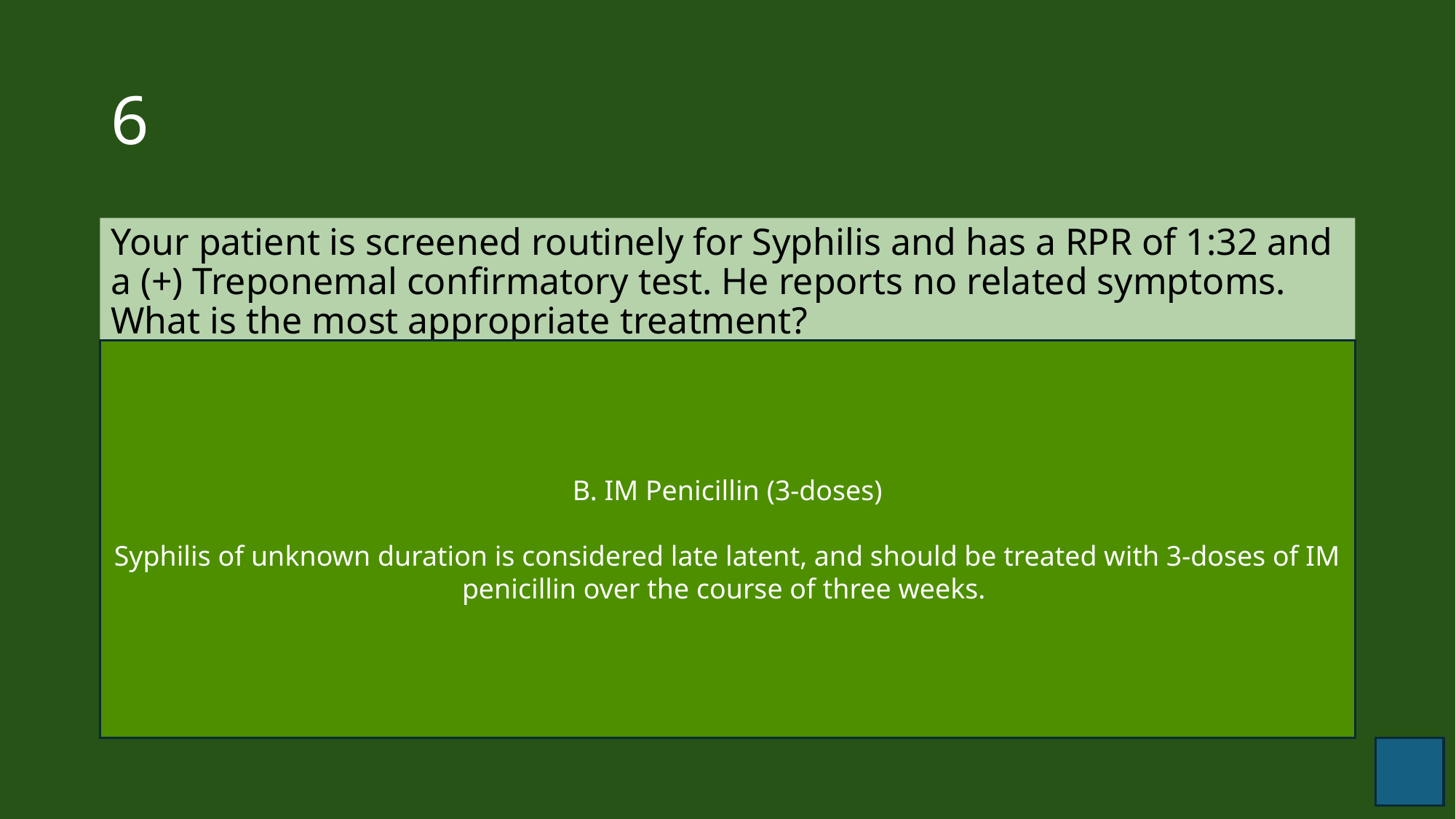

# 6
Your patient is screened routinely for Syphilis and has a RPR of 1:32 and a (+) Treponemal confirmatory test. He reports no related symptoms. What is the most appropriate treatment?
IM Penicillin (single dose)
IM Penicillin (three doses)
Oral doxycycline for 28-days
Clinical observation and sequential RPRs
B. IM Penicillin (3-doses)
Syphilis of unknown duration is considered late latent, and should be treated with 3-doses of IM penicillin over the course of three weeks.

## Slide 18
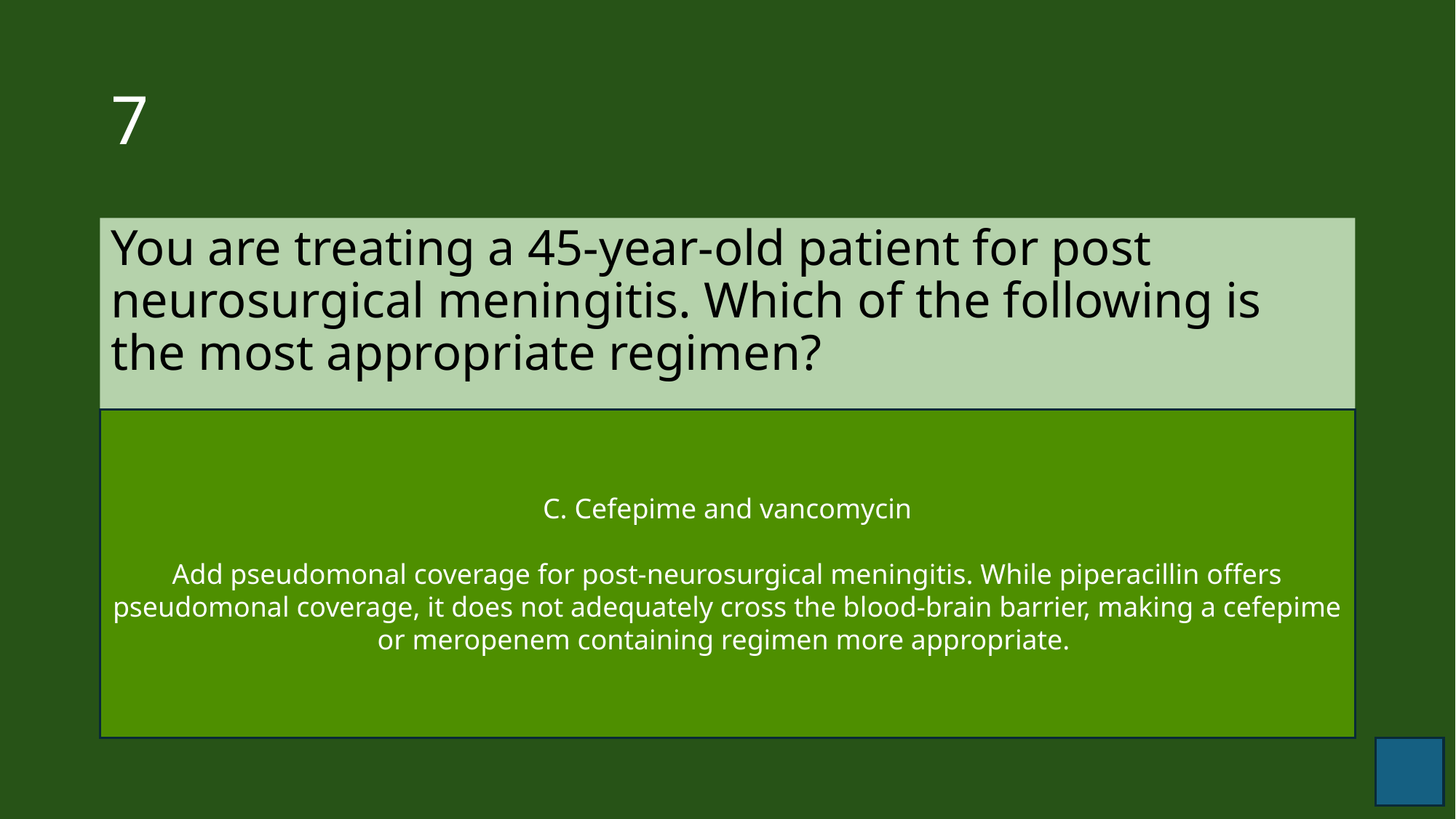

# 7
You are treating a 45-year-old patient for post neurosurgical meningitis. Which of the following is the most appropriate regimen?
Ceftriaxone and vancomycin
Piperacillin-tazobactam and vancomycin
Cefepime and vancomycin
Ceftriaxone, vancomycin, and ampicillin
C. Cefepime and vancomycin
Add pseudomonal coverage for post-neurosurgical meningitis. While piperacillin offers pseudomonal coverage, it does not adequately cross the blood-brain barrier, making a cefepime or meropenem containing regimen more appropriate.

## Slide 19
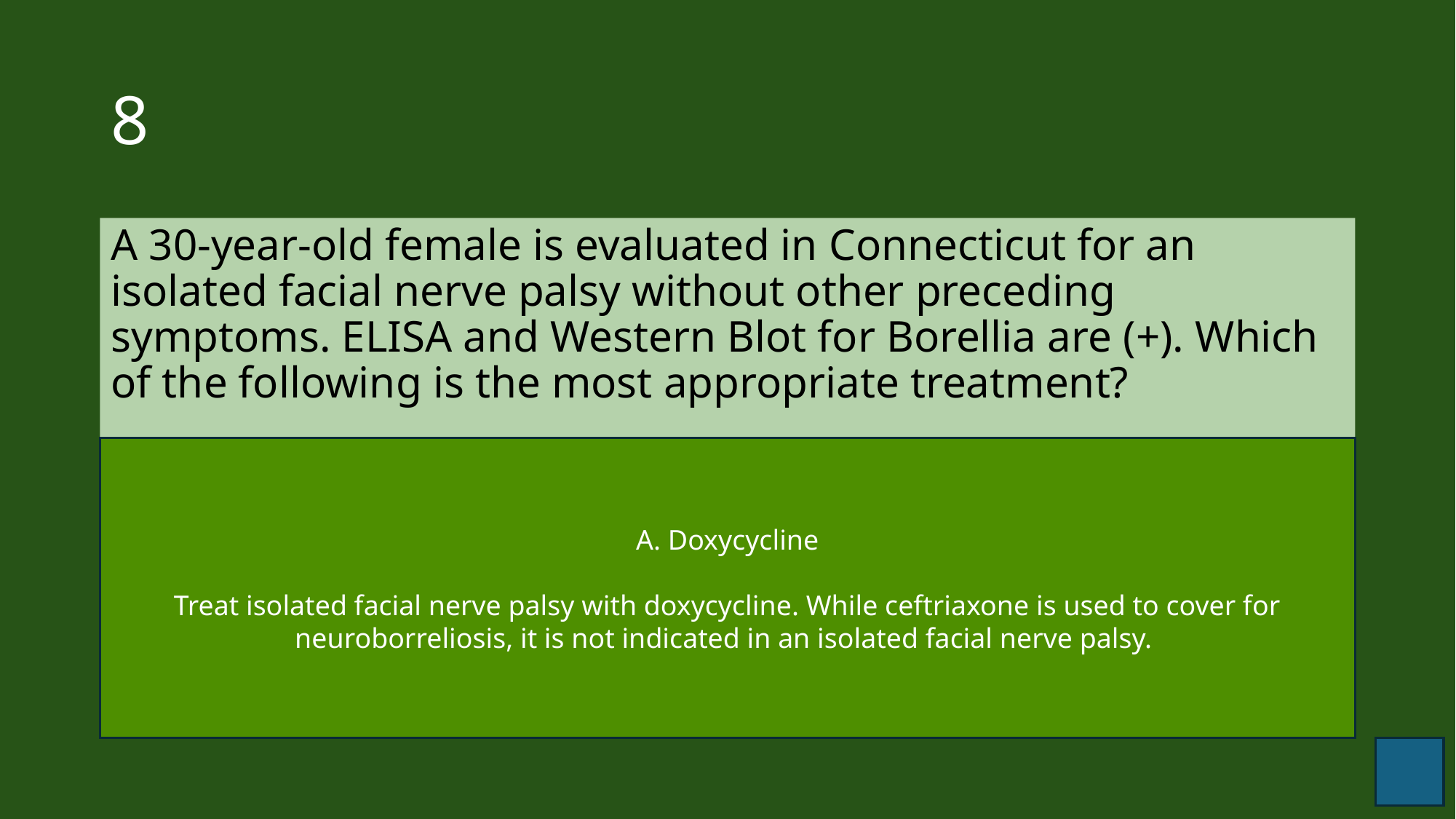

# 8
A 30-year-old female is evaluated in Connecticut for an isolated facial nerve palsy without other preceding symptoms. ELISA and Western Blot for Borellia are (+). Which of the following is the most appropriate treatment?
Doxycycline
Ceftriaxone
Azithromycin and atovaquone
Glucocorticoids alone
Doxycycline and glucocorticoids
A. Doxycycline
Treat isolated facial nerve palsy with doxycycline. While ceftriaxone is used to cover for neuroborreliosis, it is not indicated in an isolated facial nerve palsy.

## Slide 20
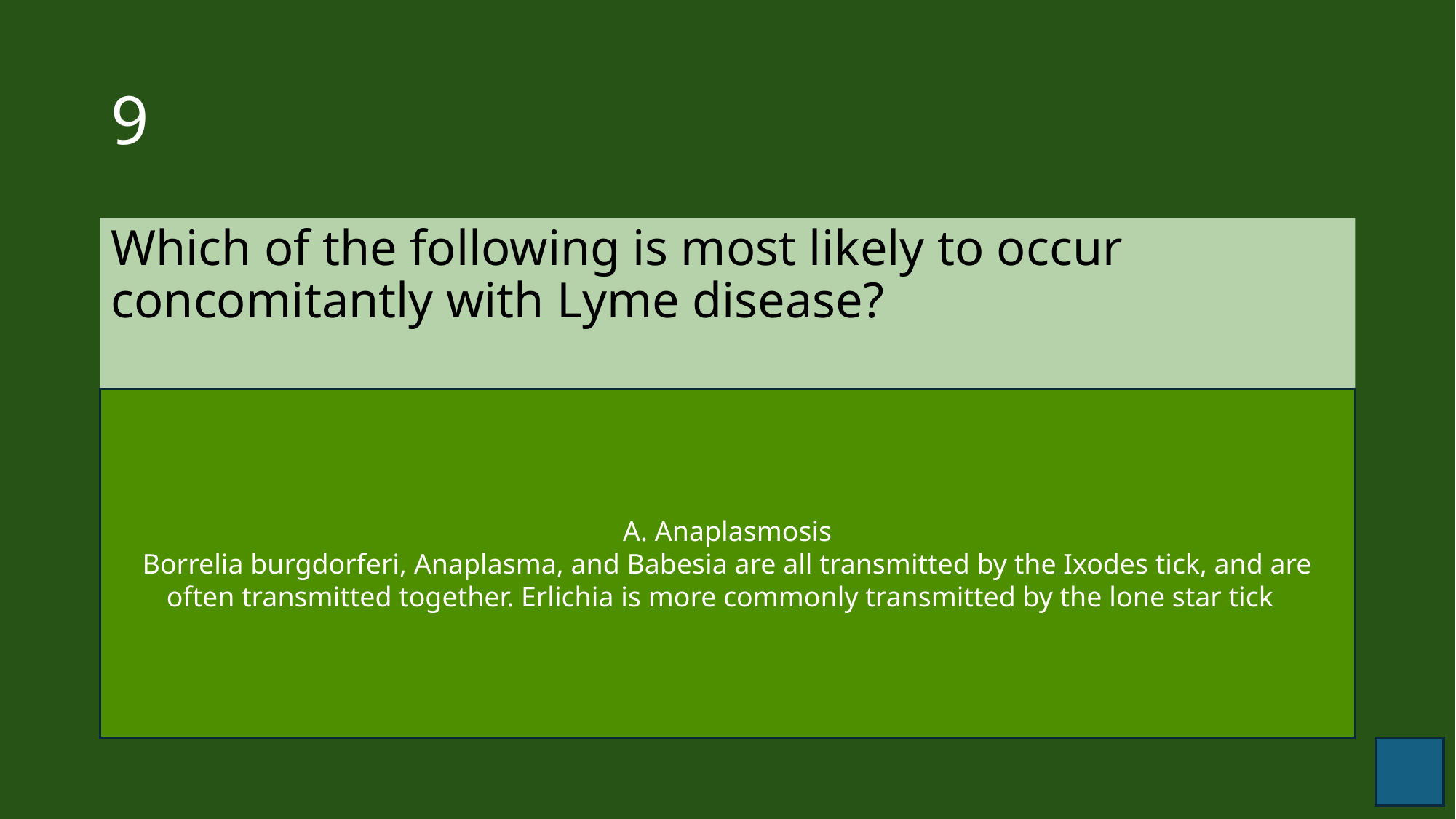

# 9
Which of the following is most likely to occur concomitantly with Lyme disease?
 Anaplasmosis
 Ehrlichiosis
 Rickettsia rickettsia
 West-Nile virus
A. Anaplasmosis
Borrelia burgdorferi, Anaplasma, and Babesia are all transmitted by the Ixodes tick, and are often transmitted together. Erlichia is more commonly transmitted by the lone star tick

## Slide 21
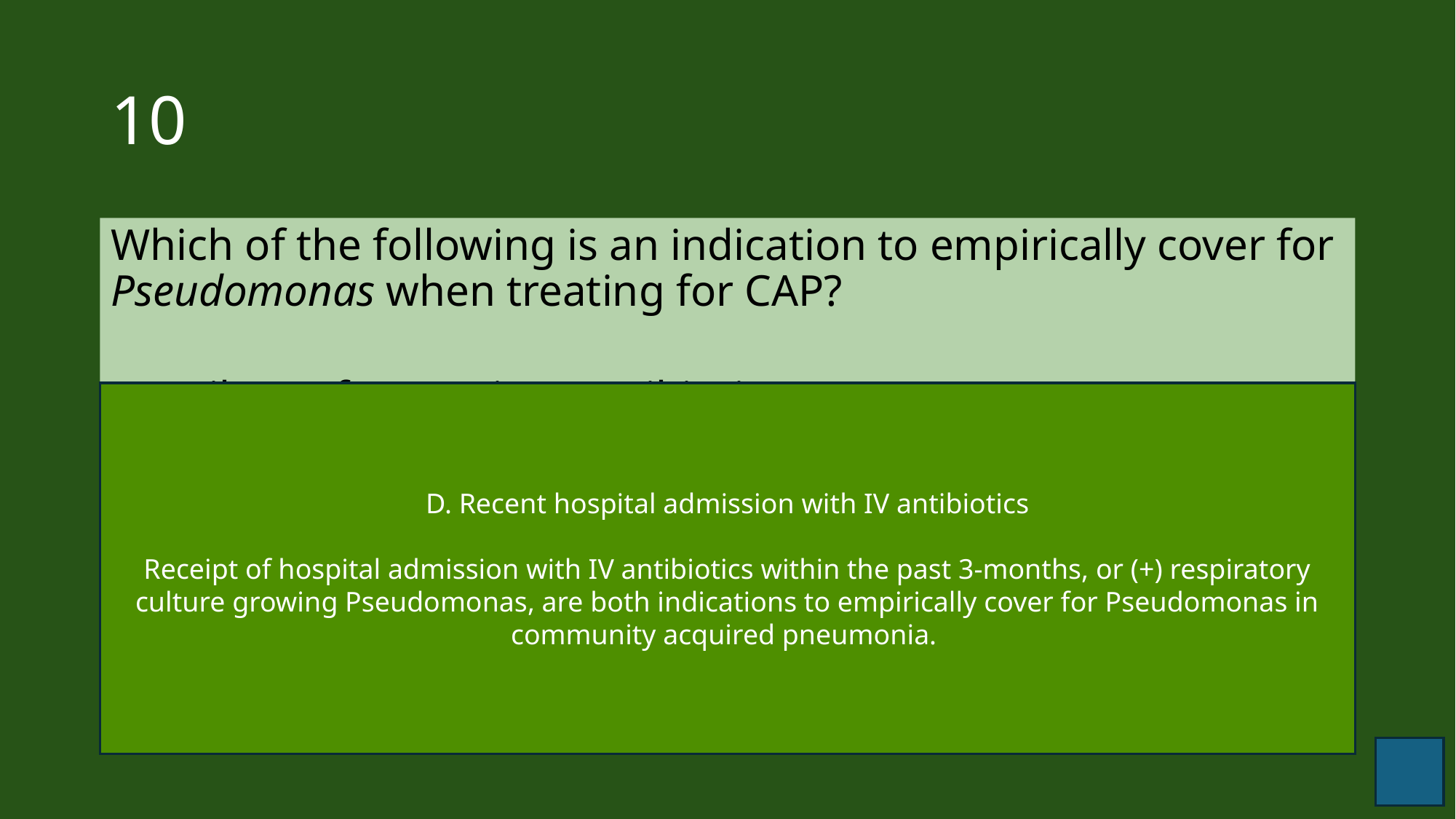

# 10
Which of the following is an indication to empirically cover for Pseudomonas when treating for CAP?
 Failure of outpatient antibiotics
 Chronic dialysis
 HIV
 Severe pneumonia and recent hospital admission with IV antibiotics
 Requiring mechanical ventilation
D. Recent hospital admission with IV antibiotics
Receipt of hospital admission with IV antibiotics within the past 3-months, or (+) respiratory culture growing Pseudomonas, are both indications to empirically cover for Pseudomonas in community acquired pneumonia.

## Slide 22
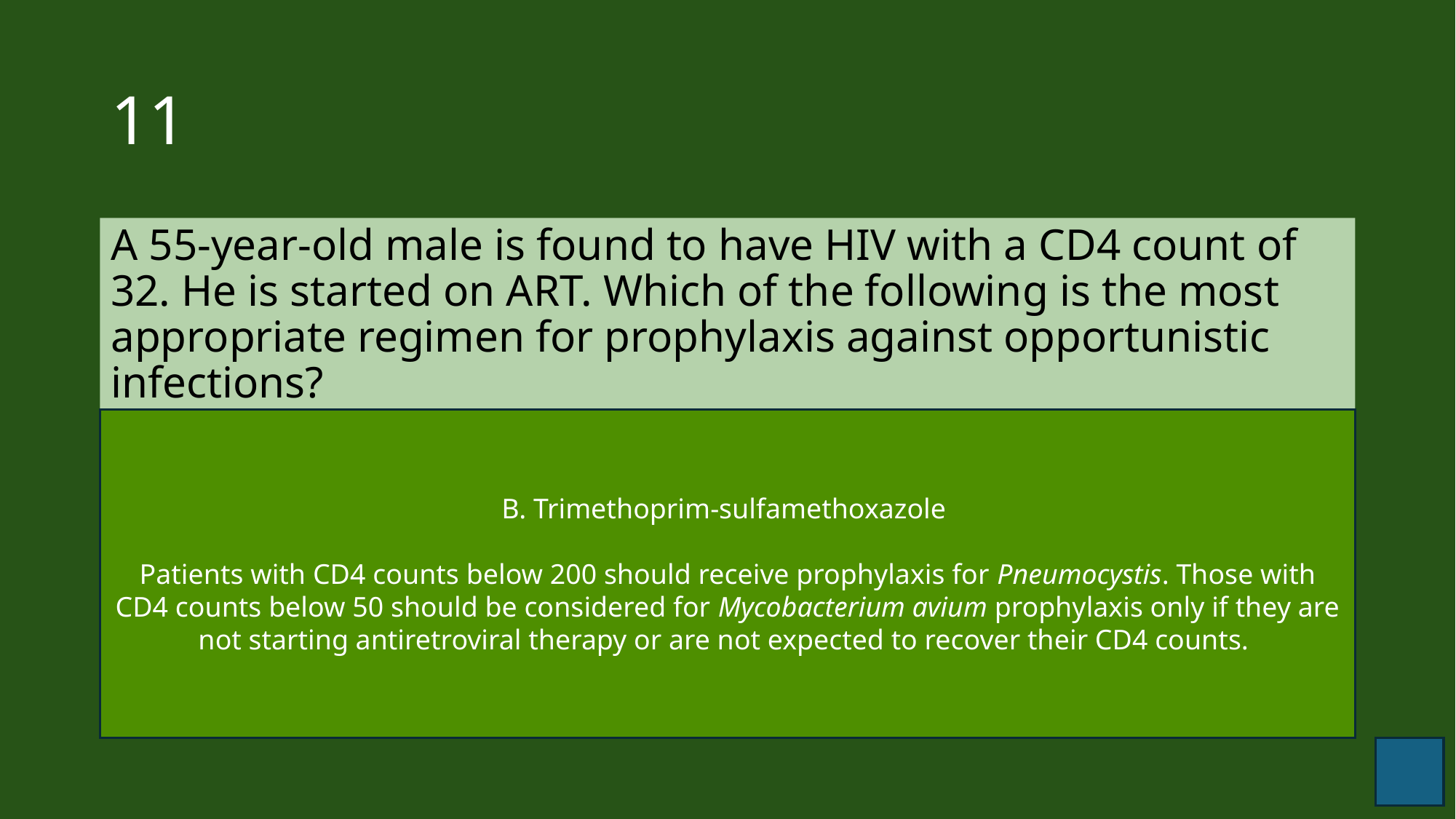

# 11
A 55-year-old male is found to have HIV with a CD4 count of 32. He is started on ART. Which of the following is the most appropriate regimen for prophylaxis against opportunistic infections?
Azithromycin
Trimethoprim-sulfamethoxazole
Azithromycin + trimethoprim-sulfamethoxazole
Azithromycin + Fluconazole
Azithromycin + Atovaquone
B. Trimethoprim-sulfamethoxazole
Patients with CD4 counts below 200 should receive prophylaxis for Pneumocystis. Those with CD4 counts below 50 should be considered for Mycobacterium avium prophylaxis only if they are not starting antiretroviral therapy or are not expected to recover their CD4 counts.

## Slide 23
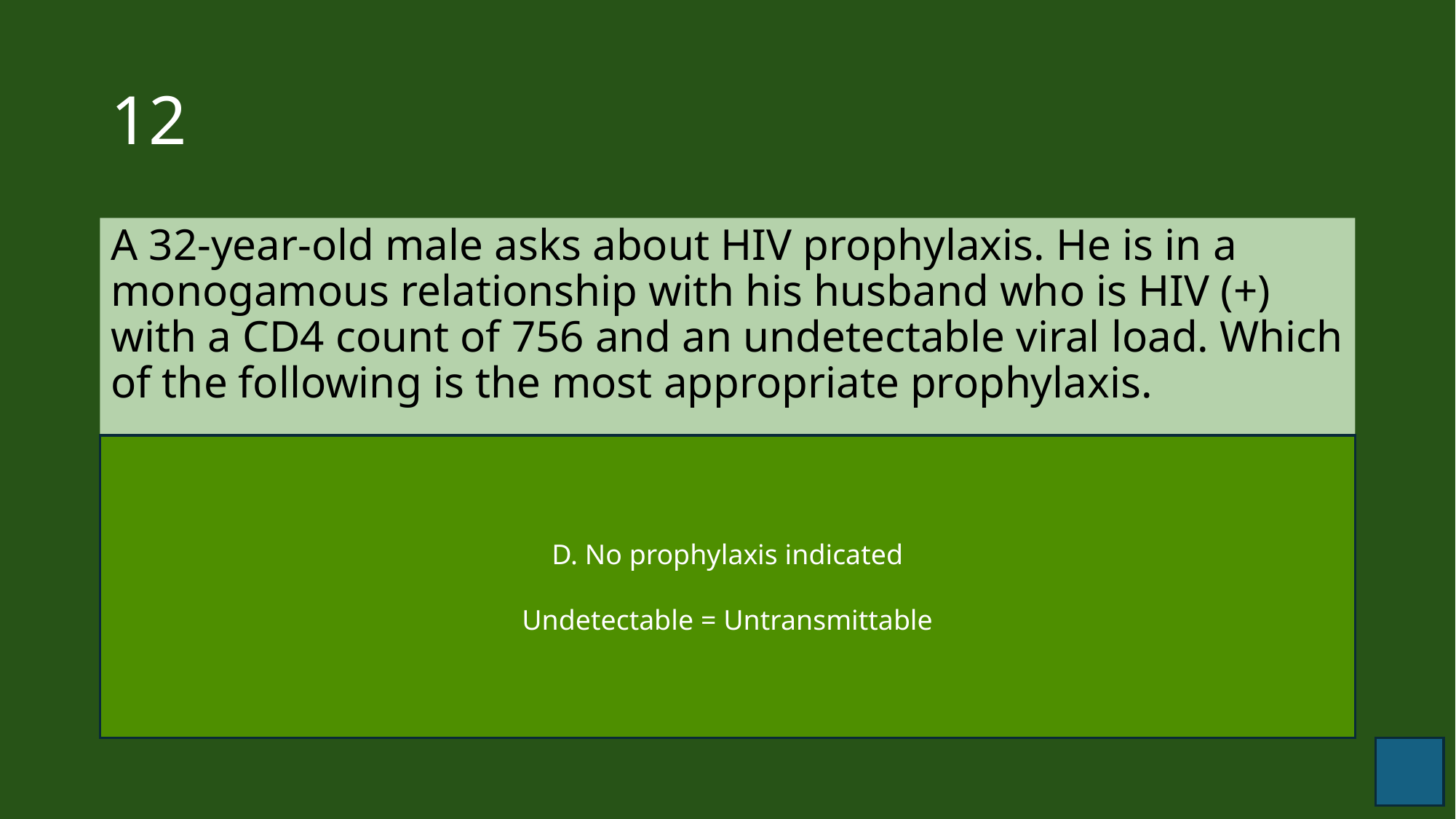

# 12
A 32-year-old male asks about HIV prophylaxis. He is in a monogamous relationship with his husband who is HIV (+) with a CD4 count of 756 and an undetectable viral load. Which of the following is the most appropriate prophylaxis.
Tenofovir + Emtricitabine daily
Tenofovir + emtricitabine on the day of intercourse and for the next two days following
IM cabotegravir bimonthly
No prophylaxis indicated
D. No prophylaxis indicated
Undetectable = Untransmittable

## Slide 24
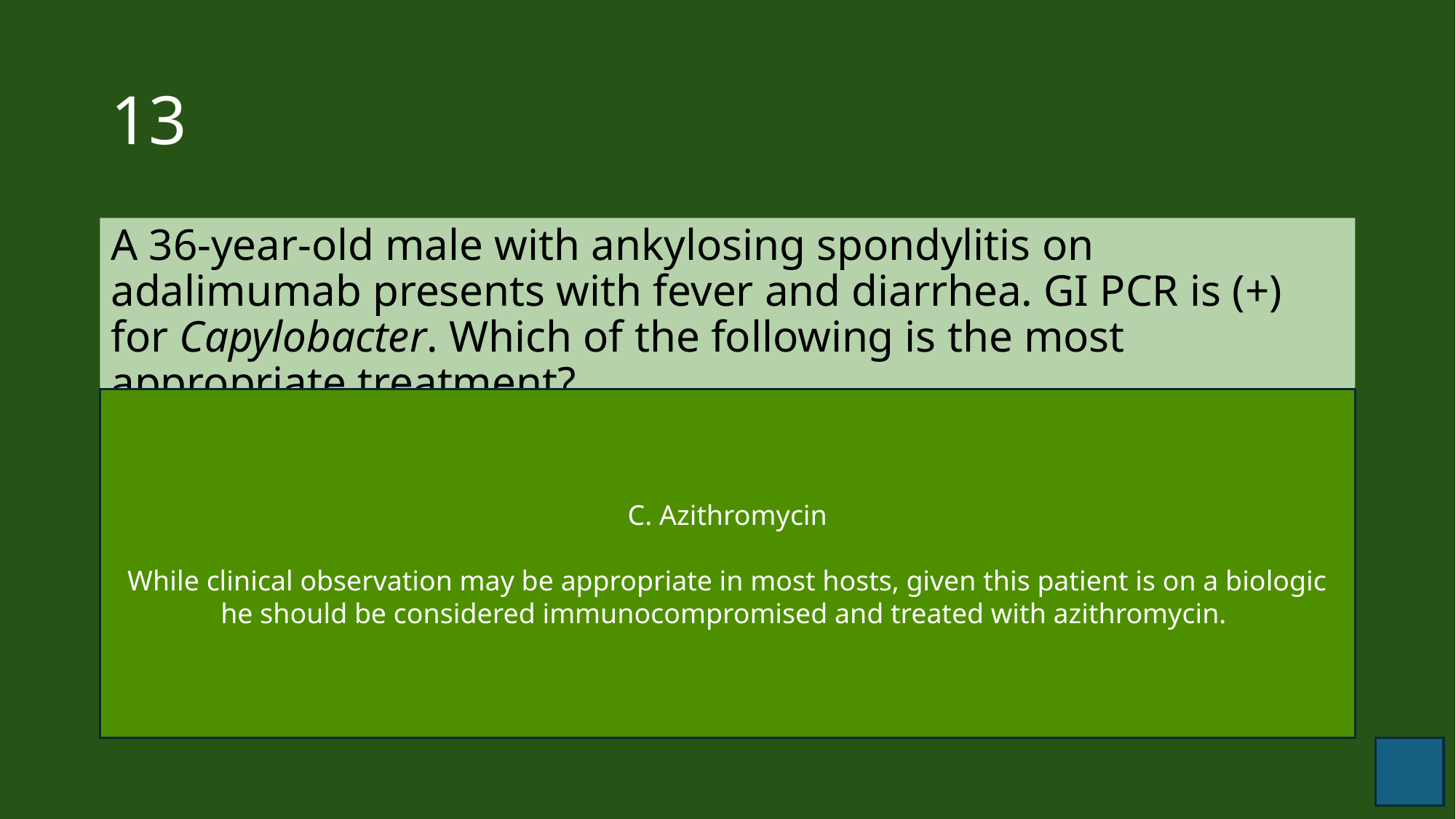

# 13
A 36-year-old male with ankylosing spondylitis on adalimumab presents with fever and diarrhea. GI PCR is (+) for Capylobacter. Which of the following is the most appropriate treatment?
Metronidazole
Fidaxomicin
Azithromycin
Ciprofloxacin
Clinical observation
C. Azithromycin
While clinical observation may be appropriate in most hosts, given this patient is on a biologic he should be considered immunocompromised and treated with azithromycin.

## Slide 25
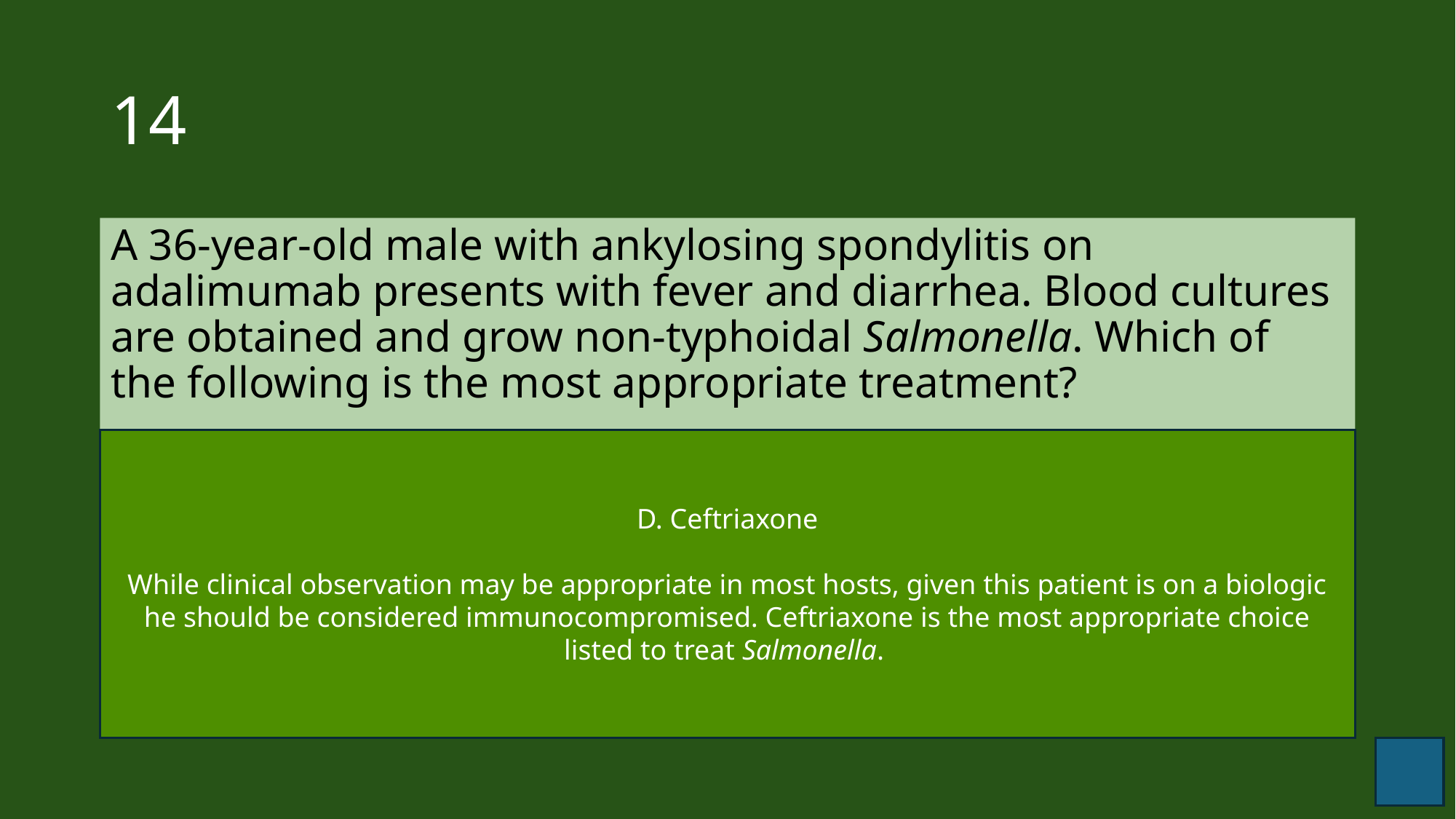

# 14
A 36-year-old male with ankylosing spondylitis on adalimumab presents with fever and diarrhea. Blood cultures are obtained and grow non-typhoidal Salmonella. Which of the following is the most appropriate treatment?
Metronidazole
Fidaxomicin
Azithromycin
Ceftriaxone
Clinical observation
D. Ceftriaxone
While clinical observation may be appropriate in most hosts, given this patient is on a biologic he should be considered immunocompromised. Ceftriaxone is the most appropriate choice listed to treat Salmonella.

## Slide 26
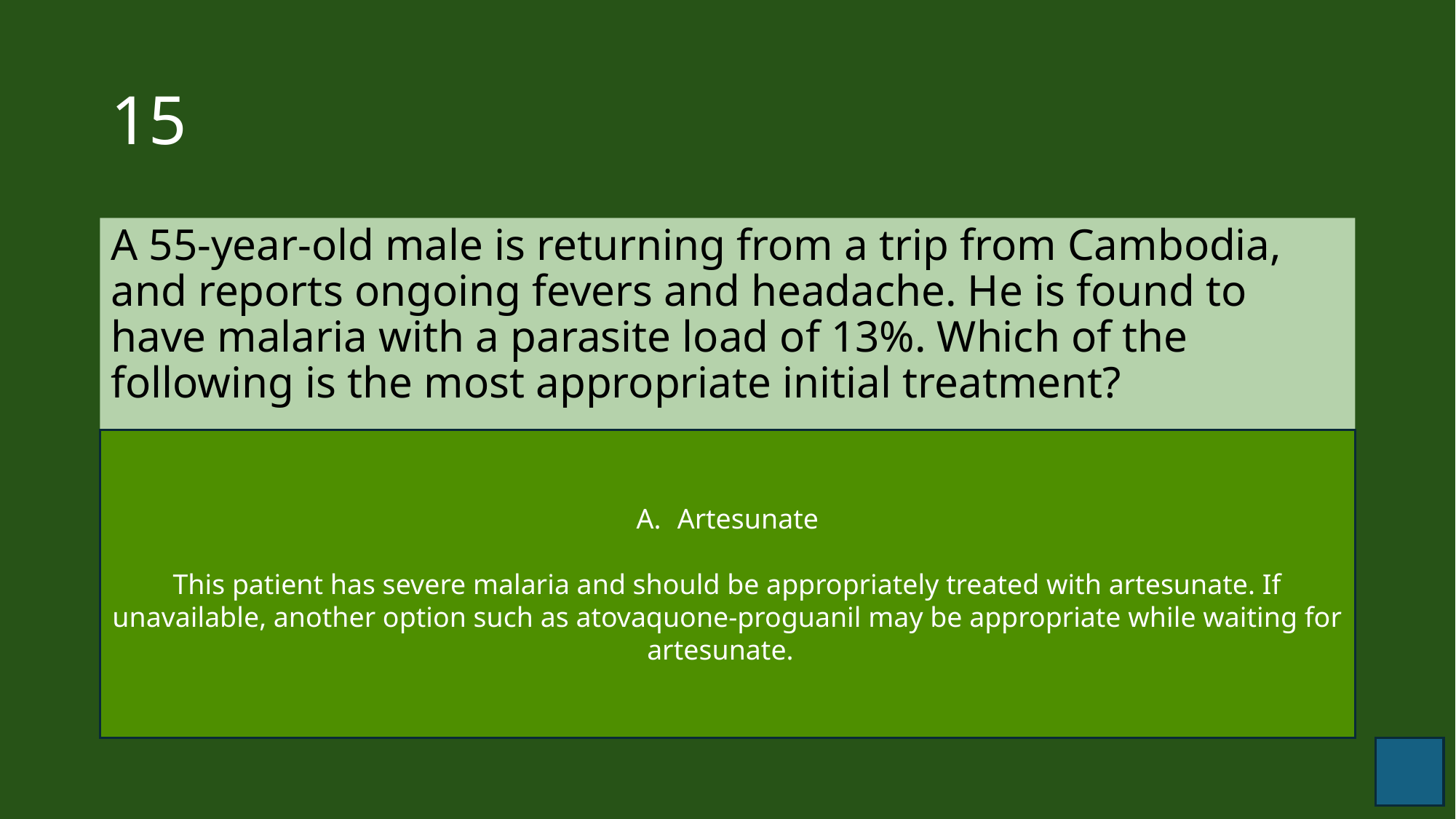

# 15
A 55-year-old male is returning from a trip from Cambodia, and reports ongoing fevers and headache. He is found to have malaria with a parasite load of 13%. Which of the following is the most appropriate initial treatment?
Artesunate
Doxycycline and clindamycin
Primaquine
Atovaquone-proguanil
Artesunate
This patient has severe malaria and should be appropriately treated with artesunate. If unavailable, another option such as atovaquone-proguanil may be appropriate while waiting for artesunate.

## Slide 27
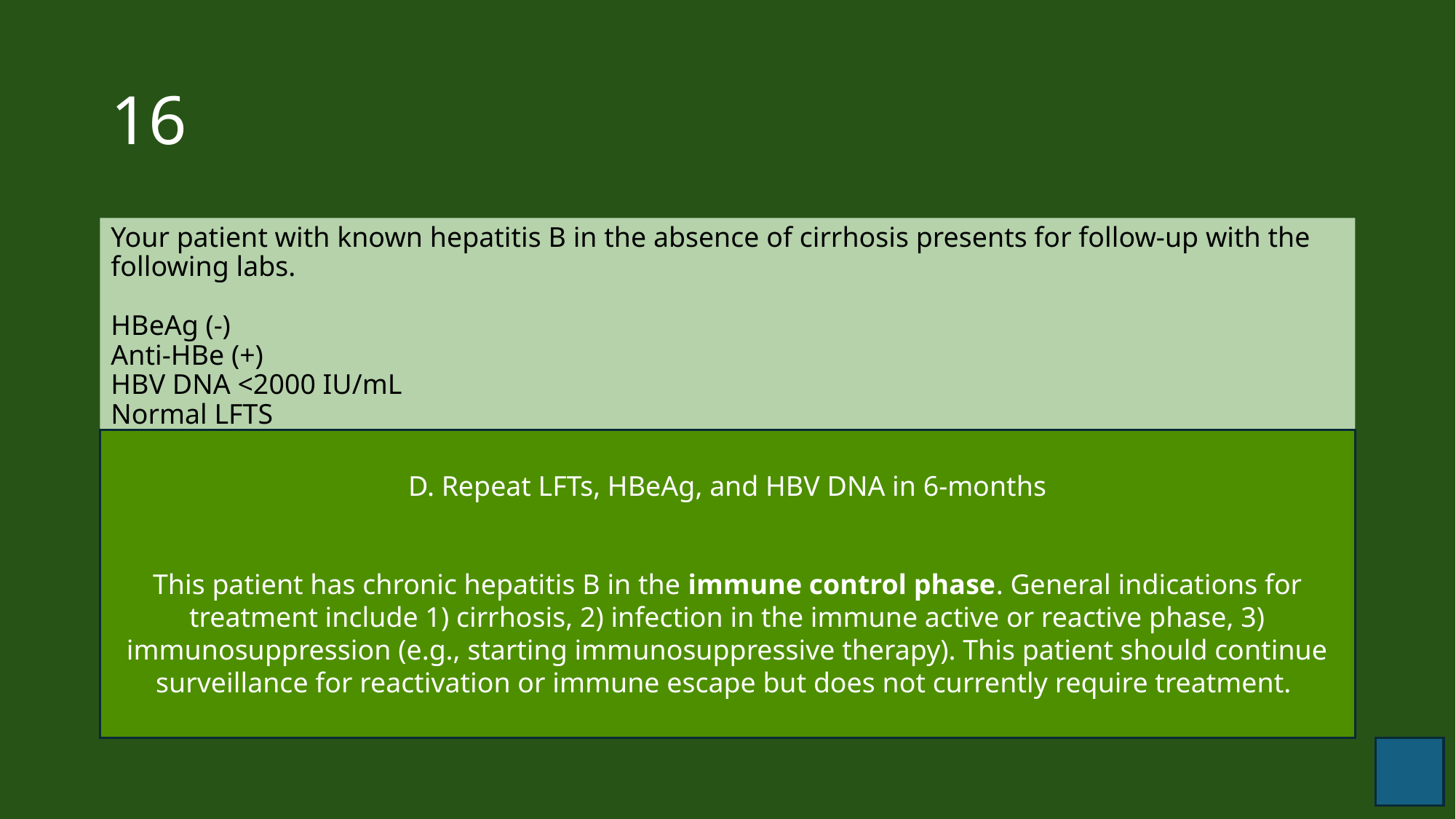

# 16
Your patient with known hepatitis B in the absence of cirrhosis presents for follow-up with the following labs.
HBeAg (-)
Anti-HBe (+)
HBV DNA <2000 IU/mL
Normal LFTS
What is the best next step in management for this patient?
Start tenofovir
Start entecavir
Start lamivudine
Repeat LFTs, HBeAg, and HBV DNA in 6-months
D. Repeat LFTs, HBeAg, and HBV DNA in 6-months
This patient has chronic hepatitis B in the immune control phase. General indications for treatment include 1) cirrhosis, 2) infection in the immune active or reactive phase, 3) immunosuppression (e.g., starting immunosuppressive therapy). This patient should continue surveillance for reactivation or immune escape but does not currently require treatment.

## Slide 28
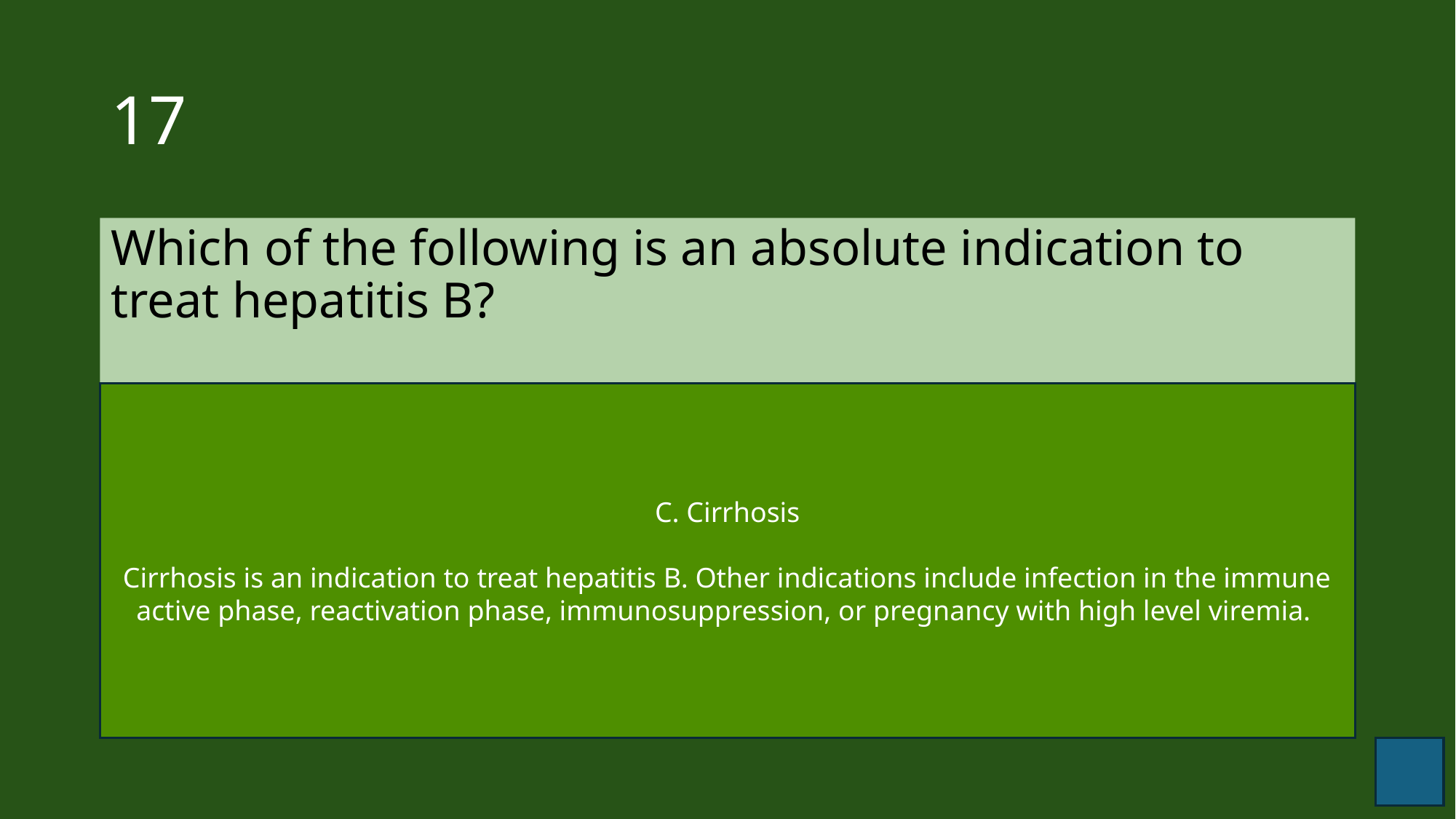

# 17
Which of the following is an absolute indication to treat hepatitis B?
Infection in the immune tolerant phase
Pregnancy
Cirrhosis
Anti-HBe seroconversion
C. Cirrhosis
Cirrhosis is an indication to treat hepatitis B. Other indications include infection in the immune active phase, reactivation phase, immunosuppression, or pregnancy with high level viremia.

## Slide 29
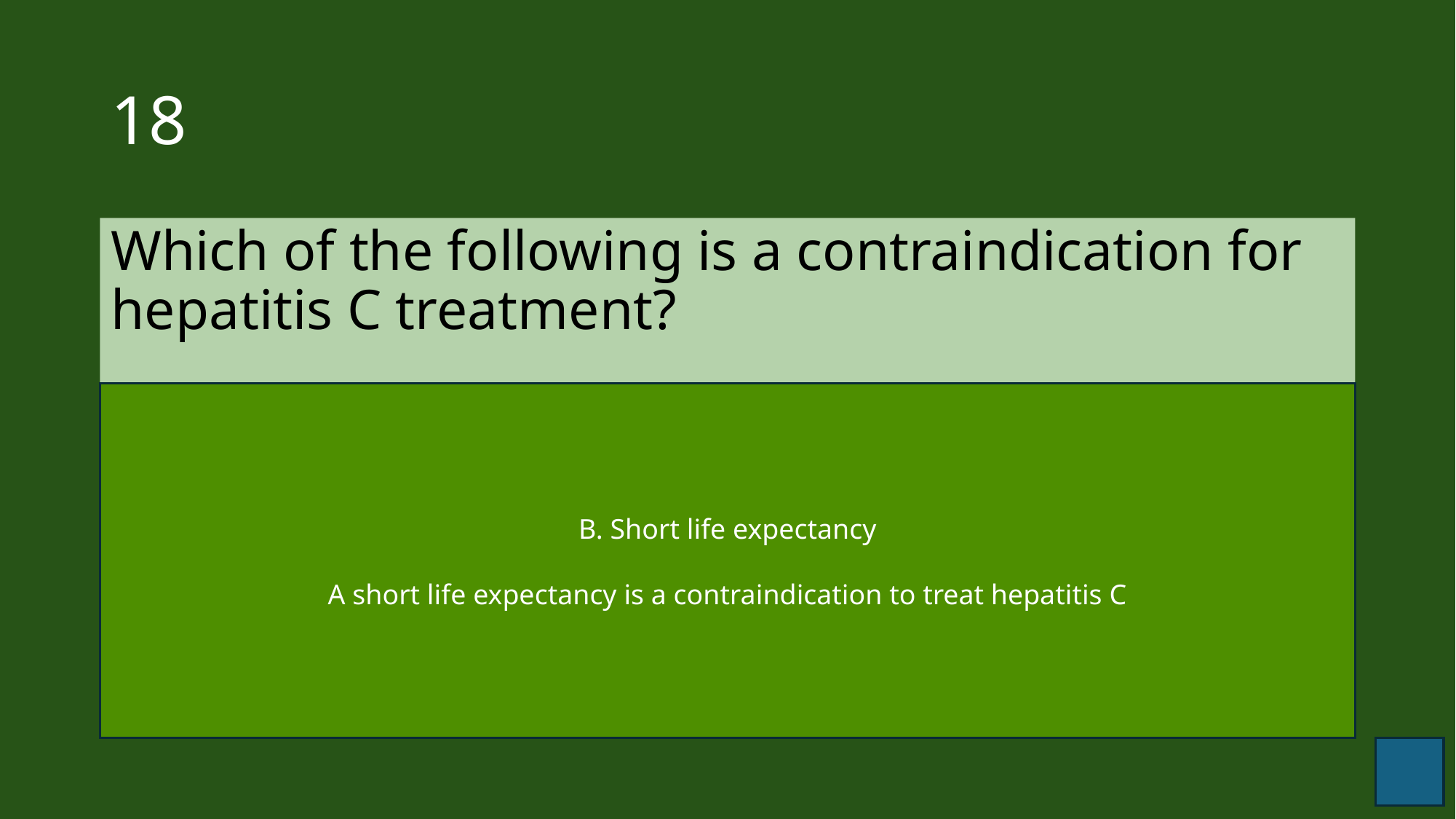

# 18
Which of the following is a contraindication for hepatitis C treatment?
Anticipated liver transplantation
Short life expectancy
Absent liver fibrosis or cirrhosis
Anti-HCV seroconversion
B. Short life expectancy
A short life expectancy is a contraindication to treat hepatitis C

## Slide 30
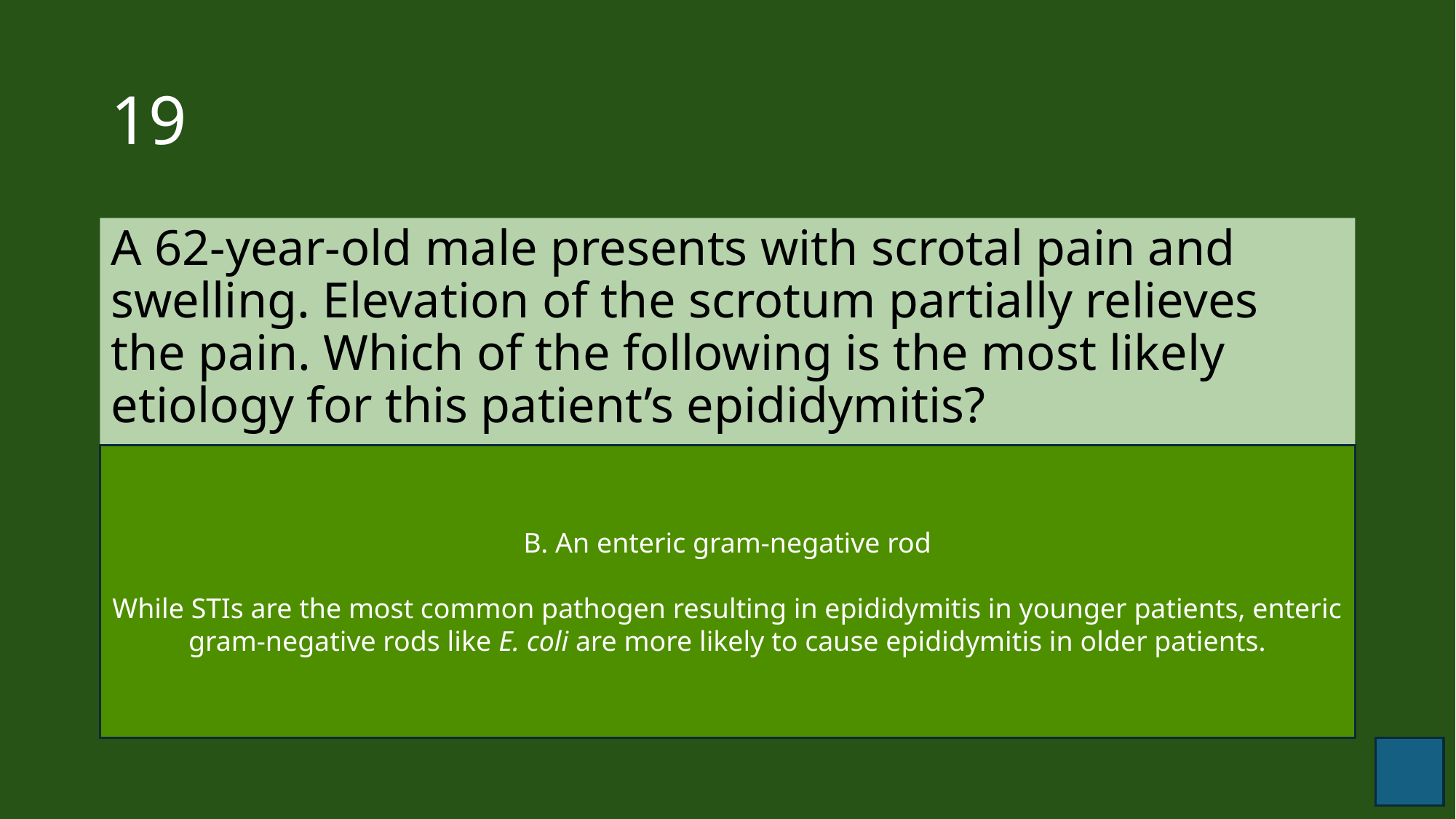

# 19
A 62-year-old male presents with scrotal pain and swelling. Elevation of the scrotum partially relieves the pain. Which of the following is the most likely etiology for this patient’s epididymitis?
 An STI
 An enteric gram-negative rod infection
 A hematogenous infection
 A vaccine-preventable viral infection
B. An enteric gram-negative rod
While STIs are the most common pathogen resulting in epididymitis in younger patients, enteric gram-negative rods like E. coli are more likely to cause epididymitis in older patients.

## Slide 31
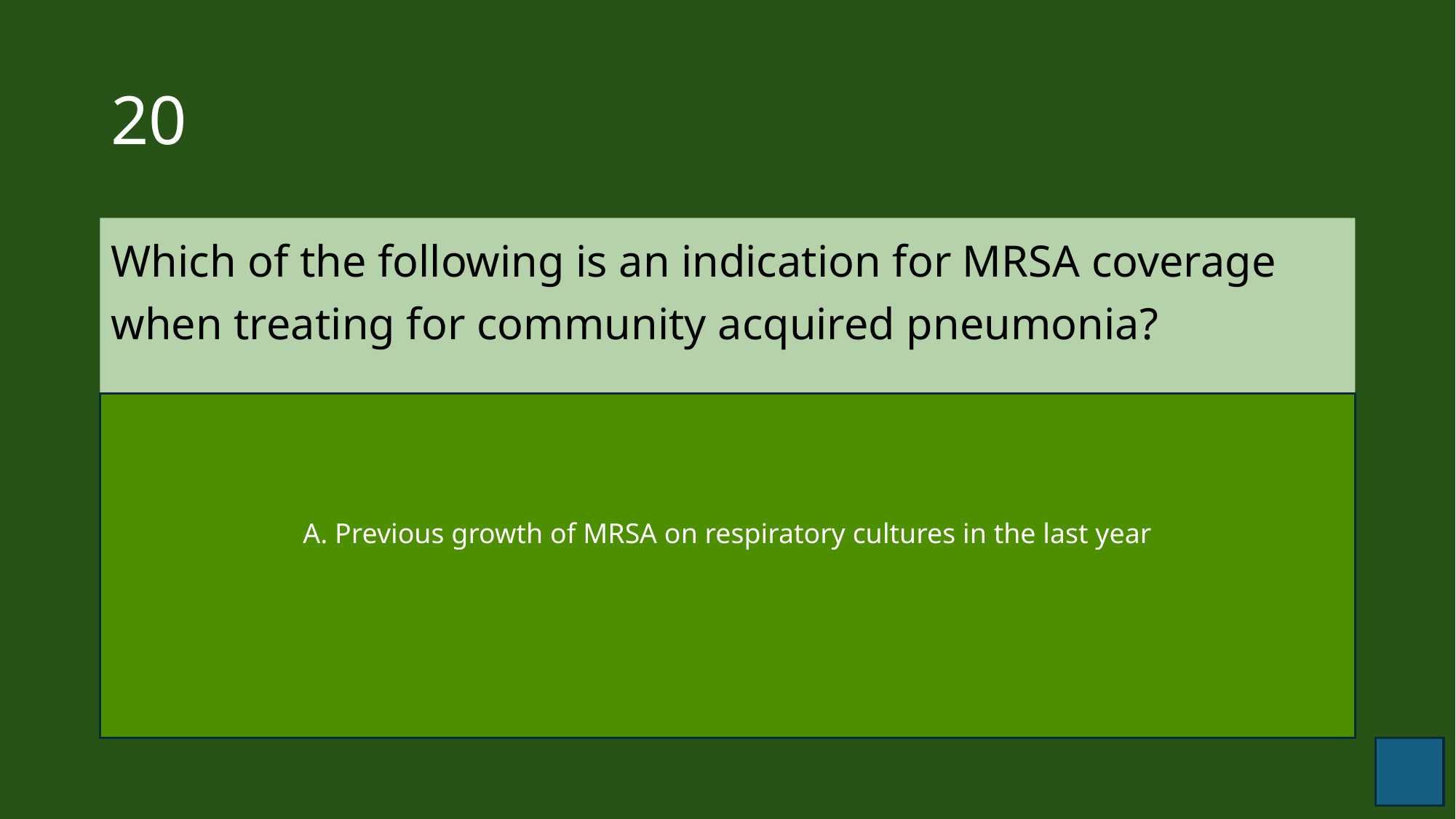

# 20
Which of the following is an indication for MRSA coverage when treating for community acquired pneumonia?
 Previous growth of MRSA on respiratory culture in the past year
 Parenteral antibiotics within the last 6-months
 Failure of outpatient antibiotics
 Poor dentition
A. Previous growth of MRSA on respiratory cultures in the last year

## Slide 32
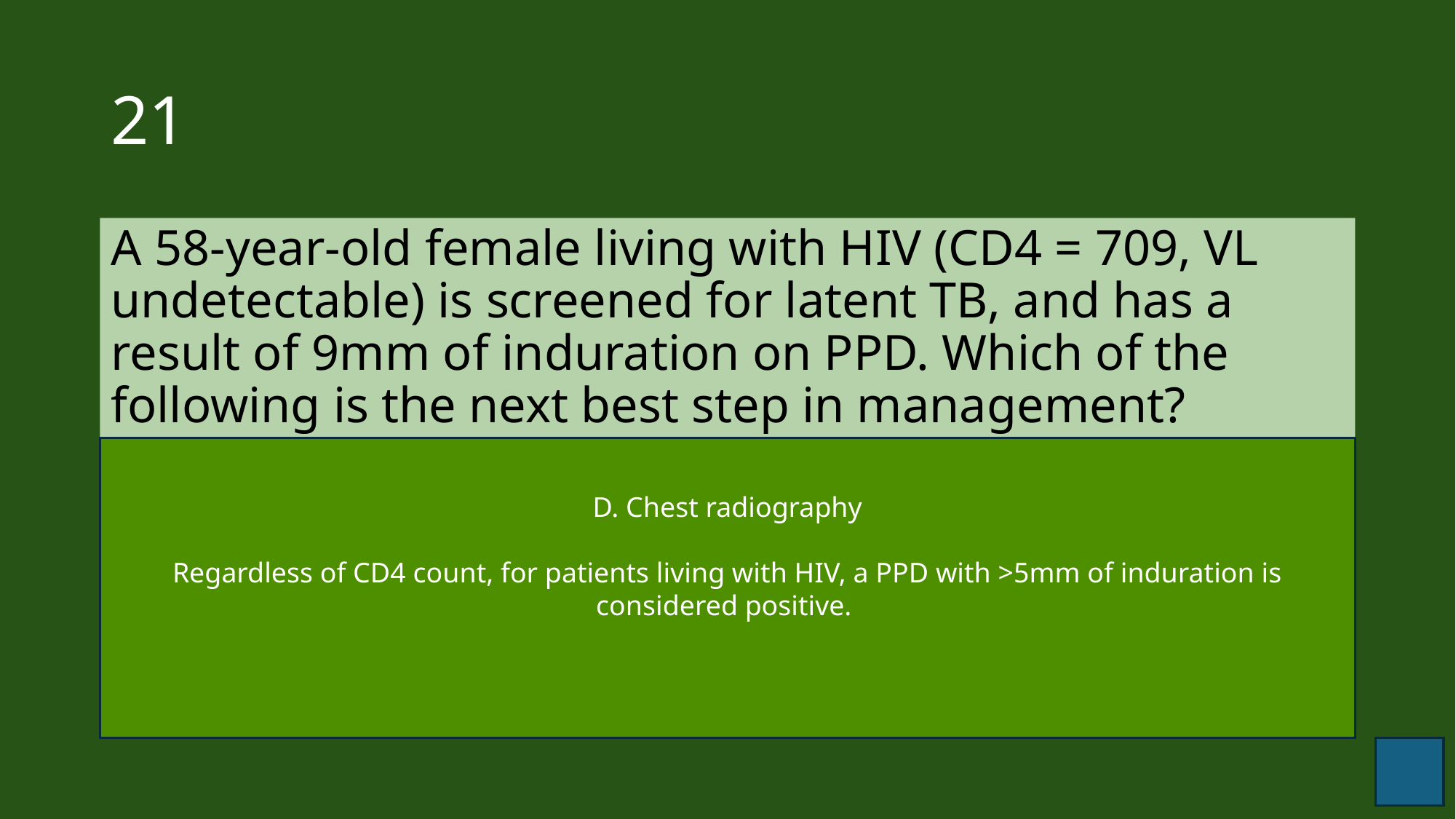

# 21
A 58-year-old female living with HIV (CD4 = 709, VL undetectable) is screened for latent TB, and has a result of 9mm of induration on PPD. Which of the following is the next best step in management?
Return to routine screening
Rifampin daily for 4-months
Isoniazid and rifampin daily for 3-months
Chest radiography
D. Chest radiography
Regardless of CD4 count, for patients living with HIV, a PPD with >5mm of induration is considered positive.

## Slide 33
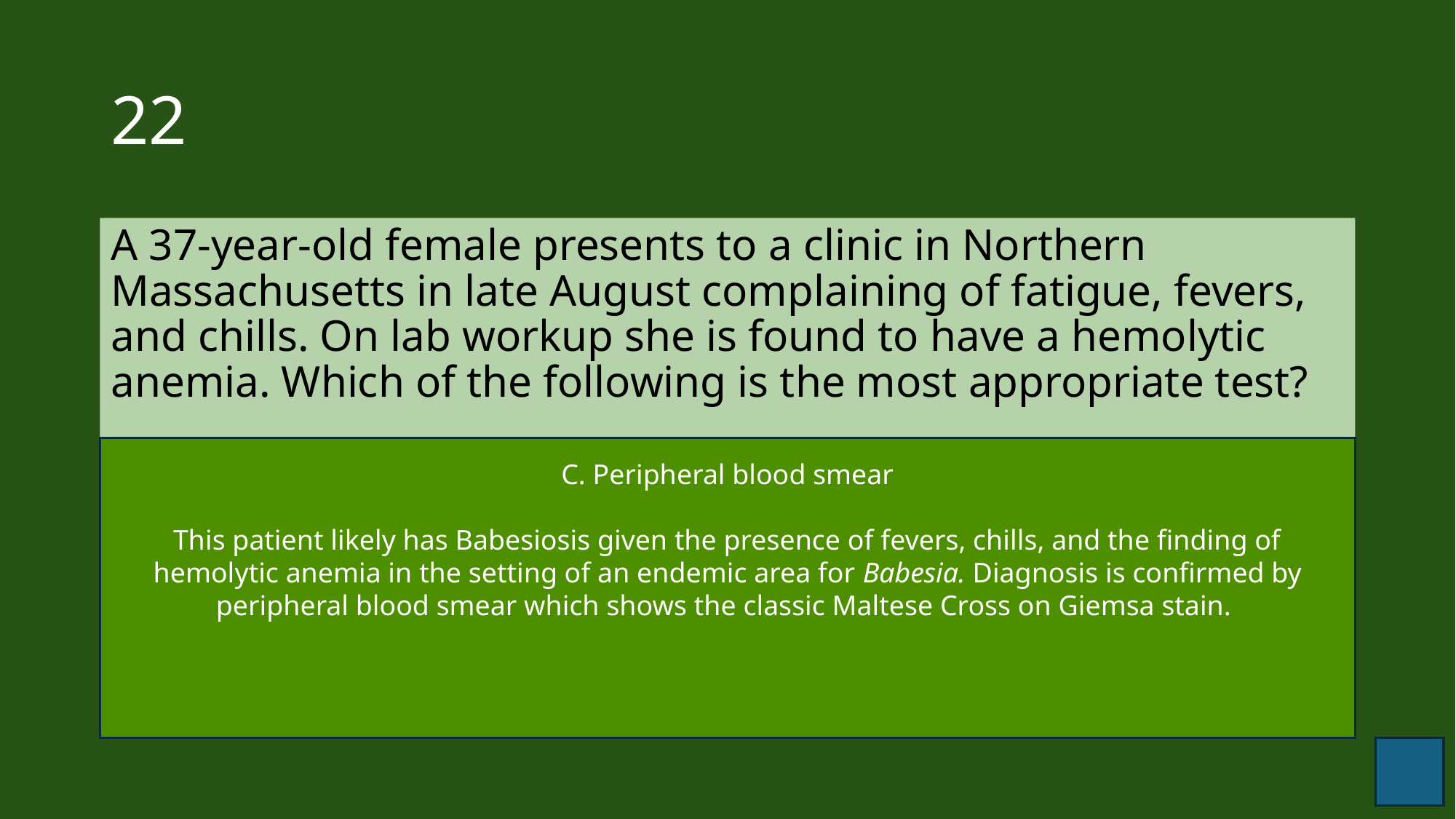

# 22
A 37-year-old female presents to a clinic in Northern Massachusetts in late August complaining of fatigue, fevers, and chills. On lab workup she is found to have a hemolytic anemia. Which of the following is the most appropriate test?
 ELISA antibody testing
 Western blot testing
 Peripheral blood smear
 Blood cultures
C. Peripheral blood smear
This patient likely has Babesiosis given the presence of fevers, chills, and the finding of hemolytic anemia in the setting of an endemic area for Babesia. Diagnosis is confirmed by peripheral blood smear which shows the classic Maltese Cross on Giemsa stain.

## Slide 34
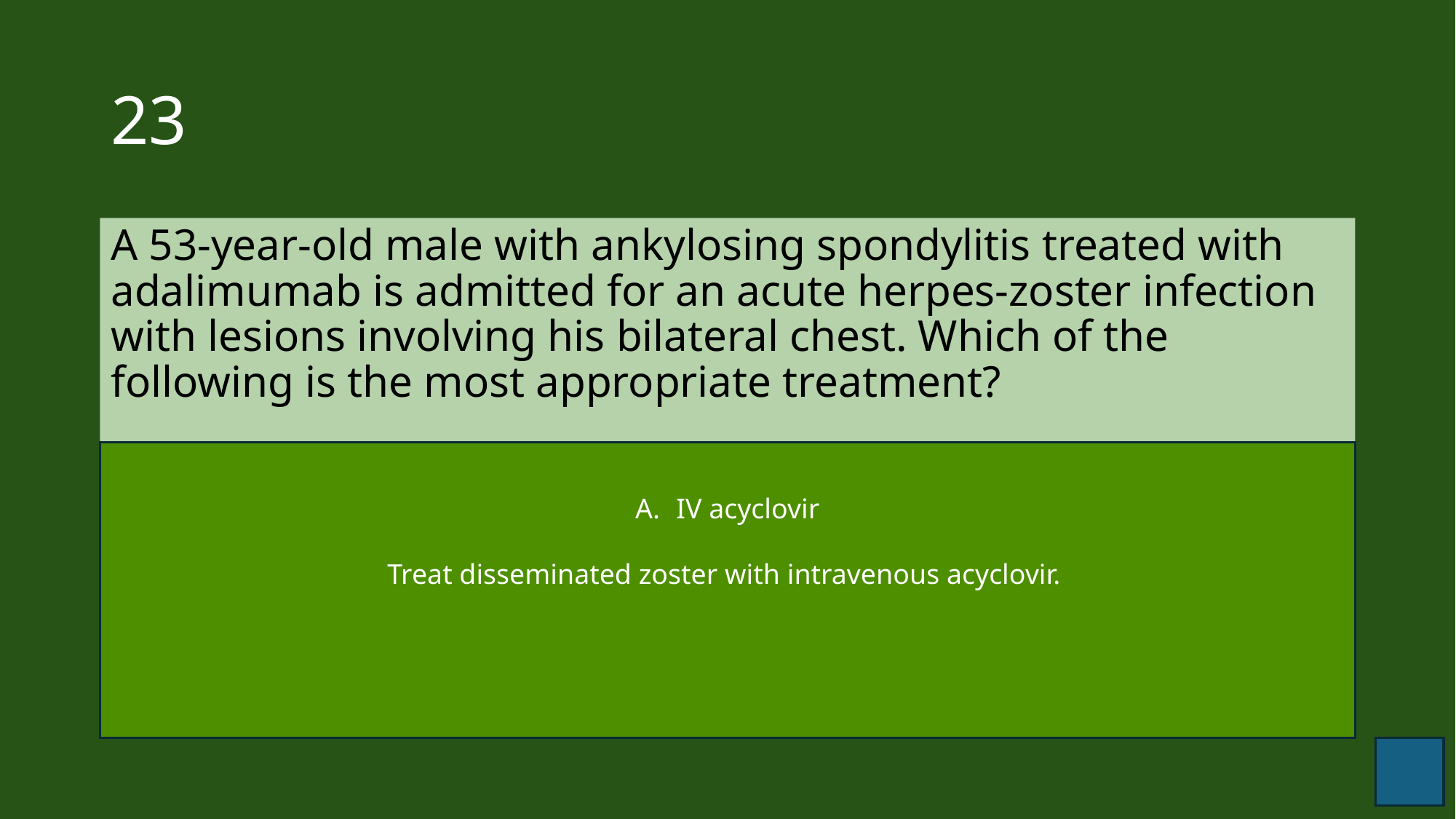

# 23
A 53-year-old male with ankylosing spondylitis treated with adalimumab is admitted for an acute herpes-zoster infection with lesions involving his bilateral chest. Which of the following is the most appropriate treatment?
A. IV acyclovir
B. PO valacyclovir
C. PO ganciclovir
D. PO famciclovir
E. Withdrawal of adalimumab; clinical observation
IV acyclovir
Treat disseminated zoster with intravenous acyclovir.

## Slide 35
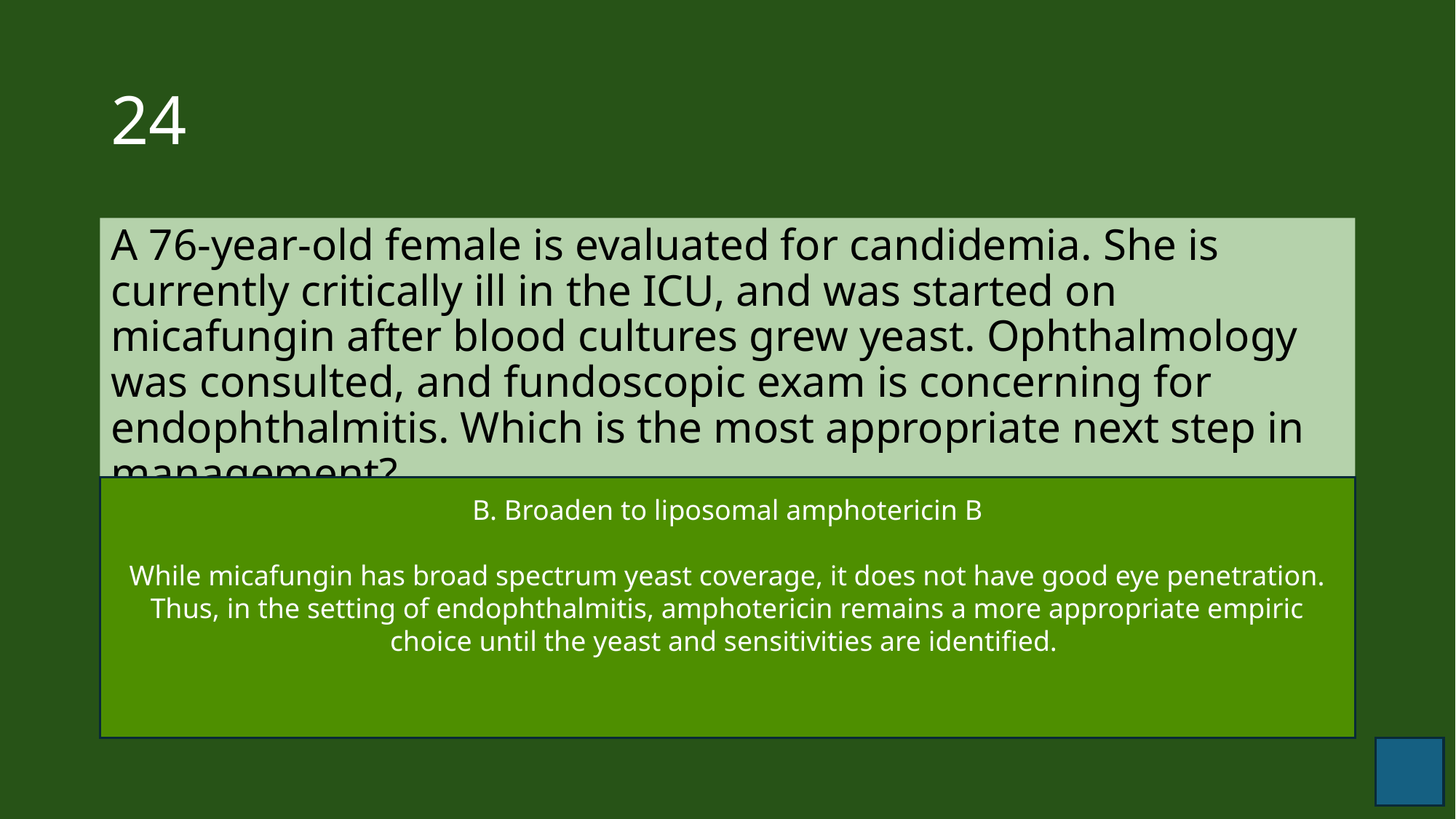

# 24
A 76-year-old female is evaluated for candidemia. She is currently critically ill in the ICU, and was started on micafungin after blood cultures grew yeast. Ophthalmology was consulted, and fundoscopic exam is concerning for endophthalmitis. Which is the most appropriate next step in management?
 Continue with micafungin
 Broaden to liposomal amphotericin B
 Narrow to fluconazole
 Switch to terbinafine
B. Broaden to liposomal amphotericin B
While micafungin has broad spectrum yeast coverage, it does not have good eye penetration. Thus, in the setting of endophthalmitis, amphotericin remains a more appropriate empiric choice until the yeast and sensitivities are identified.

## Slide 36
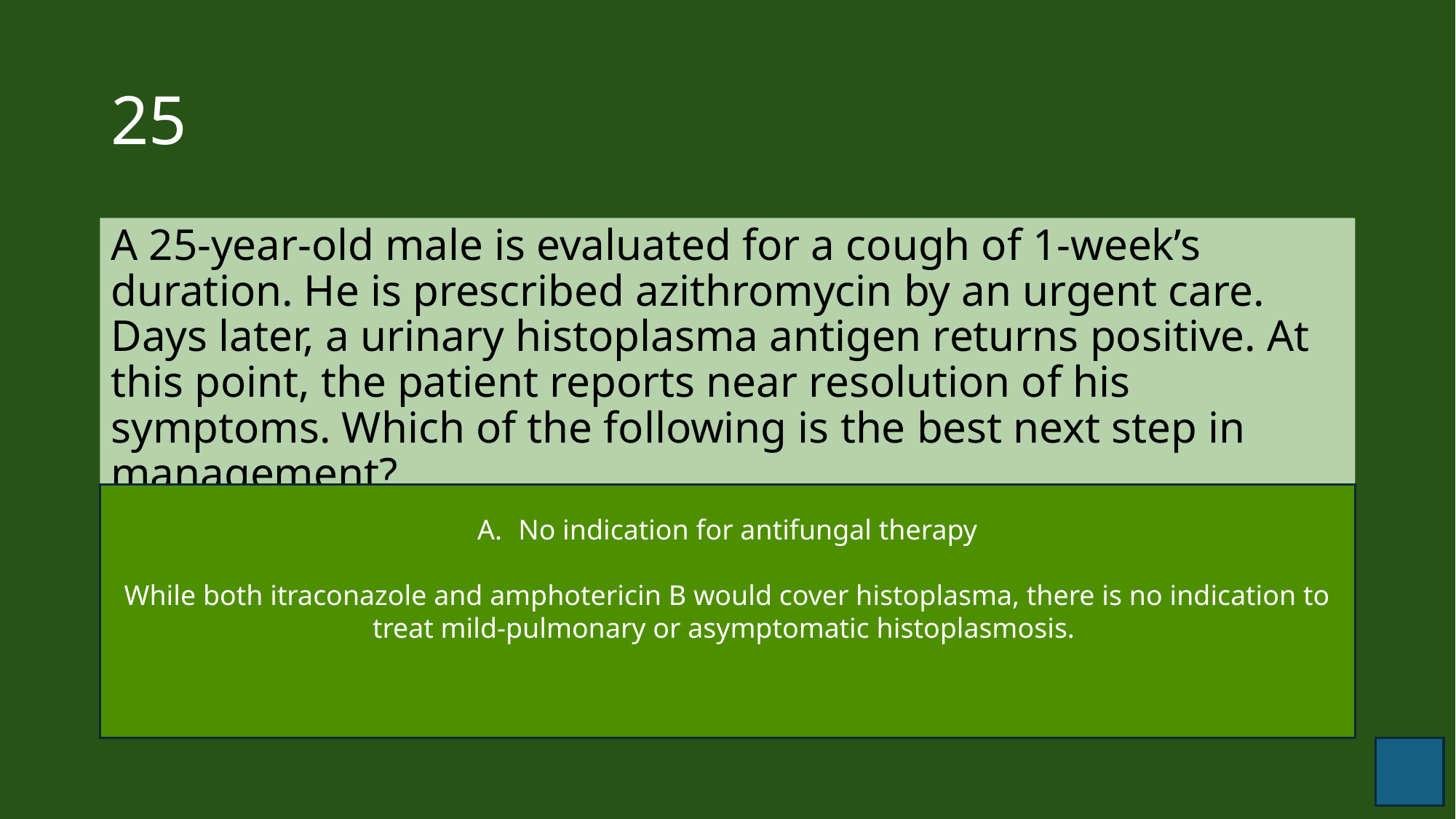

# 25
A 25-year-old male is evaluated for a cough of 1-week’s duration. He is prescribed azithromycin by an urgent care. Days later, a urinary histoplasma antigen returns positive. At this point, the patient reports near resolution of his symptoms. Which of the following is the best next step in management?
No indication for anti-fungal therapy
Itraconazole
Fluconazole
Liposomal amphotericin B
No indication for antifungal therapy
While both itraconazole and amphotericin B would cover histoplasma, there is no indication to treat mild-pulmonary or asymptomatic histoplasmosis.

## Slide 37
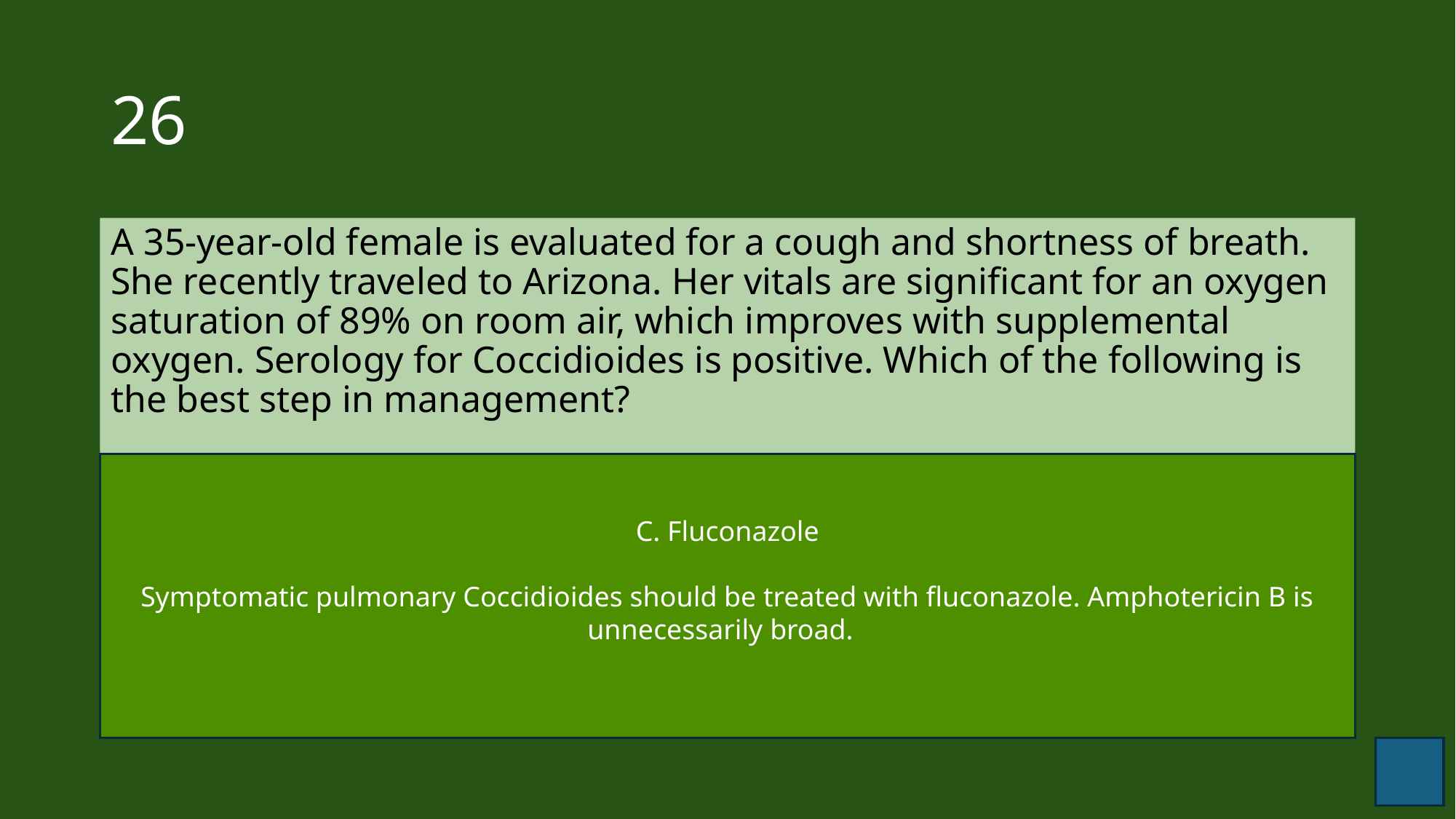

# 26
A 35-year-old female is evaluated for a cough and shortness of breath. She recently traveled to Arizona. Her vitals are significant for an oxygen saturation of 89% on room air, which improves with supplemental oxygen. Serology for Coccidioides is positive. Which of the following is the best step in management?
No indication for anti-fungal therapy
Ketoconazole
Fluconazole
Liposomal amphotericin B
C. Fluconazole
Symptomatic pulmonary Coccidioides should be treated with fluconazole. Amphotericin B is unnecessarily broad.

## Slide 38
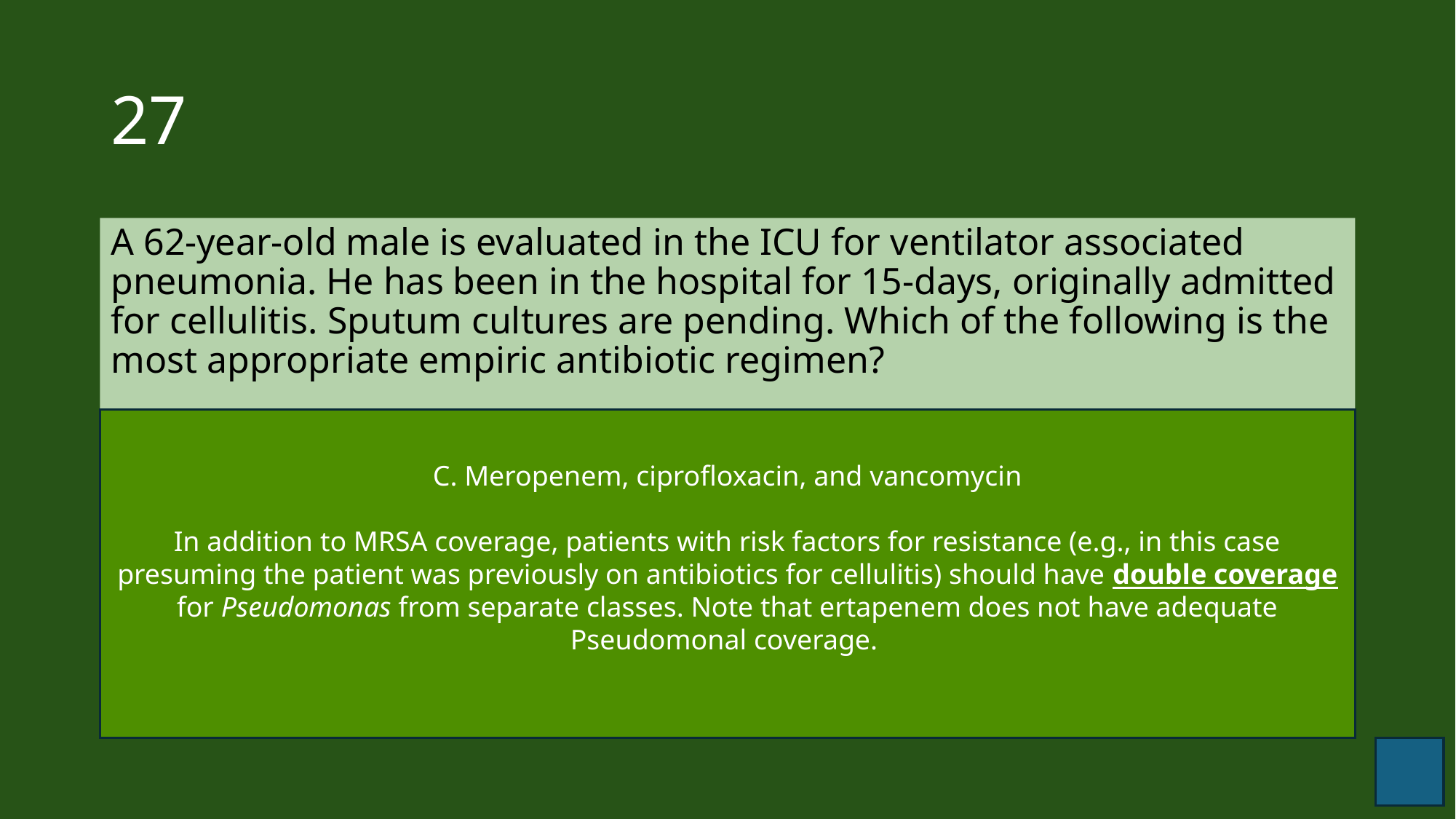

# 27
A 62-year-old male is evaluated in the ICU for ventilator associated pneumonia. He has been in the hospital for 15-days, originally admitted for cellulitis. Sputum cultures are pending. Which of the following is the most appropriate empiric antibiotic regimen?
Ertapenem, ciprofloxacin, and vancomycin
Meropenem and vancomycin
Meropenem, ciprofloxacin, and vancomycin
Piperacillin-tazobactam and linezolid
C. Meropenem, ciprofloxacin, and vancomycin
In addition to MRSA coverage, patients with risk factors for resistance (e.g., in this case presuming the patient was previously on antibiotics for cellulitis) should have double coverage for Pseudomonas from separate classes. Note that ertapenem does not have adequate Pseudomonal coverage.

## Slide 39
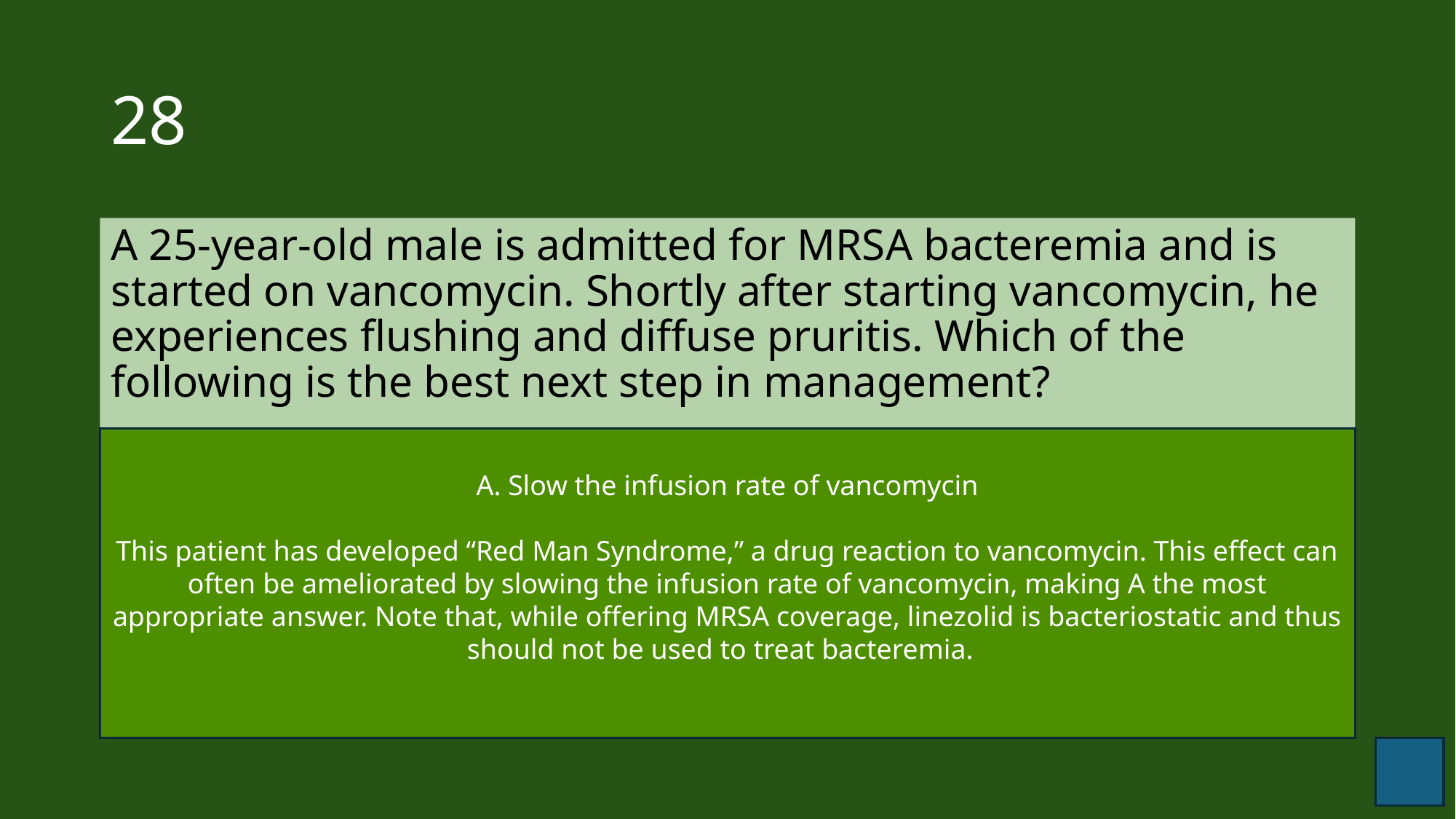

# 28
A 25-year-old male is admitted for MRSA bacteremia and is started on vancomycin. Shortly after starting vancomycin, he experiences flushing and diffuse pruritis. Which of the following is the best next step in management?
 Slow the infusion rate of vancomycin
 Discontinue vancomycin and treat with linezolid
 Give IM epinephrine
 Repeat blood cultures
A. Slow the infusion rate of vancomycin
This patient has developed “Red Man Syndrome,” a drug reaction to vancomycin. This effect can often be ameliorated by slowing the infusion rate of vancomycin, making A the most appropriate answer. Note that, while offering MRSA coverage, linezolid is bacteriostatic and thus should not be used to treat bacteremia.

## Slide 40
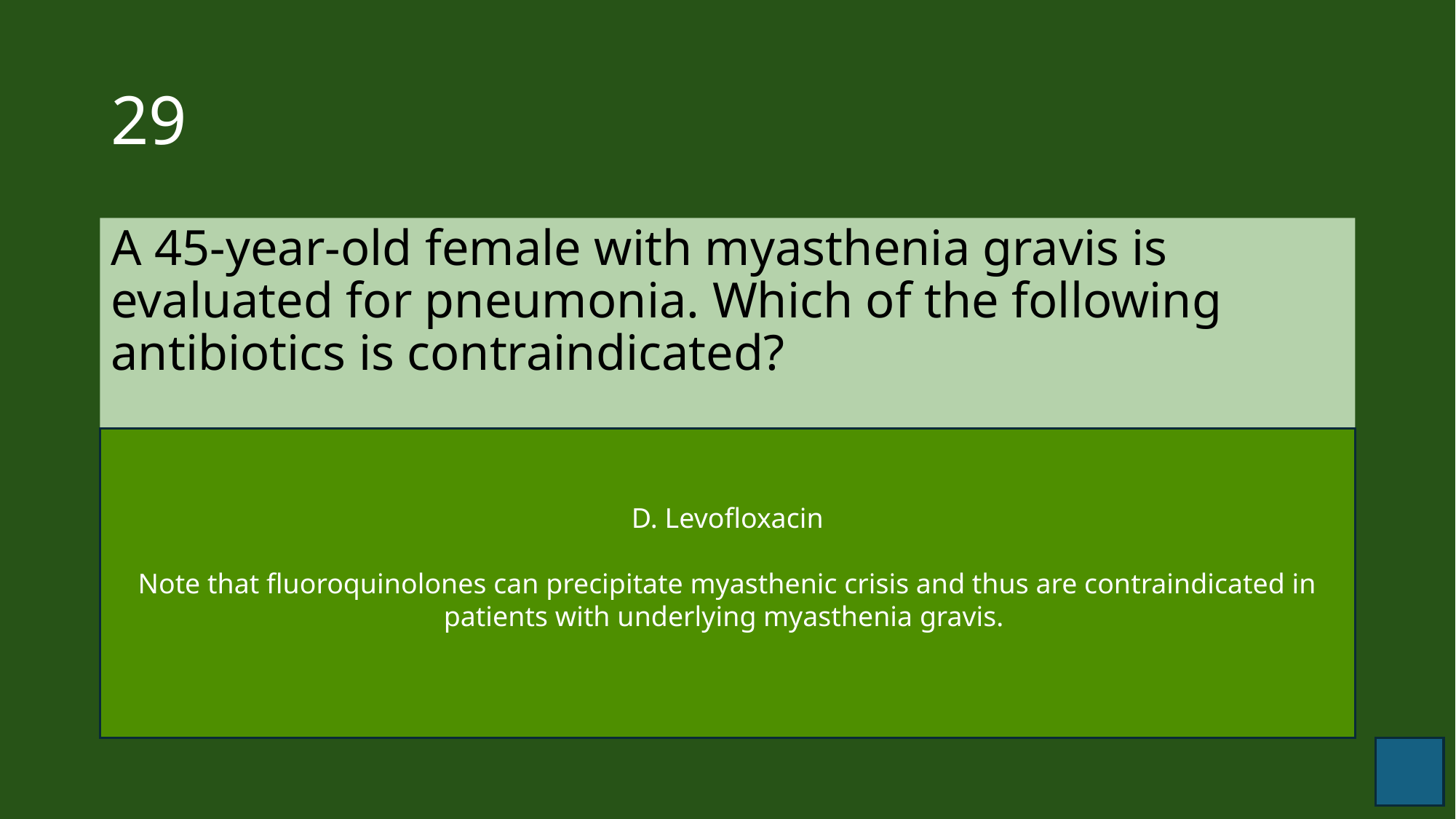

# 29
A 45-year-old female with myasthenia gravis is evaluated for pneumonia. Which of the following antibiotics is contraindicated?
 Ceftriaxone
 Doxycycline
 Meropenem
 Levofloxacin
D. Levofloxacin
Note that fluoroquinolones can precipitate myasthenic crisis and thus are contraindicated in patients with underlying myasthenia gravis.

## Slide 41
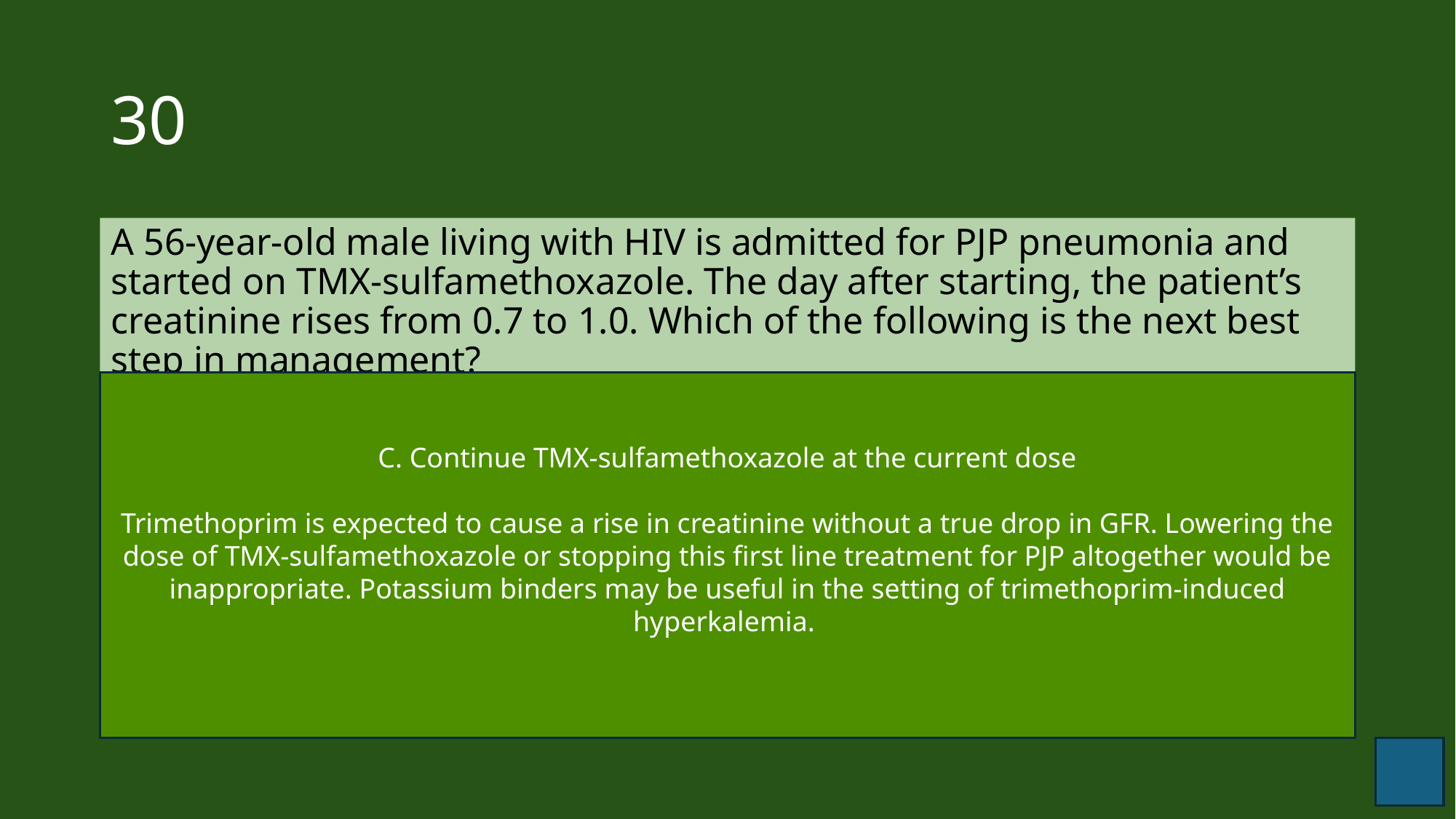

# 30
A 56-year-old male living with HIV is admitted for PJP pneumonia and started on TMX-sulfamethoxazole. The day after starting, the patient’s creatinine rises from 0.7 to 1.0. Which of the following is the next best step in management?
Stop TMX-sulfamethoxazole and start alternative treatment
Continue TMX-sulfamethoxazole at a lower dose
Continue TMX-sulfamethoxazole and the current therapeutic dose
Continue TMX-sulfamethoxazole and start sodium zirconium
C. Continue TMX-sulfamethoxazole at the current dose
Trimethoprim is expected to cause a rise in creatinine without a true drop in GFR. Lowering the dose of TMX-sulfamethoxazole or stopping this first line treatment for PJP altogether would be inappropriate. Potassium binders may be useful in the setting of trimethoprim-induced hyperkalemia.

## Slide 42
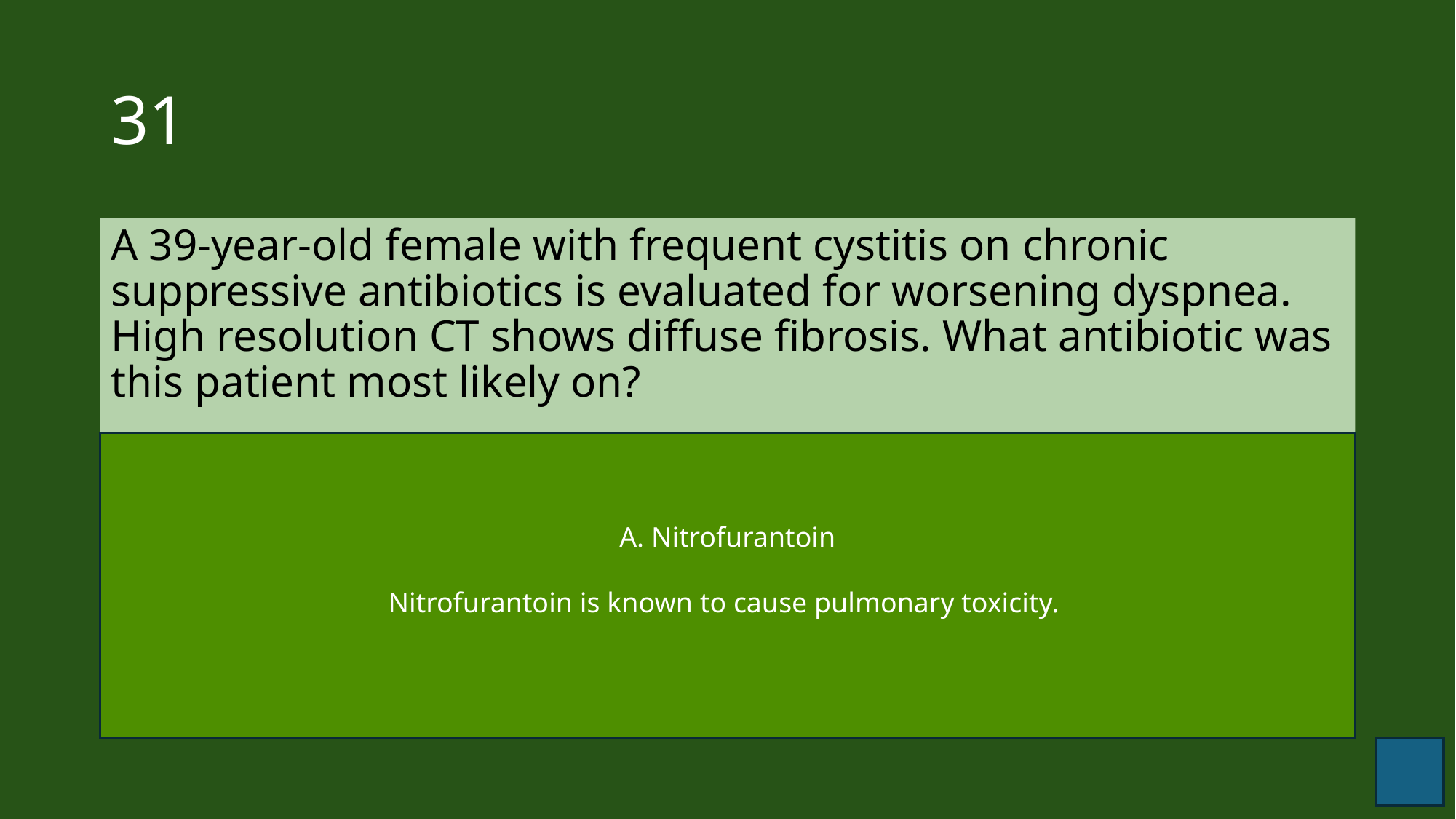

# 31
A 39-year-old female with frequent cystitis on chronic suppressive antibiotics is evaluated for worsening dyspnea. High resolution CT shows diffuse fibrosis. What antibiotic was this patient most likely on?
A. Nitrofurantoin
B. Cephalexin
C. TMX-sulfamethoxazole
D. Fosfomycin
A. Nitrofurantoin
Nitrofurantoin is known to cause pulmonary toxicity.

## Slide 43
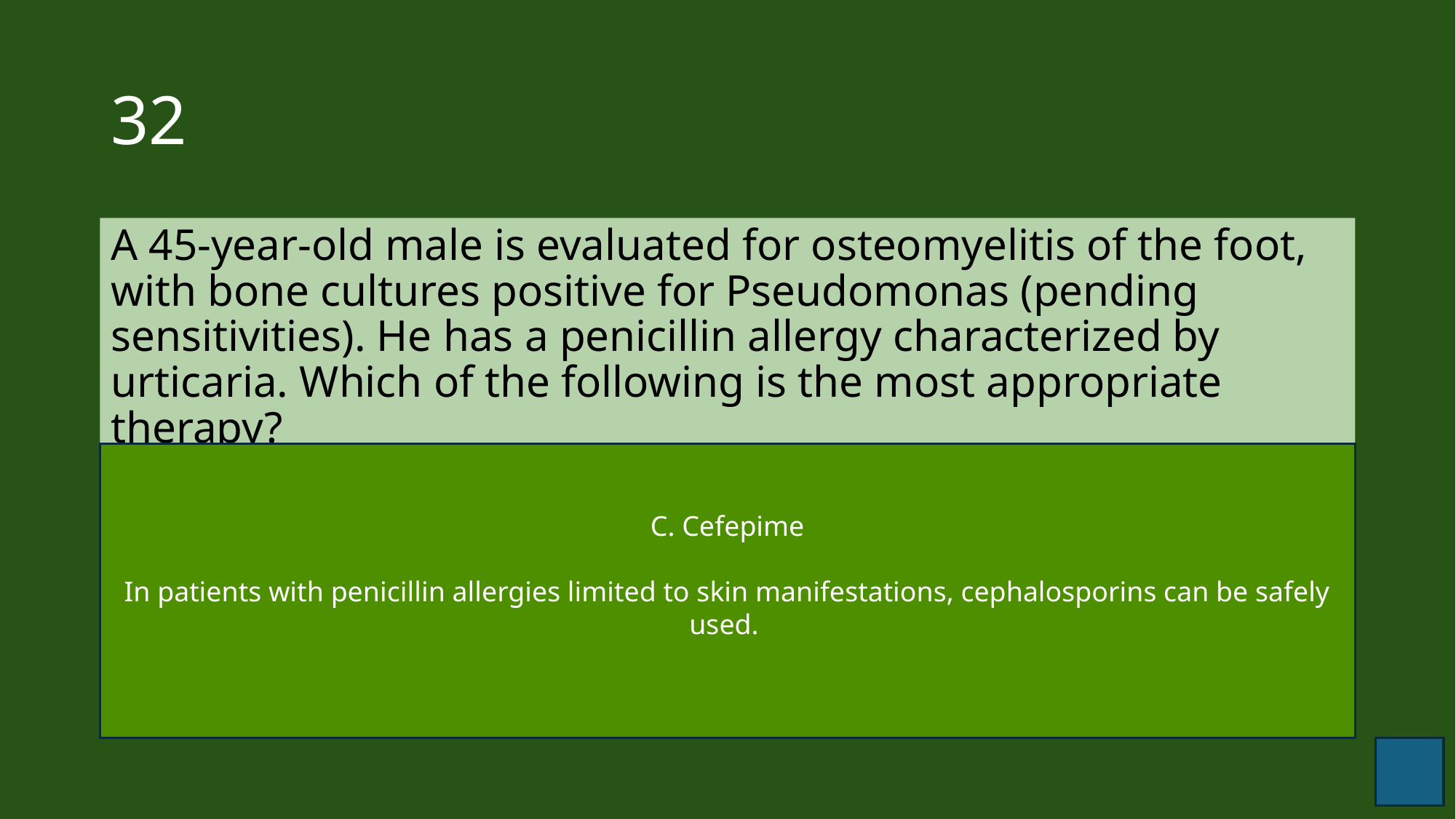

# 32
A 45-year-old male is evaluated for osteomyelitis of the foot, with bone cultures positive for Pseudomonas (pending sensitivities). He has a penicillin allergy characterized by urticaria. Which of the following is the most appropriate therapy?
Piperacillin-tazobactam
Ertapenem
Cefepime
Ticarcillin
C. Cefepime
In patients with penicillin allergies limited to skin manifestations, cephalosporins can be safely used.

## Slide 44
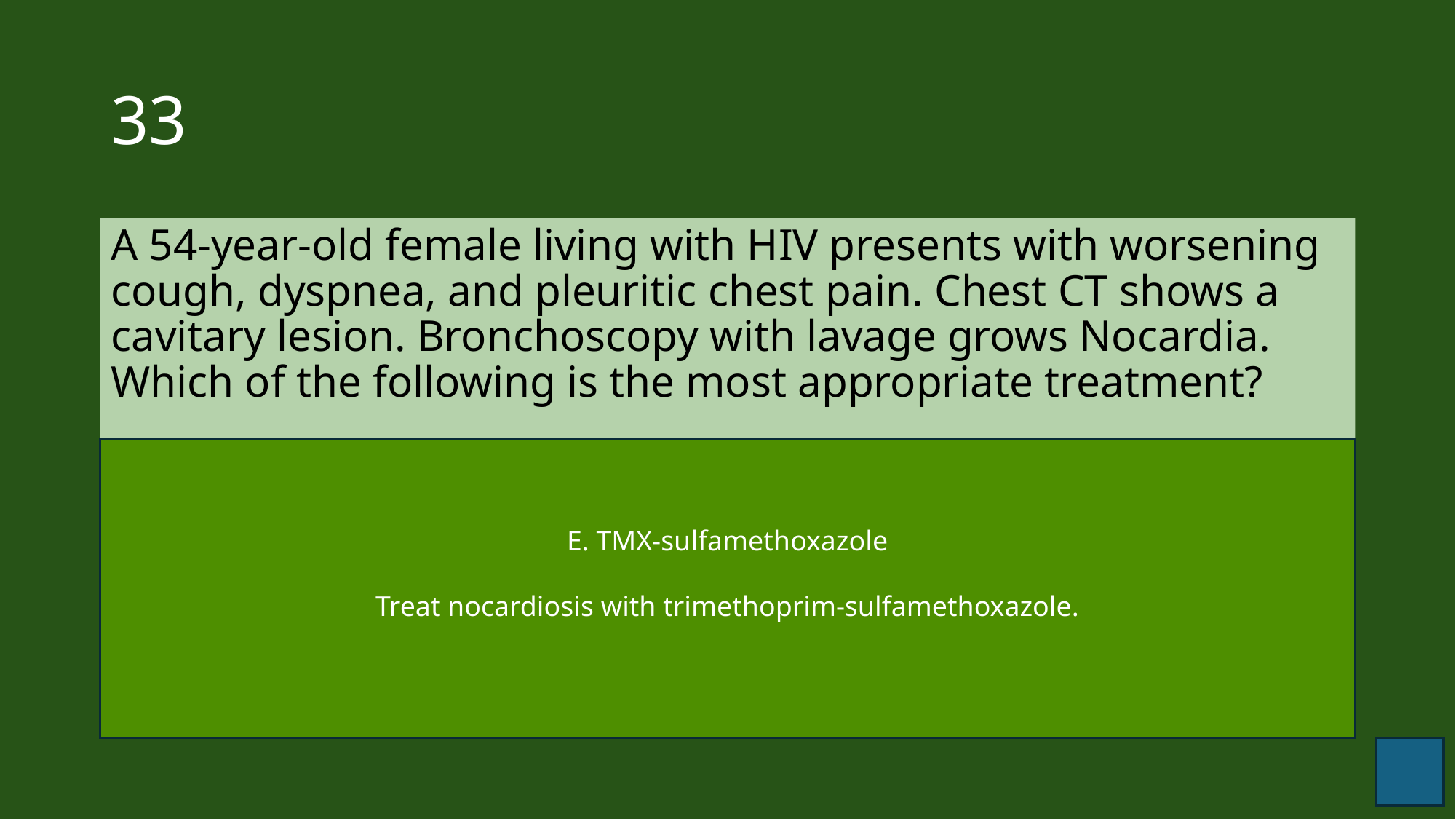

# 33
A 54-year-old female living with HIV presents with worsening cough, dyspnea, and pleuritic chest pain. Chest CT shows a cavitary lesion. Bronchoscopy with lavage grows Nocardia. Which of the following is the most appropriate treatment?
Amphotericin B
Voriconazole
Metronidazole
Flucytosine
TMX-sulfamethoxazole
E. TMX-sulfamethoxazole
Treat nocardiosis with trimethoprim-sulfamethoxazole.

## Slide 45
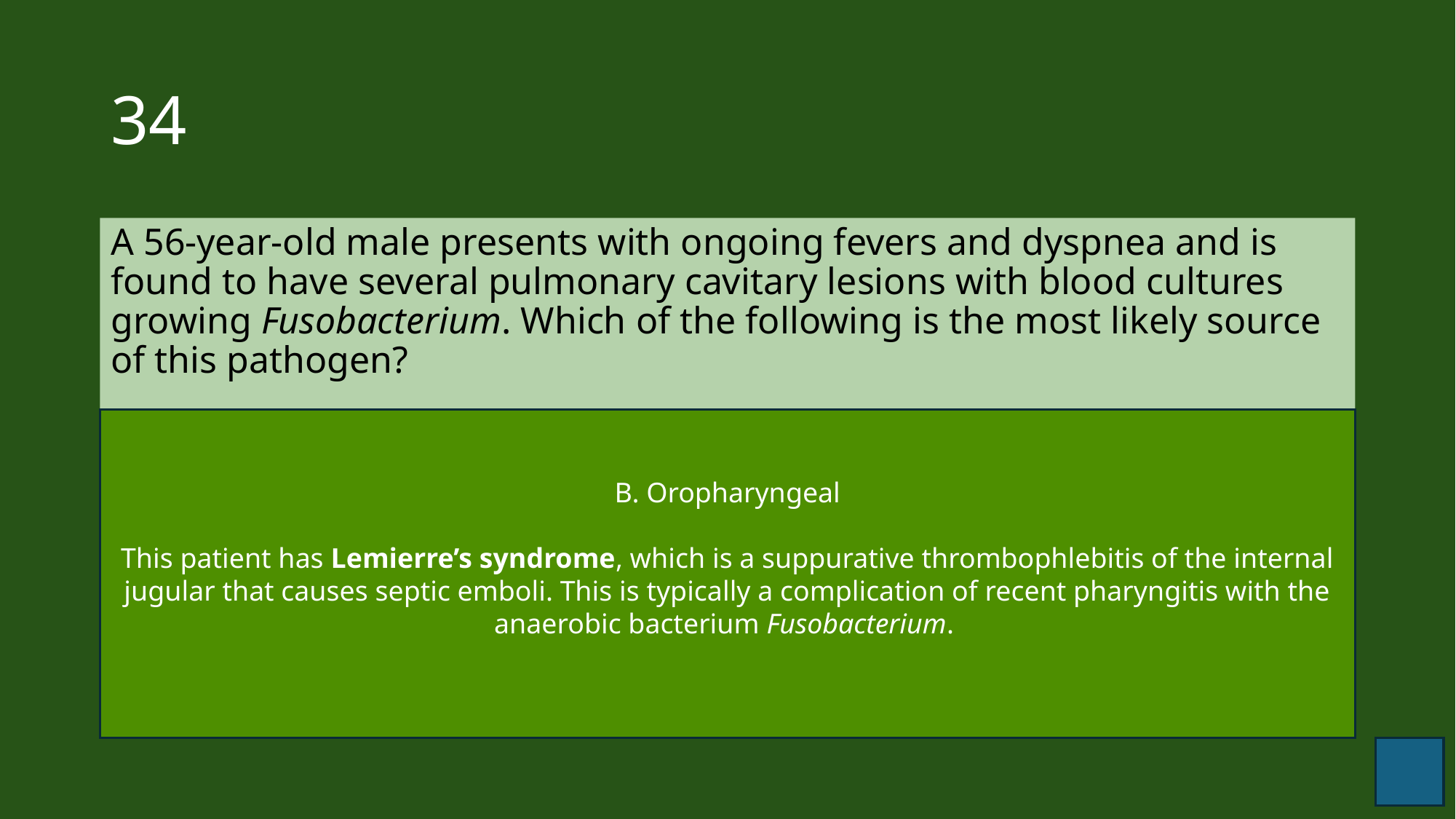

# 34
A 56-year-old male presents with ongoing fevers and dyspnea and is found to have several pulmonary cavitary lesions with blood cultures growing Fusobacterium. Which of the following is the most likely source of this pathogen?
IV drug use
Oropharyngeal
Colonic entry in setting of metastasis
Airborne
B. Oropharyngeal
This patient has Lemierre’s syndrome, which is a suppurative thrombophlebitis of the internal jugular that causes septic emboli. This is typically a complication of recent pharyngitis with the anaerobic bacterium Fusobacterium.

## Slide 46
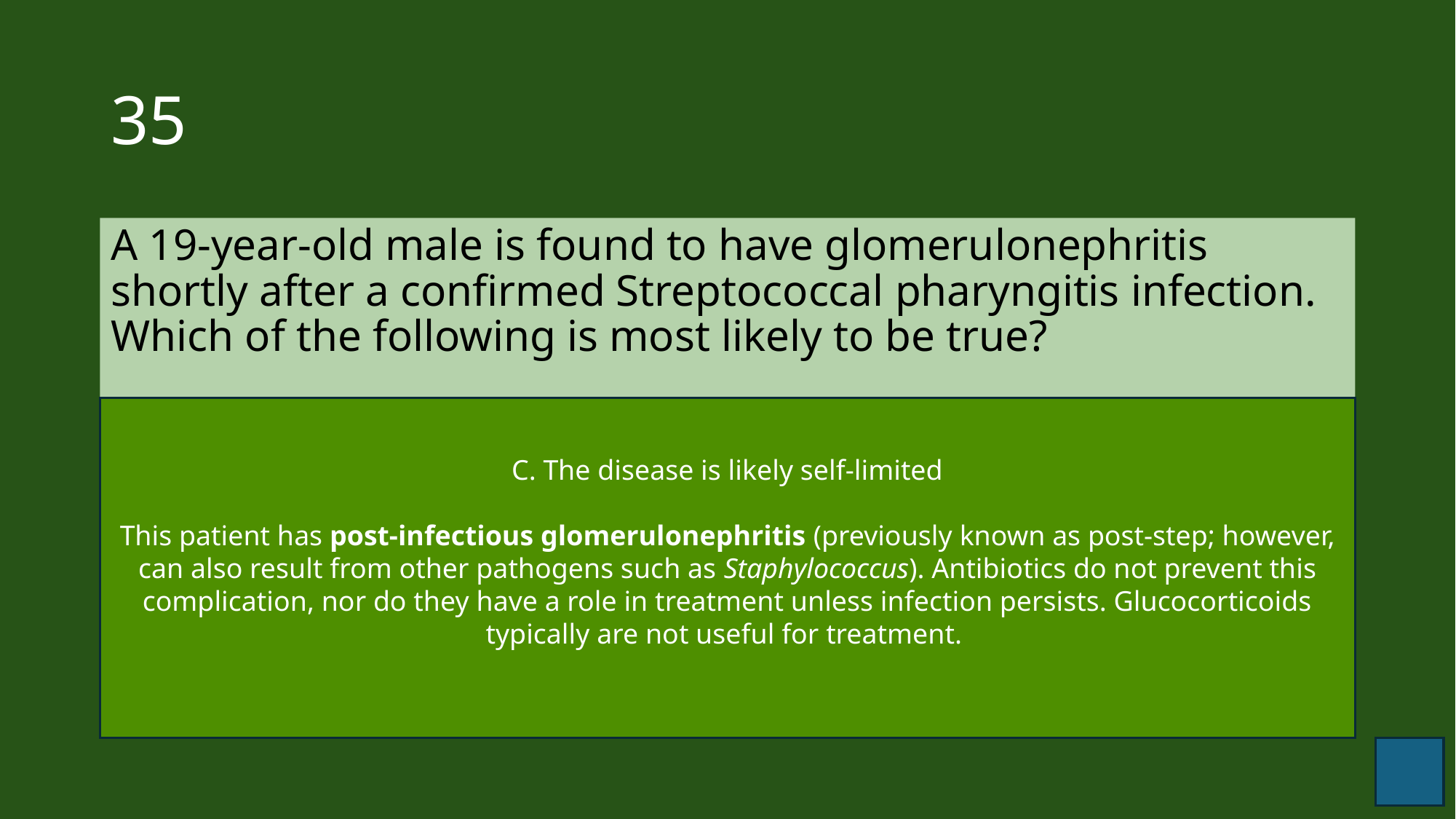

# 35
A 19-year-old male is found to have glomerulonephritis shortly after a confirmed Streptococcal pharyngitis infection. Which of the following is most likely to be true?
Amoxicillin would have prevented this complication
Antibiotics should be initiated regardless of whether pharyngitis is still present
This disease is likely self-limited
The patient should be started on glucocorticoids
C. The disease is likely self-limited
This patient has post-infectious glomerulonephritis (previously known as post-step; however, can also result from other pathogens such as Staphylococcus). Antibiotics do not prevent this complication, nor do they have a role in treatment unless infection persists. Glucocorticoids typically are not useful for treatment.

## Slide 47
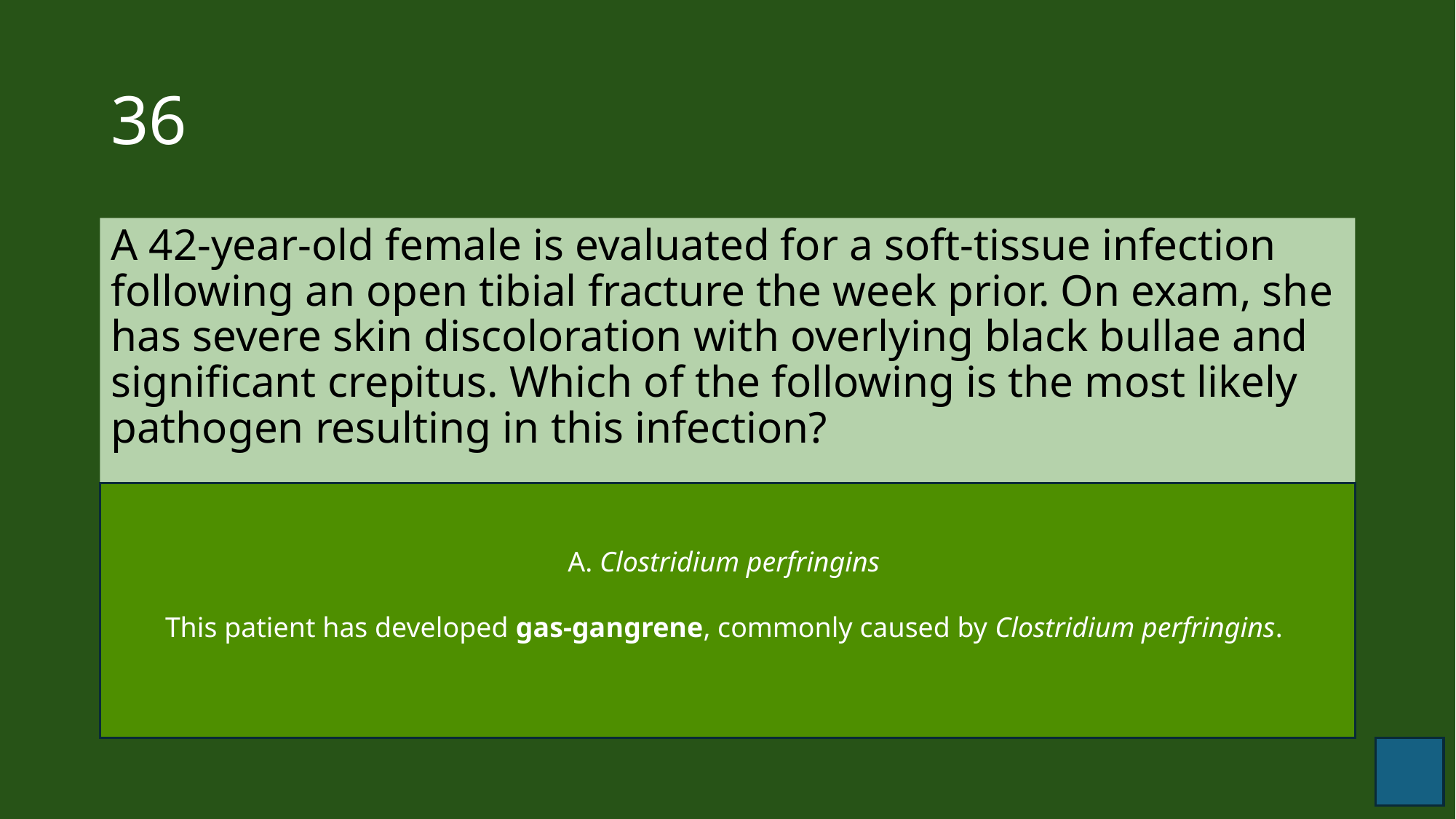

# 36
A 42-year-old female is evaluated for a soft-tissue infection following an open tibial fracture the week prior. On exam, she has severe skin discoloration with overlying black bullae and significant crepitus. Which of the following is the most likely pathogen resulting in this infection?
A. Clostridium perfringens
B. Clostridium tetani
C. Staphylococcus aureus
D. Pseudomonas aeruginosa
A. Clostridium perfringins
This patient has developed gas-gangrene, commonly caused by Clostridium perfringins.

## Slide 48
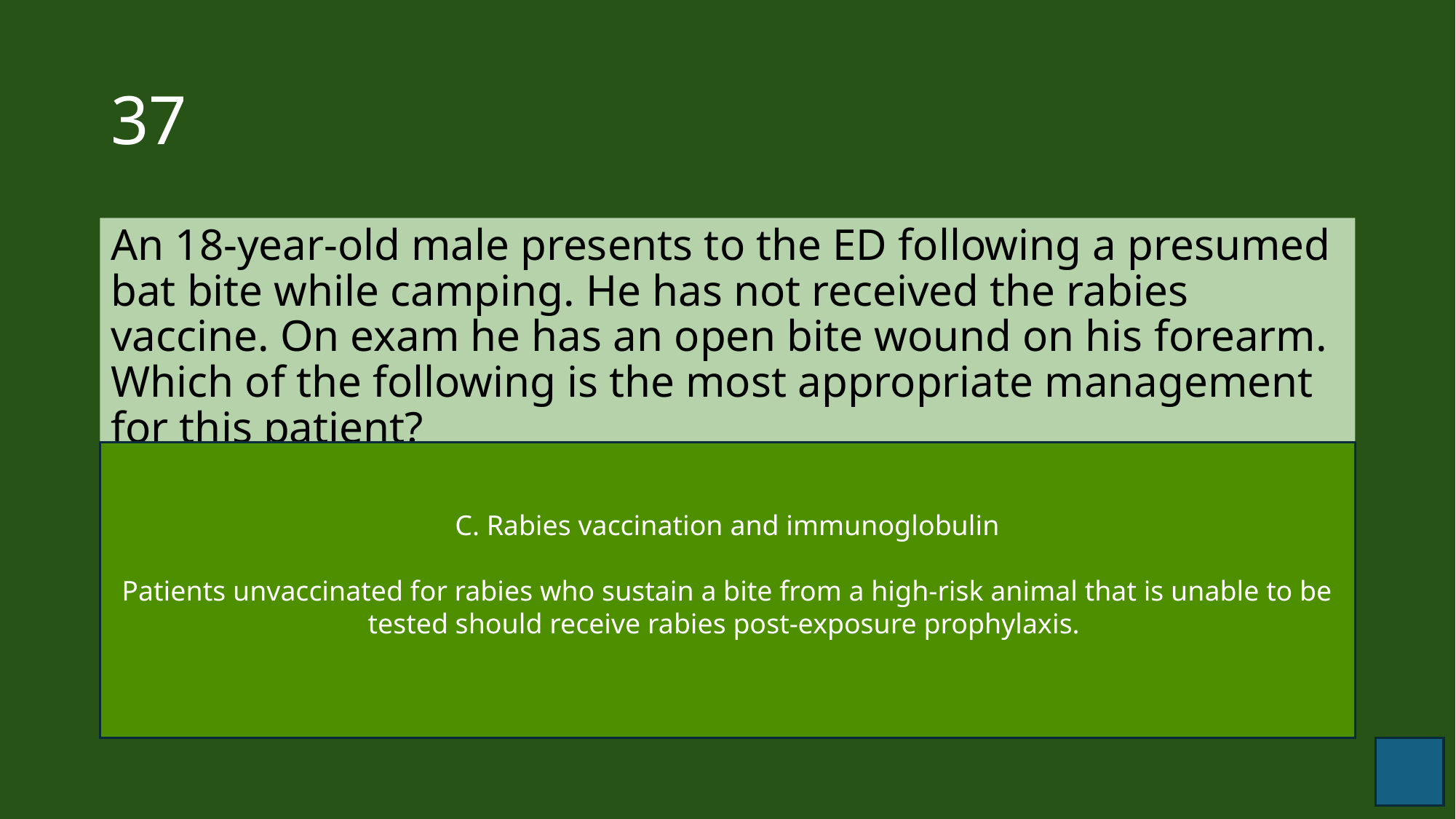

# 37
An 18-year-old male presents to the ED following a presumed bat bite while camping. He has not received the rabies vaccine. On exam he has an open bite wound on his forearm. Which of the following is the most appropriate management for this patient?
 Rabies vaccination alone
 Rabies immunoglobulin alone
 Rabies vaccination and immunoglobulin
 Cleaning and debridement; observe for neurological symptoms
C. Rabies vaccination and immunoglobulin
Patients unvaccinated for rabies who sustain a bite from a high-risk animal that is unable to be tested should receive rabies post-exposure prophylaxis.

## Slide 49
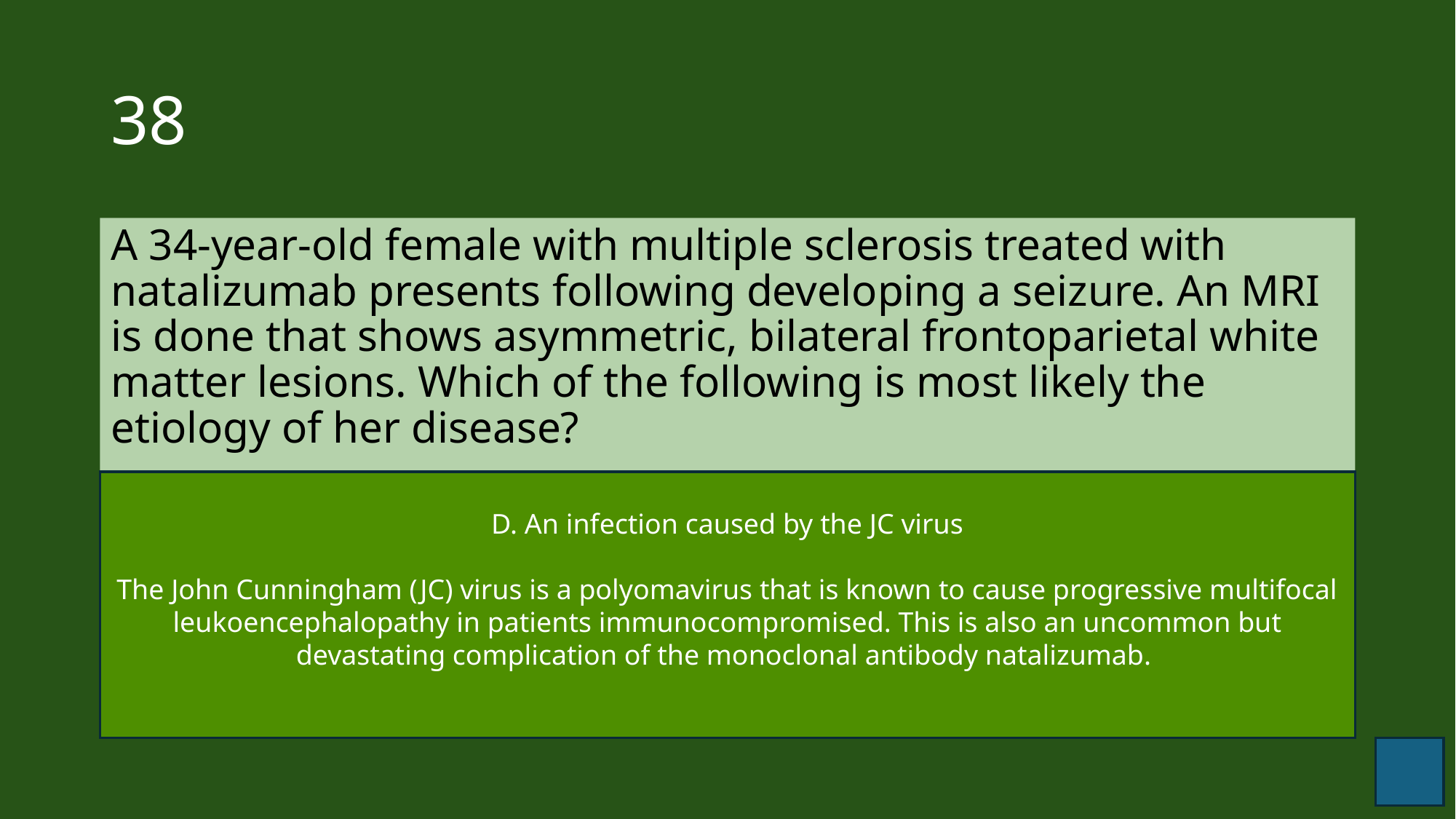

# 38
A 34-year-old female with multiple sclerosis treated with natalizumab presents following developing a seizure. An MRI is done that shows asymmetric, bilateral frontoparietal white matter lesions. Which of the following is most likely the etiology of her disease?
CNS lymphoma
 A multiple sclerosis flare
 An infection caused by the BK virus
 An infection caused by the JC virus
D. An infection caused by the JC virus
The John Cunningham (JC) virus is a polyomavirus that is known to cause progressive multifocal leukoencephalopathy in patients immunocompromised. This is also an uncommon but devastating complication of the monoclonal antibody natalizumab.

## Slide 50
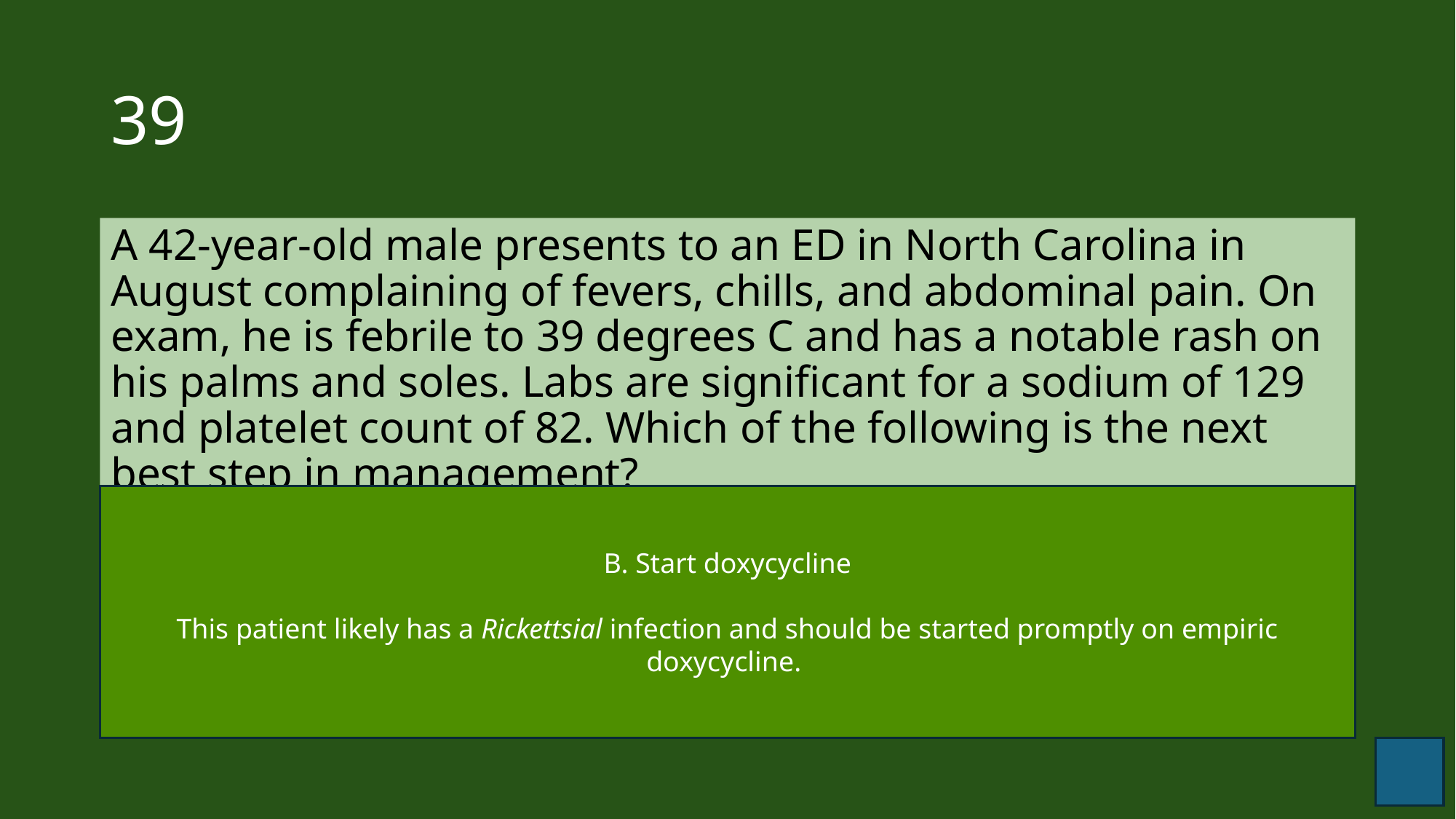

# 39
A 42-year-old male presents to an ED in North Carolina in August complaining of fevers, chills, and abdominal pain. On exam, he is febrile to 39 degrees C and has a notable rash on his palms and soles. Labs are significant for a sodium of 129 and platelet count of 82. Which of the following is the next best step in management?
Start amphotericin B
Start doxycycline
Start atovaquone and azithromycin
Start penicillin
B. Start doxycycline
This patient likely has a Rickettsial infection and should be started promptly on empiric doxycycline.

## Slide 51
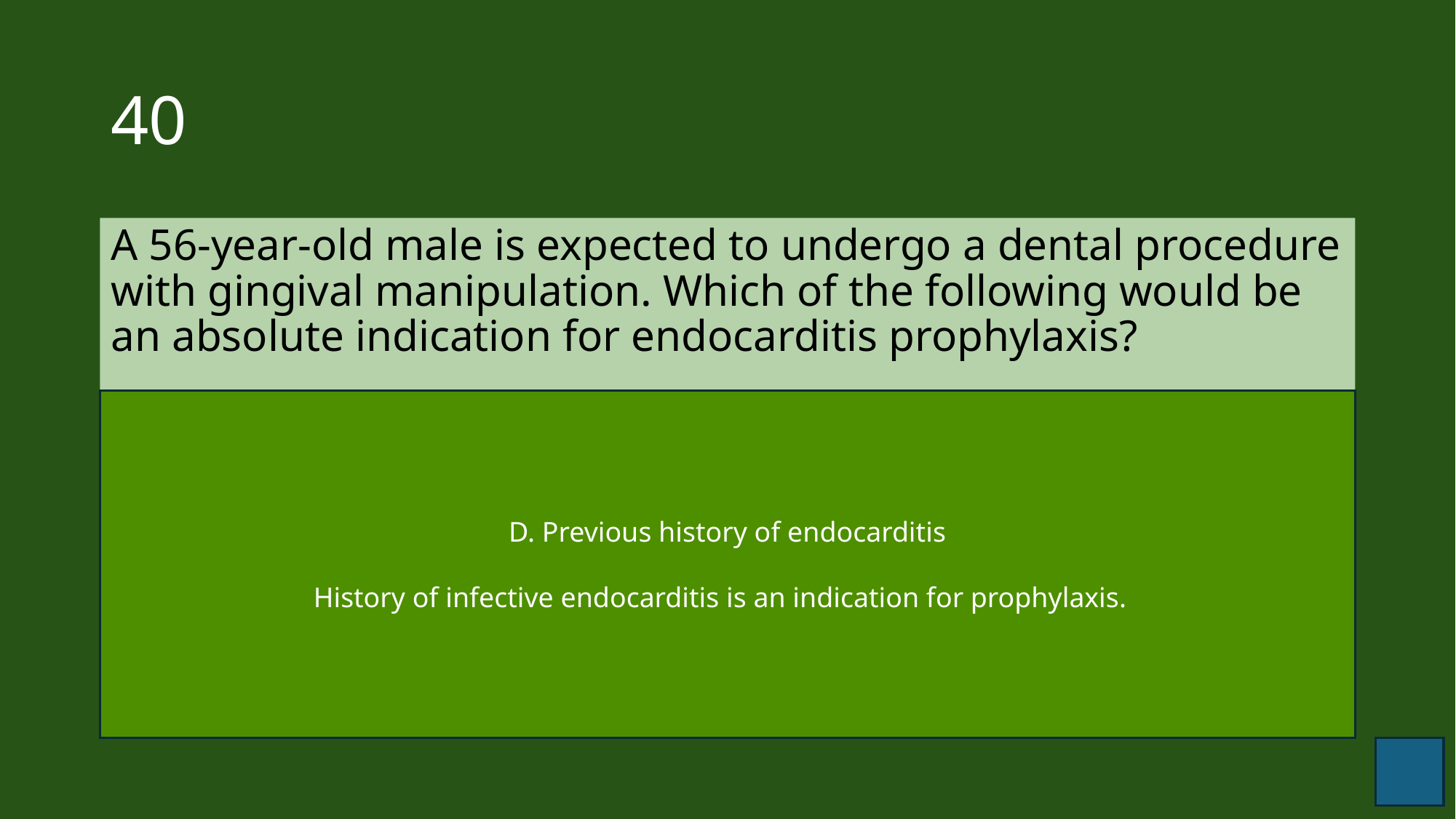

# 40
A 56-year-old male is expected to undergo a dental procedure with gingival manipulation. Which of the following would be an absolute indication for endocarditis prophylaxis?
Transcatheter atrial septal defect closure as a child
 Severe aortic stenosis
 Cardiac conduction delay
 Previous history of infectious endocarditis
D. Previous history of endocarditis
History of infective endocarditis is an indication for prophylaxis.

## Slide 52
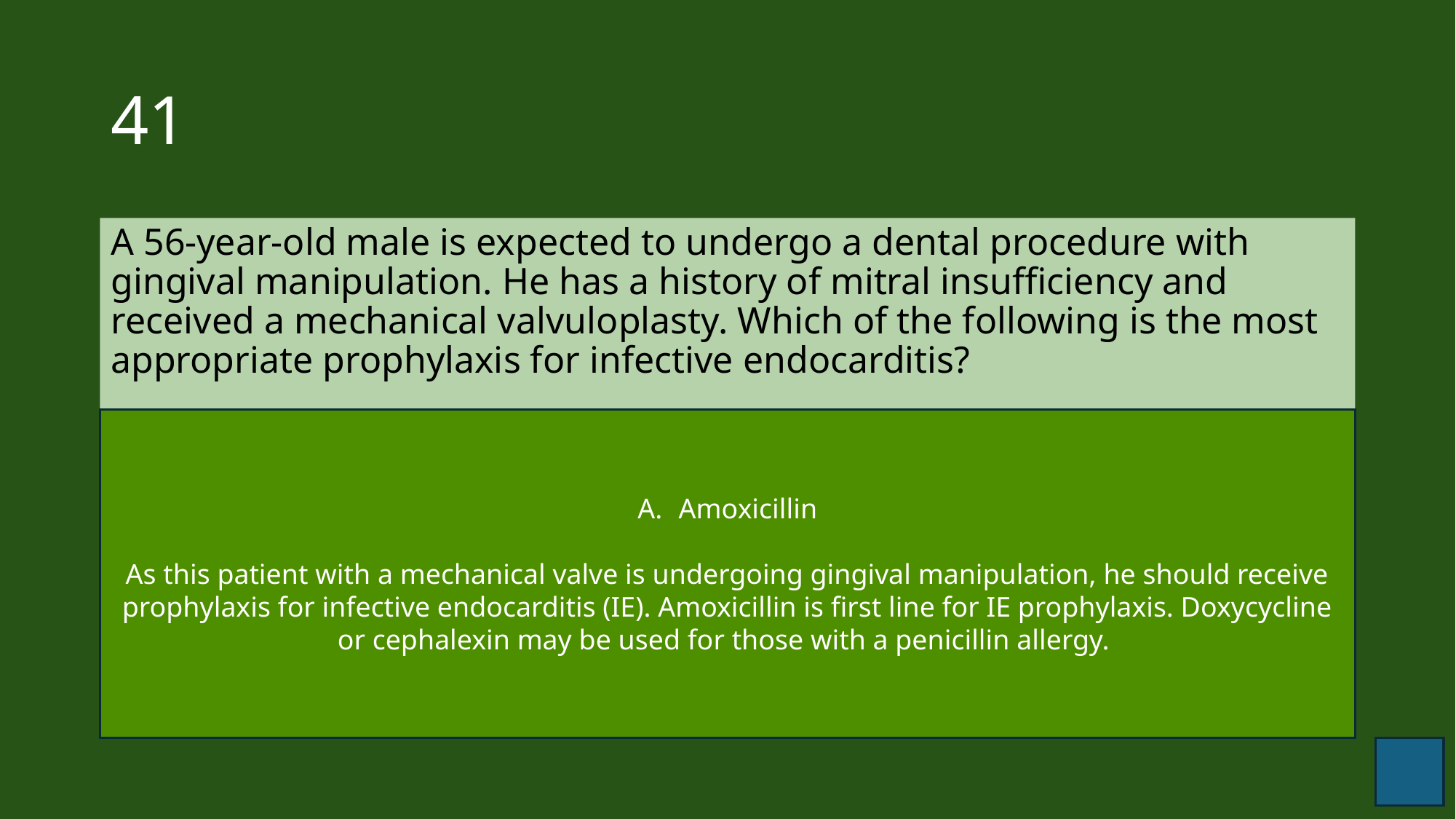

# 41
A 56-year-old male is expected to undergo a dental procedure with gingival manipulation. He has a history of mitral insufficiency and received a mechanical valvuloplasty. Which of the following is the most appropriate prophylaxis for infective endocarditis?
Amoxicillin
TMX-sulfamethoxazole
Linezolid
No prophylaxis indicated
Amoxicillin
As this patient with a mechanical valve is undergoing gingival manipulation, he should receive prophylaxis for infective endocarditis (IE). Amoxicillin is first line for IE prophylaxis. Doxycycline or cephalexin may be used for those with a penicillin allergy.

## Slide 53
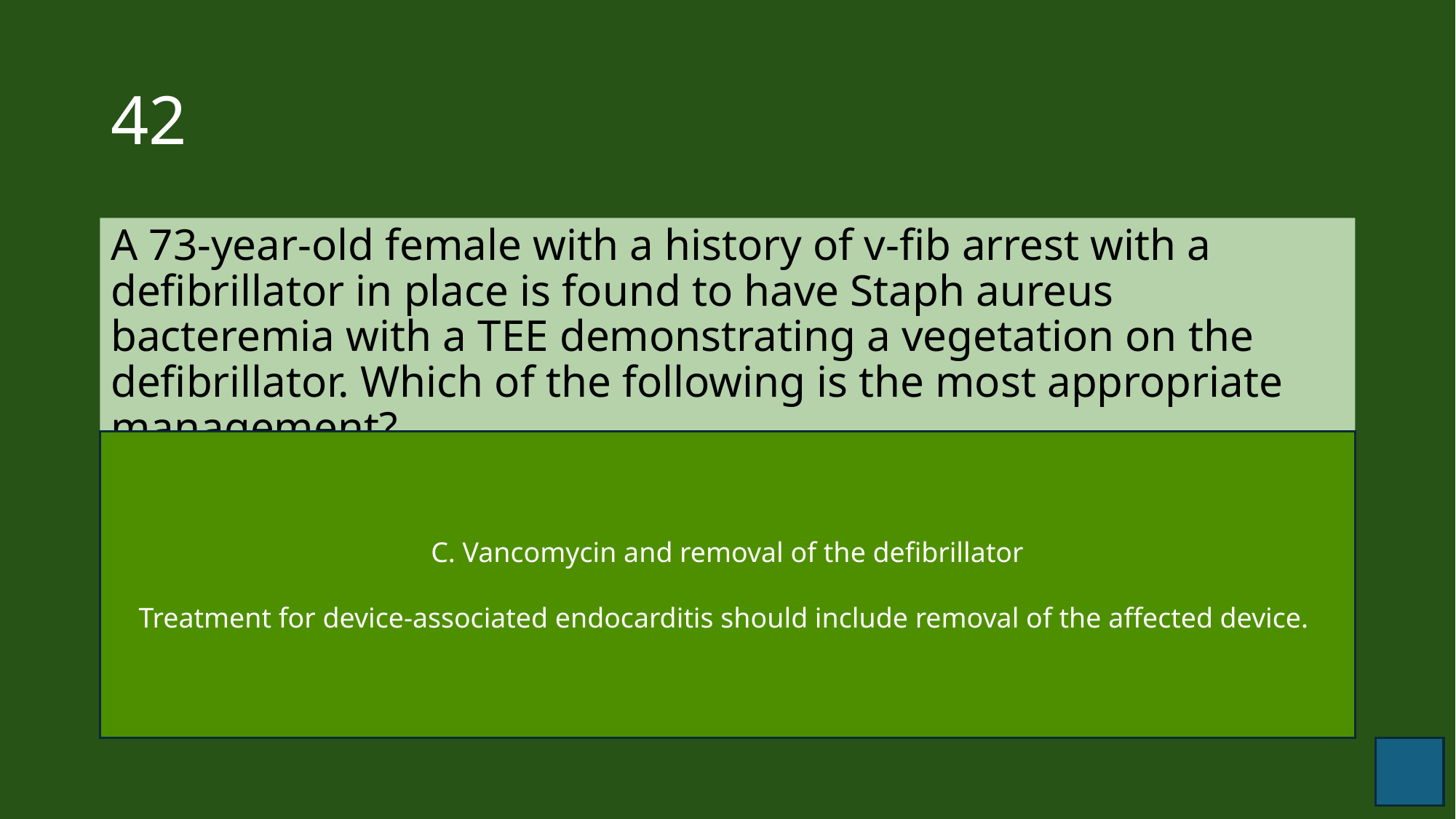

# 42
A 73-year-old female with a history of v-fib arrest with a defibrillator in place is found to have Staph aureus bacteremia with a TEE demonstrating a vegetation on the defibrillator. Which of the following is the most appropriate management?
 Combination of vancomycin and rifampin
 Combination of daptomycin and ceftaroline
 Vancomycin and removal of the defibrillator
 Vancomycin alone
C. Vancomycin and removal of the defibrillator
Treatment for device-associated endocarditis should include removal of the affected device.

## Slide 54
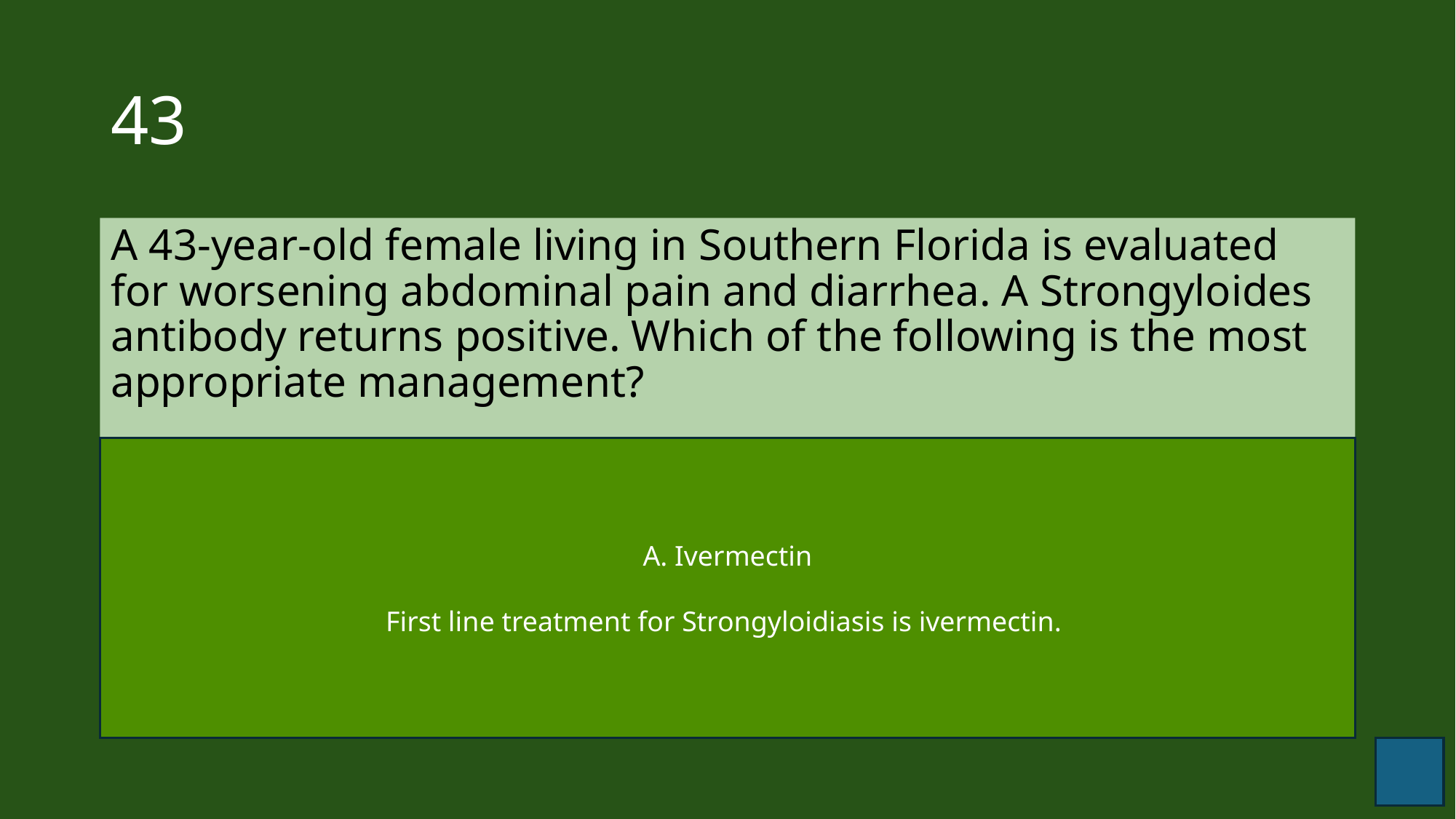

# 43
A 43-year-old female living in Southern Florida is evaluated for worsening abdominal pain and diarrhea. A Strongyloides antibody returns positive. Which of the following is the most appropriate management?
A. Ivermectin
B. Metronidazole
C. Nitazoxanide
D. Pyrimethamine
A. Ivermectin
First line treatment for Strongyloidiasis is ivermectin.

## Slide 55
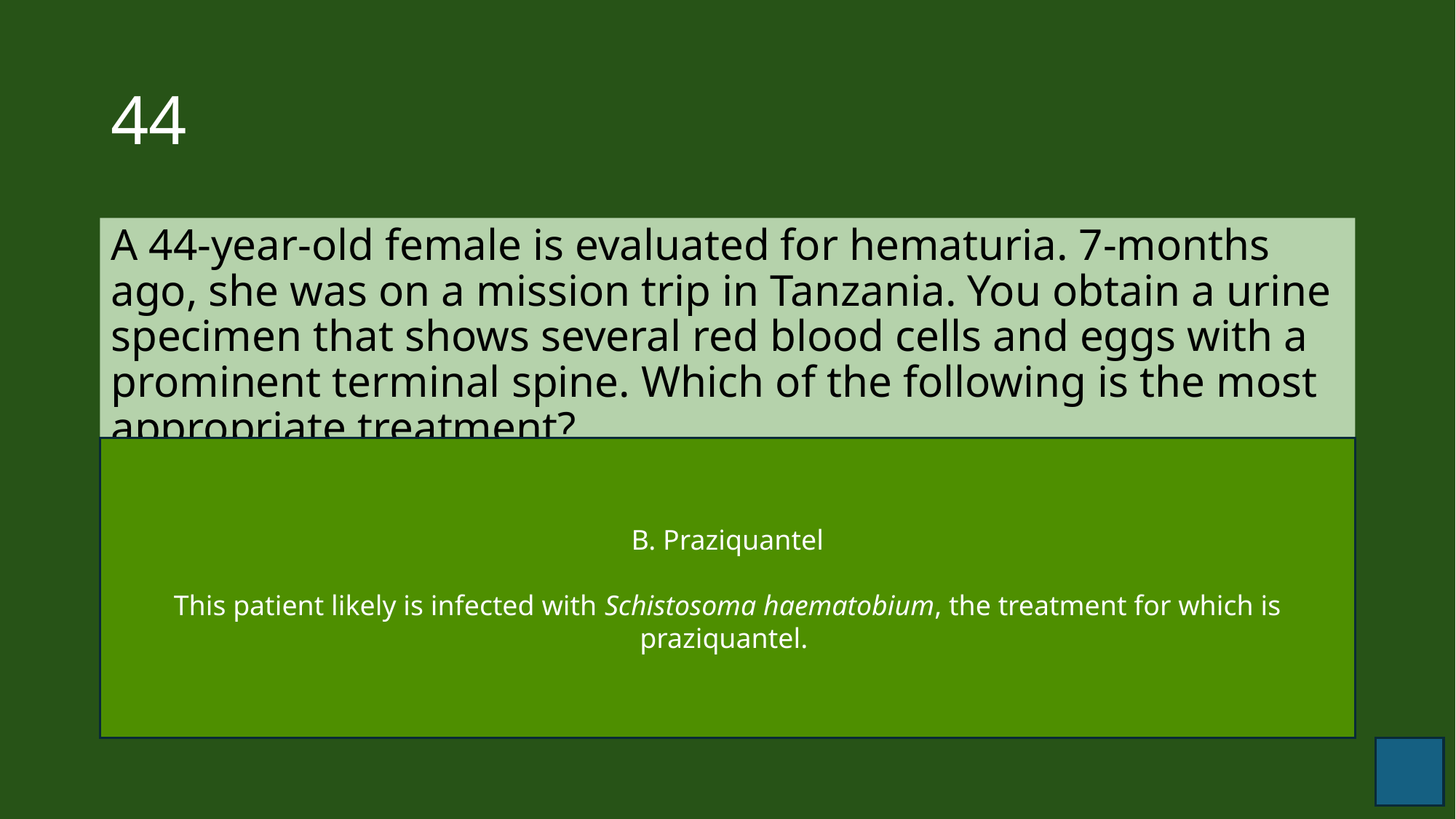

# 44
A 44-year-old female is evaluated for hematuria. 7-months ago, she was on a mission trip in Tanzania. You obtain a urine specimen that shows several red blood cells and eggs with a prominent terminal spine. Which of the following is the most appropriate treatment?
A. Metronidazole
B. Praziquantel
C. Ivermectin
D. Nitazoxanide
B. Praziquantel
This patient likely is infected with Schistosoma haematobium, the treatment for which is praziquantel.

## Slide 56
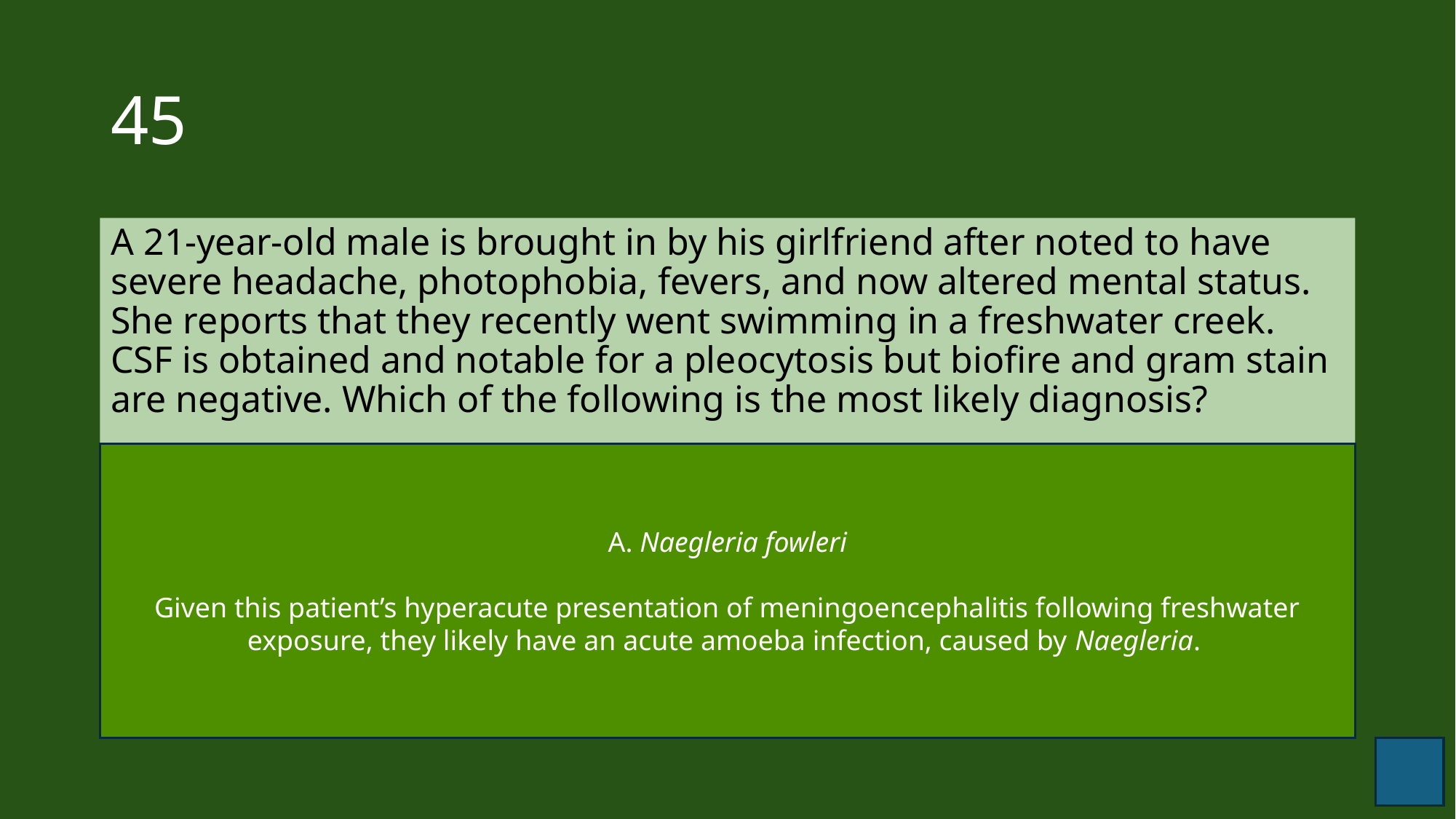

# 45
A 21-year-old male is brought in by his girlfriend after noted to have severe headache, photophobia, fevers, and now altered mental status. She reports that they recently went swimming in a freshwater creek. CSF is obtained and notable for a pleocytosis but biofire and gram stain are negative. Which of the following is the most likely diagnosis?
Naegleria fowleri
Taenia solium
Neurocysticercosis
Nocardiosis
A. Naegleria fowleri
Given this patient’s hyperacute presentation of meningoencephalitis following freshwater exposure, they likely have an acute amoeba infection, caused by Naegleria.

## Slide 57
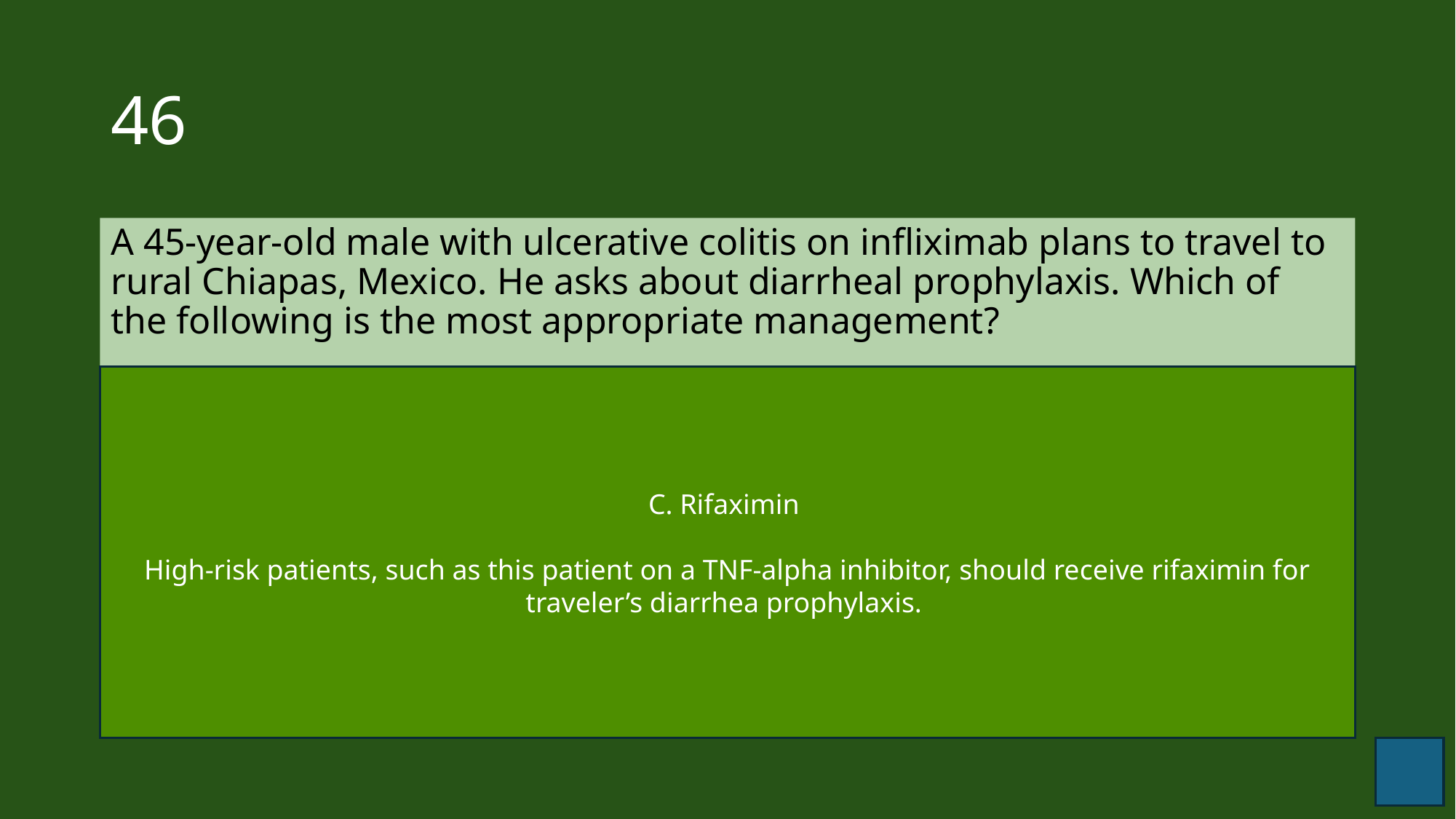

# 46
A 45-year-old male with ulcerative colitis on infliximab plans to travel to rural Chiapas, Mexico. He asks about diarrheal prophylaxis. Which of the following is the most appropriate management?
A. Lactobacillus
B. Azithromycin
C. Rifaximin
Loperamide
No prophylaxis indicated
C. Rifaximin
High-risk patients, such as this patient on a TNF-alpha inhibitor, should receive rifaximin for traveler’s diarrhea prophylaxis.

## Slide 58
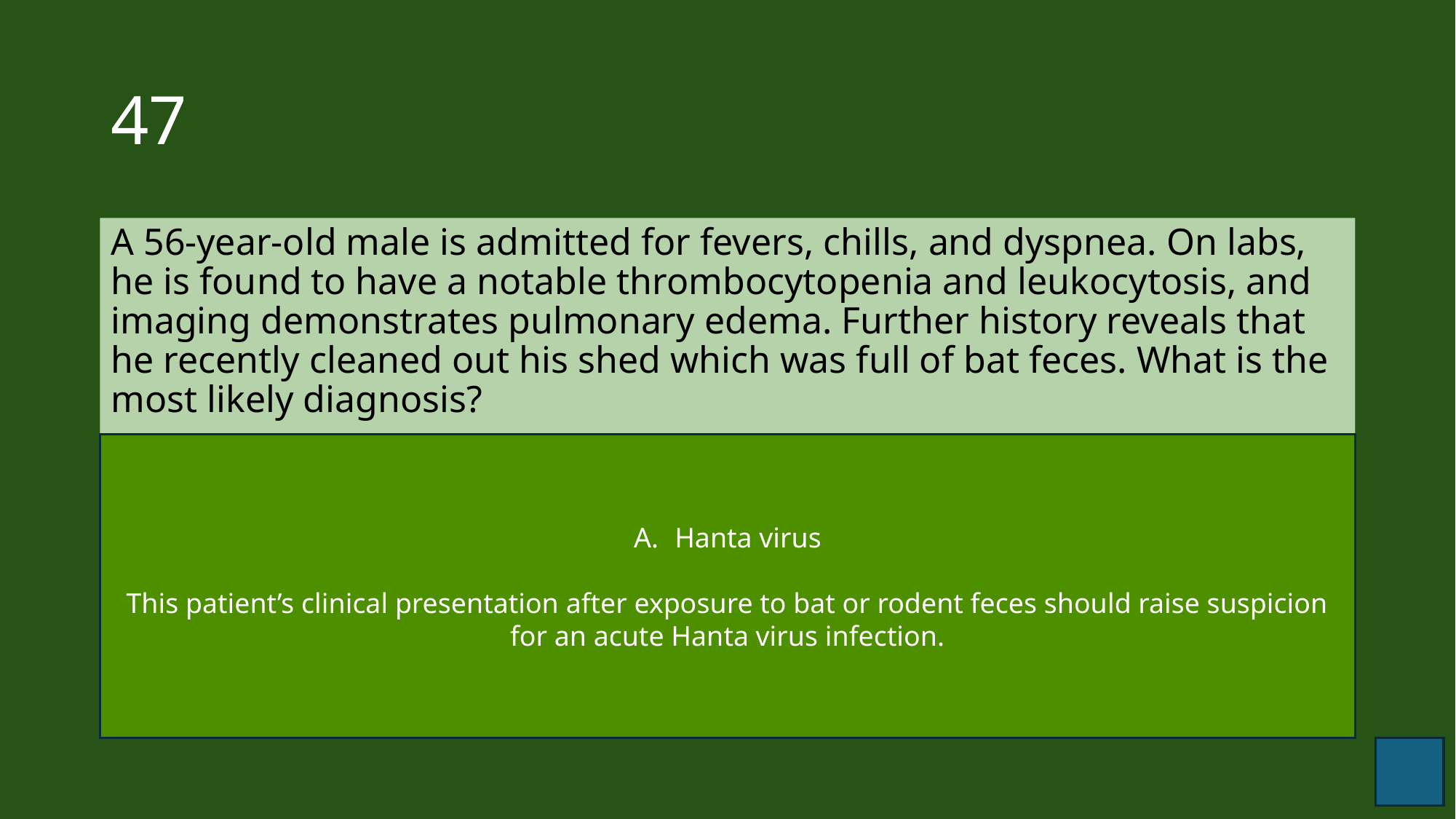

# 47
A 56-year-old male is admitted for fevers, chills, and dyspnea. On labs, he is found to have a notable thrombocytopenia and leukocytosis, and imaging demonstrates pulmonary edema. Further history reveals that he recently cleaned out his shed which was full of bat feces. What is the most likely diagnosis?
A. Hanta virus
B. Leptospirosis
C. Aeromonas
D. Aspergillus
Hanta virus
This patient’s clinical presentation after exposure to bat or rodent feces should raise suspicion for an acute Hanta virus infection.

## Slide 59
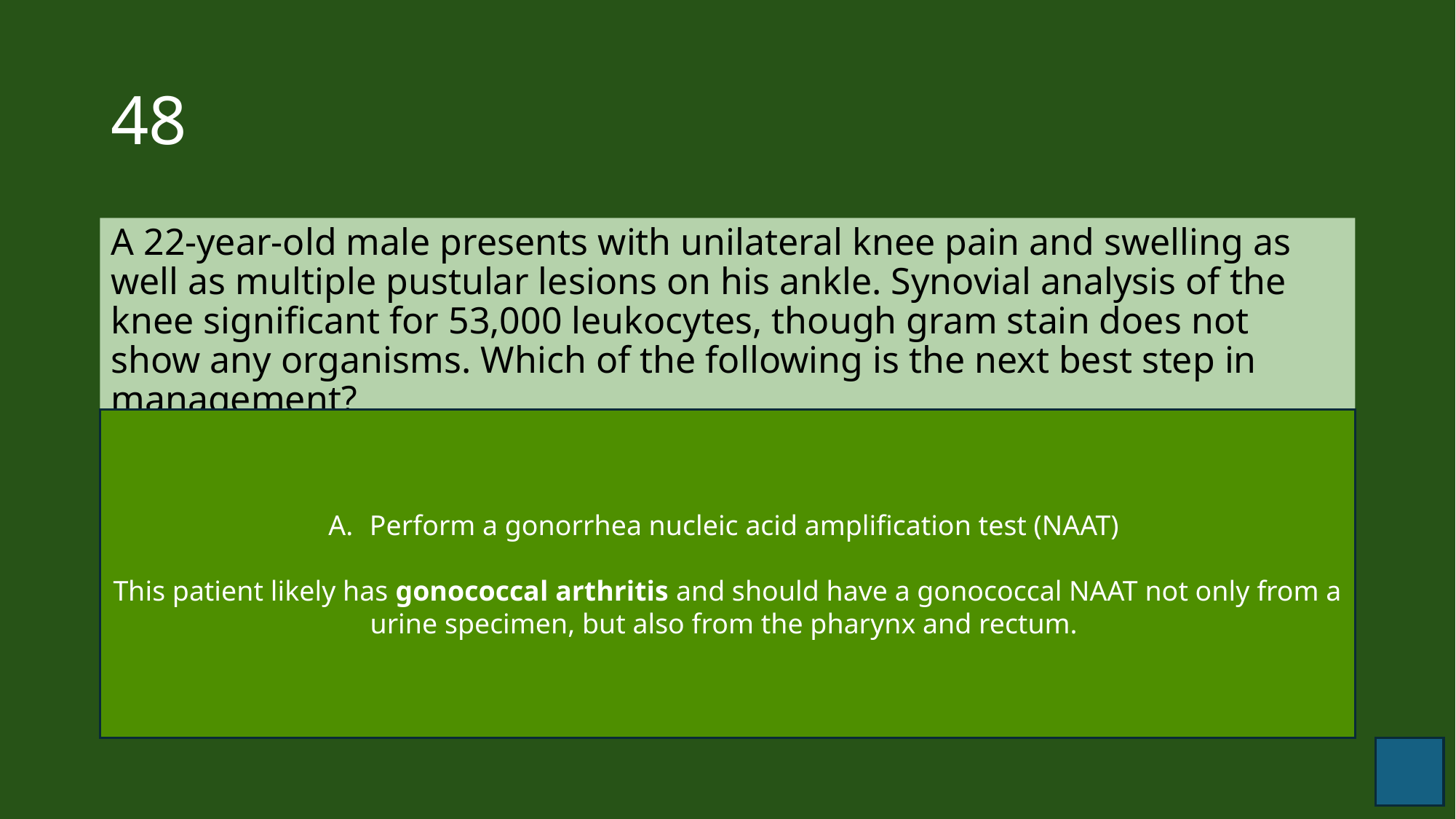

# 48
A 22-year-old male presents with unilateral knee pain and swelling as well as multiple pustular lesions on his ankle. Synovial analysis of the knee significant for 53,000 leukocytes, though gram stain does not show any organisms. Which of the following is the next best step in management?
Perform a gonorrhea nucleic acid amplification test
Start high dose NSAIDS
Obtain HLA-B27
Obtain echocardiogram
Perform a gonorrhea nucleic acid amplification test (NAAT)
This patient likely has gonococcal arthritis and should have a gonococcal NAAT not only from a urine specimen, but also from the pharynx and rectum.

## Slide 60
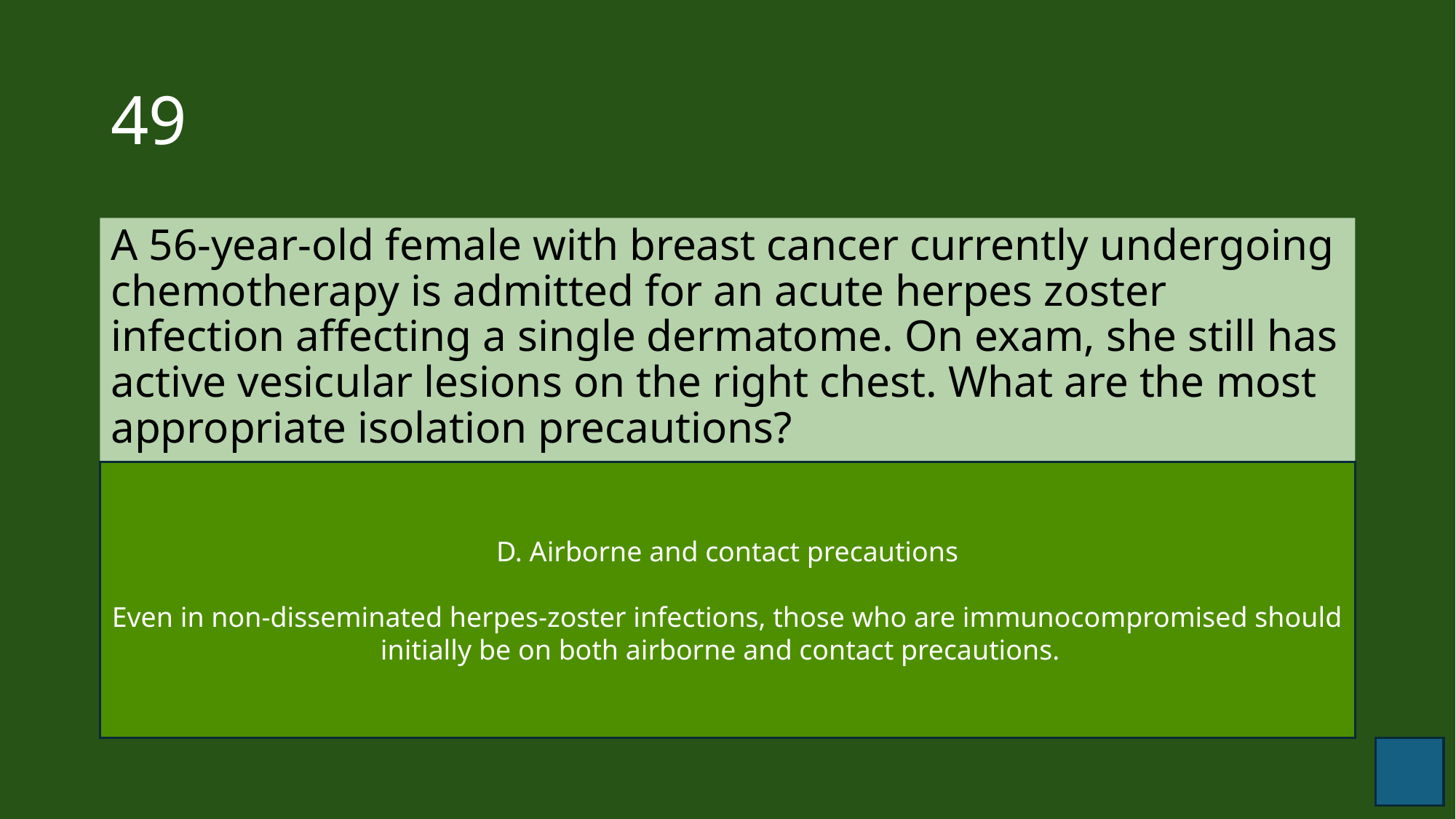

# 49
A 56-year-old female with breast cancer currently undergoing chemotherapy is admitted for an acute herpes zoster infection affecting a single dermatome. On exam, she still has active vesicular lesions on the right chest. What are the most appropriate isolation precautions?
 No isolation
 Contact precautions
 Droplet precautions
 Airborne and contact precautions
D. Airborne and contact precautions
Even in non-disseminated herpes-zoster infections, those who are immunocompromised should initially be on both airborne and contact precautions.

## Slide 61
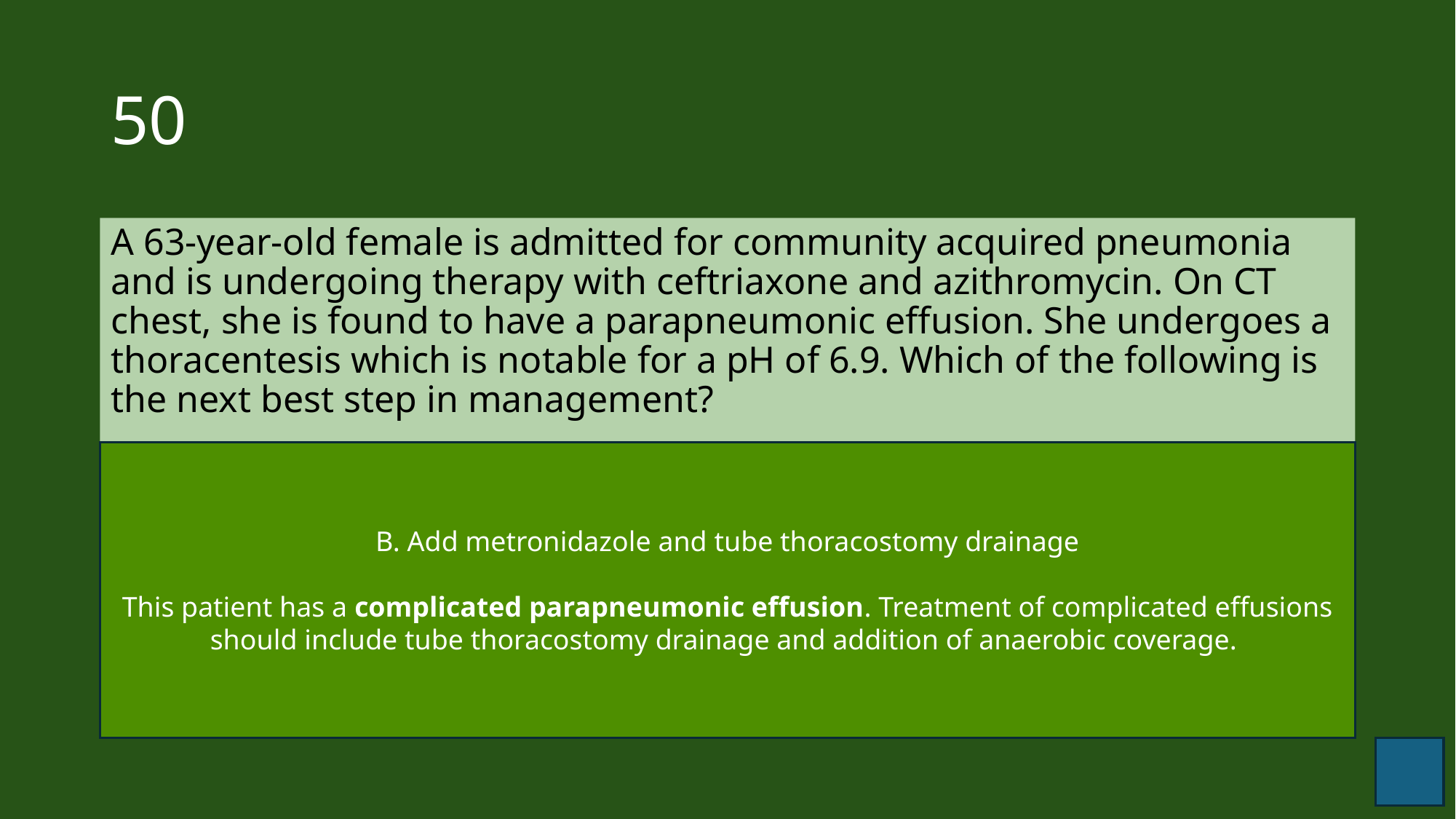

# 50
A 63-year-old female is admitted for community acquired pneumonia and is undergoing therapy with ceftriaxone and azithromycin. On CT chest, she is found to have a parapneumonic effusion. She undergoes a thoracentesis which is notable for a pH of 6.9. Which of the following is the next best step in management?
A. Add metronidazole
B. Add metronidazole and tube thoracostomy drainage
C. Continue current antibiotics and perform thoracostomy drainage
D. Video-assisted thoracic surgery pleurodesis
B. Add metronidazole and tube thoracostomy drainage
This patient has a complicated parapneumonic effusion. Treatment of complicated effusions should include tube thoracostomy drainage and addition of anaerobic coverage.
