# Supplementary material for: Pathogen Pursuit: A Gamified Format to Learn Infectious Diseases and Antimicrobial Stewardship for Medical Residents
Source: MedEdPORTAL. 2025 Dec 16;21:11565. doi: 10.15766/mep_2374-8265.11565 (PMC12705857; doi:10.15766/mep_2374-8265.11565)
Supplement: Supplementary file 1 — Educational Objectives by Quesitons.docxGame Instructions.docxPathogen Game Cards.pdfAntimicrobial Game Cards.pdfGame Board Slide Show.pptxKey.pdfPostgame Survey.docxPre- and Posttest.docx [file mep_2374-8265.11565-s001.zip › F. Key.pdf]

Answer key

Pathogen												
Aspiration-pneumonia with abscess	C	K + Y	K + M	AC	S	X						
Candida auris	Z	D										
CAP (outpatient, no comorbidities)	Q	B	(no longer accept F due to emerging resistance)									
Chlamydia	Q	F										
Community aquired pneumonia (inpatient)	K + (F or Q)	X + (F or Q)	C + (F or Q)	I + (F or Q)								
Enterobacter cloacae	I	X	S									
Enterococcus faecalis	B	C	AC	X	S	AH	O	W	AE			
Enterococcus faecium	X	S	AH	O	W	AE						
Enterococcus faecium (pan resistant)	AE											
ESBL E. coli Pyelonephritis	X	S	AA									
Esophageal candidiasis	T	Z	AI	D								
Genital herpes flare	A	AG										
Gonorrhea	K											
Hepatitis C	AD											
Herpes-zoster (disseminated)	A											
HIV	R + G	R + P	P + V									
Invasive aspergillus	AI	D										
Lyme disease	Q	K										
Malaria	E											
Meningitis (23 year old)	K + AH	I + AH										
Meningitis (59 year old)	K + AH + B	K + AH + B										
MRSA bacteremia	AH	O										
MRSA osteomyelitis	AH	O	W									
MRSA Pneumonia	AH	W										
MSSA bacteremia	H	AH	O	K								
Mucormycosis	D											
Nagleria fowleri	D											
Neuroborelliosis	K											
Neurosyphillis	AB											
Non-purulent cellulitis	H	C	AC	S	Y							
Pneumocystis jerovici	AF											
Post-neurosurgical meningitis	I + AH	X + AH										
Pseudomonas (R to pip-tazo and cefepime)	X	J										
Pseudomonas (R to pip-tazo, cefepime, and car	J											
Pseudomonas pneumonia	AC	I	X	J	L							
Purulent cellulitis	M	Q	AF	AH	W	O						
Rocky mountain spotted fever	Q											
Simple cystitis	AF	AA	B	AB	H	I	J	K	L	S	X	AC
Stenotrophomonas	AF	L										
Strep pharyngitis	B	C	AB									
Strongyliodies	U											
Syphillis	AB	Q	K									
Tinea corporis	N	T	D									

	Antimicrobial
A	Acyclovir
B	Amoxicillin or Ampicillin
C	Ampicillin-sulbactam
D	Amphotericin B
E	Artesunate
F	Azithromycin
G	Bictegravir
H	Cefazolin
I	Cefepime
J	Ceftazidime-Avibactam
K	Ceftriaxone
L	Ciprofloxacin
M	Clindamycin
N	Clotrimazole
O	Daptomycin
P	Dolutegravir
Q	Doxycycline
R	Emtricitabine-tenofovir
S	Ertapenem
T	Fluconazole
U	Ivermectin
V	Lamivudine
W	Linezolid
X	Meropenem
Y	Metronidazole
Z	Micafungin
AA	Nitrofurantoin
AB	Penicillin
AC	Piperacillin-tazobactam
AD	Sofasbuvir
AE	Tigecycline
AF	Trimethoprim-sulfamethaxazole
AG	Valcylovir
AH	Vancomycin
AI	Voriconazole
