# Supplementary material for: Pathogen Pursuit: A Gamified Format to Learn Infectious Diseases and Antimicrobial Stewardship for Medical Residents
Source: MedEdPORTAL. 2025 Dec 16;21:11565. doi: 10.15766/mep_2374-8265.11565 (PMC12705857; doi:10.15766/mep_2374-8265.11565)
Supplement: Supplementary file 1 — Educational Objectives by Quesitons.docxGame Instructions.docxPathogen Game Cards.pdfAntimicrobial Game Cards.pdfGame Board Slide Show.pptxKey.pdfPostgame Survey.docxPre- and Posttest.docx [file mep_2374-8265.11565-s001.zip › G. Postgame Survey.docx]

**Pathogen Pursuit: Post-game survey**

What post-graduate year are you in?

| PGY-1 |  |
| --- | --- |
| PGY-2 |  |
| PGY-3 |  |

To what extent do you agree or disagree with the following statements?

1. The instructions for this game were clear.

| Strongly disagree | Disagree | Neither Agree nor Disagree | Agree | Strongly Agree |
| --- | --- | --- | --- | --- |

1. The learning objectives of the game were met.

| Strongly disagree | Disagree | Neither Agree nor Disagree | Agree | Strongly Agree |
| --- | --- | --- | --- | --- |

1. The format of this game encouraged me to be engaged.

| Strongly disagree | Disagree | Neither Agree nor Disagree | Agree | Strongly Agree |
| --- | --- | --- | --- | --- |

1. The format of this game enhanced my ability to learn the topic.

| Strongly disagree | Disagree | Neither Agree nor Disagree | Agree | Strongly Agree |
| --- | --- | --- | --- | --- |

1. By the end of the game, I feel better prepared to manage real-life infectious disease cases.

| Strongly disagree | Disagree | Neither Agree nor Disagree | Agree | Strongly Agree |
| --- | --- | --- | --- | --- |

1. By the end of the game, I feel better prepared to answer ABIM style questions on infectious diseases.

| Strongly disagree | Disagree | Neither Agree nor Disagree | Agree | Strongly Agree |
| --- | --- | --- | --- | --- |

1. The content in this game was appropriate for my level of training.

| Strongly disagree | Disagree | Neither Agree nor Disagree | Agree | Strongly Agree |
| --- | --- | --- | --- | --- |

1. I would like similar game-based sessions in our curriculum.

| Strongly disagree | Disagree | Neither Agree nor Disagree | Agree | Strongly Agree |
| --- | --- | --- | --- | --- |

1. During the session I learnt about antimicrobial stewardship

| Strongly disagree | Disagree | Neither Agree nor Disagree | Agree | Strongly Agree |
| --- | --- | --- | --- | --- |

1. During the session I learnt how to best treat various pathogens

| Strongly disagree | Disagree | Neither Agree nor Disagree | Agree | Strongly Agree |
| --- | --- | --- | --- | --- |

1. The gamified session was an appropriate way to learn the delivered material

| Strongly disagree | Disagree | Neither Agree nor Disagree | Agree | Strongly Agree |
| --- | --- | --- | --- | --- |

1. Comments:

Appendix G: Post-game survey
