# Supplementary material for: Pathogen Pursuit: A Gamified Format to Learn Infectious Diseases and Antimicrobial Stewardship for Medical Residents
Source: MedEdPORTAL. 2025 Dec 16;21:11565. doi: 10.15766/mep_2374-8265.11565 (PMC12705857; doi:10.15766/mep_2374-8265.11565)
Supplement: Supplementary file 1 — Educational Objectives by Quesitons.docxGame Instructions.docxPathogen Game Cards.pdfAntimicrobial Game Cards.pdfGame Board Slide Show.pptxKey.pdfPostgame Survey.docxPre- and Posttest.docx [file mep_2374-8265.11565-s001.zip › H. Pre- and Posttest.docx]

**Pathogen Pursuit Pre- and Post-test**

What post-graduate year are you in?

| PGY-1 |  |
| --- | --- |
| PGY-2 |  |
| PGY-3 |  |

| ***Question*** | ***Answer*** |
| --- | --- |
| 1. Which of the following is an indication to obtain a head CT prior to an LP in suspected meningitis? 2. Headache 3. Immunocompromised 4. Photosensitivity 5. Suspected viral etiology |  |
| 1. Which of the following is an indication to empirically cover for *Pseudomonas* when treating for CAP? 2. Failure of outpatient antibiotics 3. Chronic dialysis 4. HIV 5. Recent hospital admission with IV antibiotics 6. Elevated A-a gradient |  |
| 1. A 32-year-old male asks about HIV prophylaxis. He is in a monogamous relationship with his husband who is HIV (+) with a CD4 count of 756 and an undetectable viral load. Which of the following is the most appropriate prophylaxis. 2. Tenofovir + Emtricitabine daily 3. Tenofovir + emtricitabine on the day of intercourse and for the next two days following 4. IM cabotegravir bimonthly 5. No prophylaxis indicated |  |
| 1. A 62-year-old male is evaluated in the ICU for ventilator associated pneumonia. He has been in the hospital for 15-days, originally admitted for cellulitis. Sputum cultures are pending. Which of the following is the most appropriate empiric antibiotic regimen? 2. Ertapenem, ciprofloxacin, and vancomycin 3. Meropenem and vancomycin 4. Meropenem, ciprofloxacin, and vancomycin 5. Piperacillin-tazobactam and linezolid |  |
| 1. A 56-year-old female with breast cancer currently undergoing chemotherapy is admitted for an acute herpes zoster infection affecting a single dermatome. What are the most appropriate isolation precautions? 2. No isolation 3. Contact precautions 4. Droplet precautions 5. Airborne and contact precautions |  |

Answer key:

1. B
2. D
3. D
4. C
5. D
